# Efficient Synthesis of Fully Substituted Pyrrolidine-Fused 3-Spirooxindoles via 1,3-Dipolar Cycloaddition of Aziridine and 3-Ylideneoxindole

**DOI:** 10.3390/molecules21091113

**Published:** 2016-08-24

**Authors:** Wen Ren, Qian Zhao, Chuan Zheng, Qiong Zhao, Li Guo, Wei Huang

**Affiliations:** 1 Key Laboratory of Drug Targeting and Drug Delivery Systems of Ministry of Education, Department of Medicinal Chemistry, West China School of Pharmacy, Sichuan University, Chengdu 610041, China; sklb_renwen@126.com; 2 State Key Laboratory Breeding Base of Systematic Research, Development and Utilization of Chinese Medicine, School of Pharmacy, Chengdu University of Traditional Chinese Medicine, Chengdu 611137, China; zhaoqianjy@126.com (Q.Z.); zhengchuan@cdutcm.edu.cn (C.Z.); zhaoqiongl@126.com (Q.Z.)

**Keywords:** spirooxindole-pyrrolidine, 1,3-dipolar cycloaddition, aziridine, single-step reaction

## Abstract

Drug-like spirocyclic scaffolds have been prepared by fusing fully functionalized pyrrolidine with oxindoles in an approach based on 1,3-dipolar cycloaddition. Reaction between aziridine and 3-ylideneoxindole generated diverse spirooxindole-pyrrolidines in good yield (up to 95%) with high diastereoselectivity (up to >20:1). The reaction also proceeded smoothly with several other synthetically useful activated trisubstituted olefins. The mild reaction conditions, short reaction times, and high tolerance for various substitutions make this approach attractive for constructing pharmacologically interesting spiro-architectures.

## 1. Introduction

Since the first report of Steven rearrangement in 1928 [1], nitrogen ylides have attracted considerable attention from organic chemists because of their distinctive properties and usefulness in preparative organic syntheses [2,3,4,5,6]. As nitrogen-based 1,3-dipoles, they play important roles in organic synthesis, particularly in cycloadditions [7,8,9,10]. Nitrogen ylides have been used to achieve (3 + 2) annulation [11,12,13,14,15] and (3 + 3) annulation [16,17], providing a direct route to various nitrogen-containing heterocycles, including pyrrolidine [18,19,20,21,22], piperidine [23,24], and piperazine [25]. This strategy has proven powerful for synthesizing natural products as well as other biologically interesting compounds [26,27,28,29].

**Figure 1:**
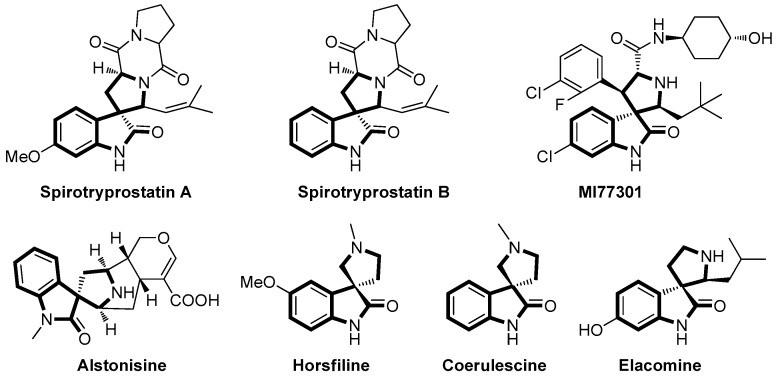
Examples of the spirooxindole-pyrrolidine framework in biologically active molecules.

Spirooxindole-pyrrolidine, a privileged framework with crucial biological activities, is present in a large family of alkaloid and natural products (Figure 1) [30,31,32,33]. For example, spirotryprostatins A and B, both isolated from *Aspergillus fumigatus*, completely inhibit progression from G2 to M phase in mammalian tsFT210 cells [30]. The synthetic analogues of these natural products are often more efficacious and selective than the natural molecules [34,35,36,37]. For example, spirooxindole-pyrrolidine derivative MI-77301, an inhibitor of murine double minute 2 (MDM2), entered its second Phase I clinical trial in 2013 [37].

**Scheme 1:**
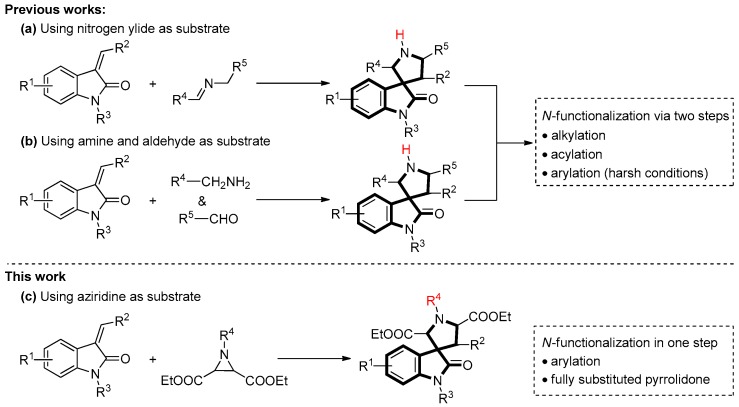
1,3-Dipolar cycloaddition-based synthesis of spirooxindole-pyrrolidines.

Various elegant studies have generated molecules with spirooxindole-pyrrolidine skeletons [38,39,40,41,42] using diverse reactions, including 1,3-dipolar cycloaddition [43,44,45,46,47,48,49,50,51,52], ring-enlargement [53,54,55], intramolecular Mannich reaction [56], rearrangement [57] and alkylation [58]. Nitrogen ylides have been used to synthesize spirooxindole-pyrrolidines via 1,3-dipolar addition; this process shows high reactivity, high yield, and excellent stereoselectivity (Scheme 1a [43,44,45,46,47] and Scheme 1b [48,49,50,51,52]). In this approach, two steps are required to modify the nitrogen by alkylation, acylation, or, under harsh conditions, arylation. Developing a one-step strategy for constructing spirooxindoles containing a fully substituted pyrrolidine remains an important challenge.

**Scheme 2:**
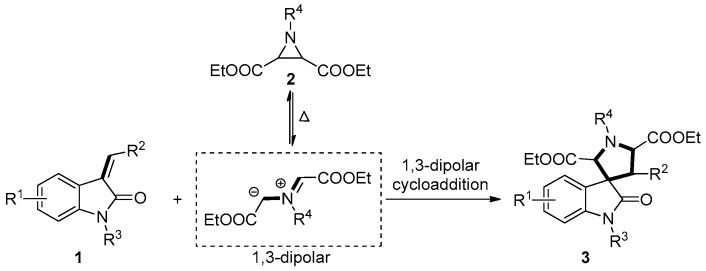
Synthetic strategy.

To construct functionalized spirooxindoles in a straightforward, single-step reaction, we envisioned 1,3-dipolar cycloaddition between 3-ylideneoxindole **1** and aziridine **2** (Scheme 1c). Aziridine could generate a 1,3-dipole through thermolysis, and then 1,3-dipolar cycloaddition of the 1,3-dipole with dipolarophile **1** would yield (3 + 2) cycloadduct **2** (Scheme 2). If successful, this approach would broaden the applications of aziridine and provide an alternative method for preparing pharmacologically interesting spirooxindole-pyrrolidines.

## 2. Results and Discussion

Our investigation began with 3-ylideneoxindole **1a** and 1,3-dipolar aziridine **2a** in MeCN at 90 °C (Table 1, entry 1). The 1,3-dipolar cycloaddition proceeded rapidly, affording **3a** in 40% yield. Encouraged by this result, we optimized the reaction conditions, first by replacing MeCN with other solvents while keeping other conditions the same (Table 1, entries 2–5). Toluene led to the best yield (Table 1, entry 3), but it did not improve the *dr* value. Varying temperature did not increase yield, though it did accelerate the reaction (Table 1, entries 6, 7). Therefore, we examined whether acid or alkali additive might promote the conversion from aziridine to 1,3-dipole and thereby increase yield (Table 1, entries 8–15) [59,60]. Among the acid additives tested, acetic acid afforded the highest yield (Table 1, entry 8), whereas no reaction was observed with trifluoroacetic acid (Table 1, entry 11). Base additives increased yield more than acid additives, with triethylamine giving the best result (Table 1, entry 15). When we carried out this model reaction under 90 °C using TEA as base additive, a similar good result was also obtained, but the reaction did not proceed quickly enough. Obviously, thermodynamic factor played an important role in the 1,3-dipole generation (Table 1, entry 16). Finally, we found that a different configuration of aziridine also participated in the reaction, giving the product **3a** in moderate yield (Table 1, entry 17).

**Figure 2:**
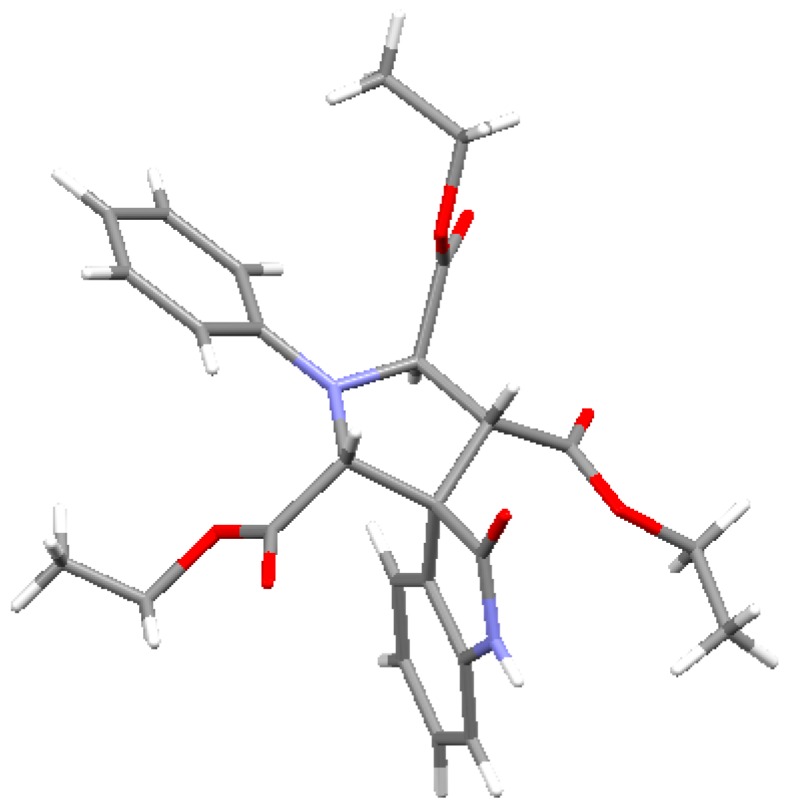
X-ray structure of **3a**.

Using the optimized reaction conditions, we evaluated the substrate scope and limitations of 1,3-dipolar cycloaddition (Table 2). We first examined the reaction of 1,3-dipolar aziridine **2a** with **1**. The nature of the functional group at position R^1^ on the oxindole core did not affect the reaction: both electron-deficient and electron-rich groups gave the corresponding products **3b**–**d** in high yield. Isopropoxyformoxyl, phenoxyformoxyl or dicyano groups at R^2^ barely affected reaction efficiency, leading to satisfying yields and *dr* values for the corresponding products **3e**–**g**. Aryl or polycyclic aryl groups at R^2^ afforded the products **3h**–**n** in moderate yield with high diastereoselectivity; yield was higher for electron-withdrawing substituents (Table 2, **3i**–**k**) than for electron-donating ones (Table 2, **3l**–**m**). A heteroaryl group at R^2^ led to the corresponding products **3o** and **3p** in slightly lower yield. Various protecting groups, including benzyl and Boc groups, were well tolerated and enhanced the *dr* values obtained for products **3q** and **3r**. Finally, we made substitutions on the aziridine **2** that led to good yield of products **3s** and **3t**. The relative configuration of product **3a** was unambiguously determined by X-ray crystallographic analysis (Figure 2) [61].

**Scheme 3:**
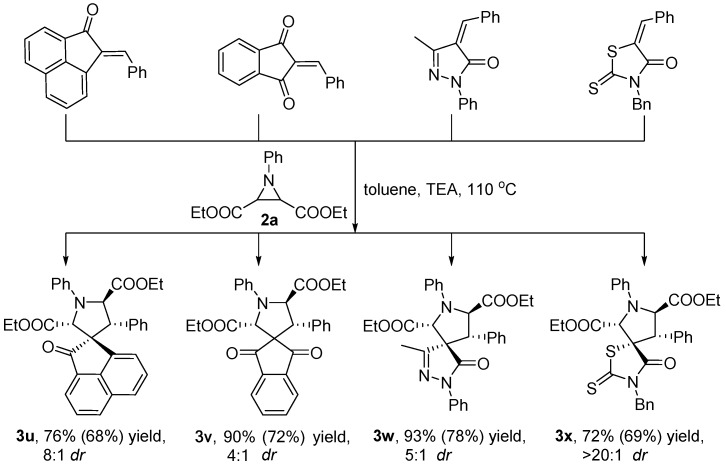
Synthesis of pharmacologically important spiro-pyrrolidines.

To further probe the usefulness of this single-step reaction, we examined whether it was compatible with synthetically useful activated trisubstituted olefins. The reaction proceeded smoothly with several such substrates, including olefinic acenaphthene, indenedione, pyrazolone, and rhodanine, providing the corresponding pharmacologically important spirocyclic products (Scheme 3, **3u**–**x**) in good yields (up to 95%) with moderate to high diastereoselectivities (up to >20:1).

## 3. Materials and Methods

### 3.1. General Information

NMR data was obtained for ^1^H at 400 MHz (Varian, Palo Alto, CA, USA) and for ^13^C at 100 MHz. Chemical shifts were reported in ppm from tetramethylsilane using solvent resonance in CDCl_3_ solution as the internal standard. ESI HRMS (Electrospray Ionization, High Resolution Mass Spectrum) was performed on a Waters SYNAPT G2 (Milford, MA, USA). Column chromatography was performed on silica gel (200–300 mesh) using an eluent of ethyl acetate and petroleum ether. TLC was performed on glass-backed silica plates; products were visualized using UV light and I_2_. Melting points were determined on a Mel-Temp apparatus (Electrothermal, Staffordshire, UK) and were not corrected. All chemicals were used from Adamas-beta (Adamas, Shanghai, China) without purification unless otherwise noted.

### 3.2. Synthesis

#### 3.2.1. General Procedure for the Synthesis of Spirooxindole-Pyrrolidines **3a**–**t**

A mixture of 3-ylideneoxindole **1** (1.1 mmol), aziridine **2** (1.0 mmol) and additive TEA (0.5 mmol) in toluene (2 mL) was refluxed at 110 °C under an open atmosphere. The reaction mixture stirred for a specified reaction time until most of 3-ylideneoxindole **1** was consumed (monitored by TLC). Then, the reaction mixture was concentrated and the residue was purified by elaborative chromatography on silica gel to give the final products **3a**–**t** in good yield (up to 77%) with high diastereoselectivity (up to >20:1). The products were further identified by ^1^H-NMR, ^13^C-NMR and HRMS (See supplementary materials).

*Triethyl-2-oxo-1′-phenylspiro(indoline-3,3′-pyrrolidine)-2′,4′,5′-tricarboxylate* (**3a**). The mixed two isomers were isolated by flash chromatography (petroleum ether/ethyl acetate = 5:1) in 78% yield (71.3 mg). The *dr* value was calculated to be 5:1 from crude ^1^H-NMR analysis of the mixture. After which, the pure major isomer **3a** was obtained as a white solid after elaborative chromatography (petroleum ether/ethyl acetate = 10:1) in 65% yield (59.4 mg). m.p. 130–132 °C; ^1^H-NMR (400 MHz, CDCl_3_) δ 8.04 (s, 1H), 7.35 (d, *J* = 7.2 Hz, 1H), 7.25–7.18 (m, 3H), 6.99 (t, *J* = 7.6 Hz, 1H), 6.85 (dd, *J* = 13.2, 7.6 Hz, 2H), 6.74 (d, *J* = 8.0 Hz, 2H), 5.42 (d, *J* = 8.4 Hz, 1H), 5.11 (s, 1H), 4.09–3.99 (m, 3H), 3.88–3.82 (m, 1H), 3.80–3.66 (m, 3H), 0.99 (t, *J* = 7.2 Hz, 3H), 0.79 (t, *J* = 7.2 Hz, 3H), 0.75 (t, *J* = 7.2 Hz, 3H); ^13^C-NMR (101 MHz, CDCl_3_) δ 176.14, 171.80, 167.35, 167.32, 145.26, 141.23, 129.59, 128.73, 126.28, 125.67, 122.71, 120.26, 116.39, 109.40, 68.76, 64.88, 61.45, 61.41, 61.08, 58.06, 54.64, 13.85, 13.49, 13.42; HRMS: *m*/*z* calcd. for C_26_H_28_N_2_O_7_ + Na, 503.1794; found, 503.1790.

*Triethyl-4-bromo-2-oxo-1′-phenylspiro(indoline-3,3′-pyrrolidine)-2′,4′,5′-tricarboxylate* (**3b**). The mixed two isomers were isolated by flash chromatography (petroleum ether/ethyl acetate = 5:1) in 82% yield (86.7 mg). The *dr* value was calculated to be 2.5:1 from crude ^1^H-NMR analysis of the mixture. The pure major isomer **3b** could not be separated in pure form after elaborative chromatography; the yield of **3b** was calculated to be 59% based on the total yield and *dr* value. m.p. 128–130 °C; ^1^H-NMR (400 MHz, CDCl_3_) δ 8.74 (s, 1H), 7.24–7.14 (m, 4H), 6.87–6.82 (m, 2H), 6.72 (d, *J* = 8.0 Hz, 2H), 5.49 (s, 1H), 5.40 (d, *J* = 8.8 Hz, 1H), 4.81 (d, *J* = 8.8 Hz, 1H), 4.18–4.08 (m, 4H), 4.06–4.02 (m, 2H), 1.14 (t, *J* = 7.2 Hz, 3H), 1.11 (t, *J* = 7.2 Hz, 3H), 0.89 (t, *J* = 7.2 Hz, 3H); ^13^C-NMR (101 MHz, CDCl_3_) δ 176.33, 172.46, 168.35, 168.22, 144.79, 144.09, 130.81, 128.81, 128.67, 127.02, 119.60, 118.46, 116.37, 115.42, 109.43, 64.93, 64.31, 61.69, 61.40, 58.94, 51.01, 14.01, 13.68, 13.62; HRMS: *m*/*z* calcd. for C_26_H_27_BrN_2_O_7_ + Na, 581.0899; found, 581.0901.

*Triethyl-5-fluoro-2-oxo-1′-phenylspiro(indoline-3,3′-pyrrolidine)-2′,4′,5′-tricarboxylate* (**3c**). The mixed two isomers were isolated by flash chromatography (petroleum ether/ethyl acetate = 5:1) in 83% yield (78.2 mg). The *dr* value was calculated to be 4:1 from crude ^1^H-NMR analysis of the mixture. After which, the pure major isomer **3c** was obtained as a white solid after elaborative chromatography (petroleum ether/ethyl acetate = 10:1) in 67% yield (62.6 mg). m.p. 120–122 °C; ^1^H-NMR (400 MHz, CDCl_3_) δ 8.90 (s, 1H), 7.22 (t, *J* = 8.0 Hz, 2H), 7.13 (dd, *J* = 8.0, 2.4 Hz, 1H), 6.99–6.92 (m, 1H), 6.89–6.84 (m, 2H), 6.76 (d, *J* = 8.0 Hz, 2H), 5.38 (d, *J* = 8.0 Hz, 1H), 5.11 (s, 1H), 4.09–4.01 (m, 3H), 3.94–3.72 (m, 4H), 1.01 (t, *J* = 7.2 Hz, 3H), 0.85 (t, *J* = 7.2 Hz, 3H), 0.78 (t, *J* = 7.2 Hz, 3H); ^13^C-NMR (101 MHz, CDCl_3_) δ 176.48, 171.68, 167.25, 167.13, 158.64 (d, *J*_CF_ = 243.4 Hz), 145.09, 137.56 (d, *J*_CF_ = 2.0 Hz), 128.78, 127.28 (d, *J*_CF_ = 8.1 Hz), 120.59, 116.60, 116.12 (d, *J*_CF_ = 23.2 Hz), 114.21 (d, *J*_CF_ = 25.3 Hz), 110.26 (d, *J*_CF_ = 8.1 Hz), 68.70, 64.70, 61.59, 61.54, 61.21, 58.60, 54.52, 13.86, 13.50, 13.48; HRMS: *m*/*z* calcd. for C_26_H_27_FN_2_O_7_ + Na, 521.1700; found, 521.1696.

*Triethyl-5-methyl-2-oxo-1′-phenylspiro(indoline-3,3′-pyrrolidine)-2′,4′,5′-tricarboxylate* (**3d**). The mixed two isomers were isolated by flash chromatography (petroleum ether/ethyl acetate = 5:1) in 81% yield (75.7 mg). The *dr* value was calculated to be 6:1 from crude ^1^H-NMR analysis of the mixture. After which, the pure major isomer **3d** was obtained as a white solid after elaborative chromatography (petroleum ether/ethyl acetate = 10:1) in 69% yield (64.9 mg). m.p. 120–123 °C; ^1^H-NMR (400 MHz, CDCl_3_) δ 8.61 (s, 1H), 7.21 (dd, *J* = 8.4, 7.6 Hz, 2H), 7.15 (s, 1H), 7.02 (dd, *J* = 8.0, 0.8 Hz, 1H), 6.84 (t, *J* = 7.2 Hz, 1H), 6.79–6.74 (m, 3H), 5.42 (d, *J* = 8.8 Hz, 1H), 5.11 (s, 1H), 4.10–4.01 (m, 3H), 3.90–3.82 (m, 1H), 3.78–3.74 (m, 1H), 3.72–3.69 (m, 2H), 2.27 (s, 3H), 1.00 (t, *J* = 7.2 Hz, 3H), 0.80 (t, *J* = 7.2 Hz, 3H), 0.75 (t, *J* = 7.2 Hz, 3H); ^13^C-NMR (101 MHz, CDCl_3_) δ 176.60, 171.87, 167.38, 167.34, 145.31, 138.99, 132.18, 129.90, 128.74, 126.73, 125.68, 120.09, 116.19, 109.32, 68.71, 64.90, 61.44, 61.37, 61.02, 58.23, 54.68, 21.09, 13.86, 13.48, 13.40; HRMS: *m*/*z* calcd. for C_27_H_30_N_2_O_7_ + Na, 517.1951; found, 517.1954.

*2′,5′-Diethyl-4′-isopropyl-2-oxo-1′-phenylspiro(indoline-3,3′-pyrrolidine)-2′,4′,5′-tricarboxylate* (**3e**). The mixed two isomers were isolated by flash chromatography (petroleum ether/ethyl acetate = 5:1) in 81% yield (76.3 mg). The *dr* value was calculated to be 4:1 from crude ^1^H-NMR analysis of the mixture. After which, the pure major isomer **3e** was obtained as a white solid after elaborative chromatography (petroleum ether/ethyl acetate = 10:1) in 65% yield (61.2 mg). m.p. 110–115 °C; ^1^H-NMR (400 MHz, CDCl_3_) δ 8.27 (d, *J* = 7.2 Hz, 1H), 7.92 (s, 1H), 7.25–7.20 (m, 3H), 7.05 (td, *J* = 7.6, 0.8 Hz, 1H), 6.89–6.83 (m, 2H), 6.67 (d, *J* = 8.0 Hz, 2H), 4.97 (d, *J* = 10.8 Hz, 1H), 4.70 (s, 1H), 4.66–4.58 (m, 1H), 4.50 (d, *J* = 10.8 Hz, 1H), 4.48–4.40 (m, 1H), 4.30–4.21 (m, 1H), 4.16–4.04 (m, 2H), 1.37 (t, *J* = 7.2 Hz, 3H), 1.13 (t, *J* = 7.2 Hz, 3H), 1.03 (d, *J* = 6.4 Hz, 3H), 0.56 (d, *J* = 6.4 Hz, 3H). ^13^C-NMR (101 MHz, CDCl_3_) δ 175.43, 171.37, 169.77, 166.74, 145.38, 140.19, 130.54, 129.42, 129.03, 126.41, 123.49, 119.91, 114.74, 109.18, 71.32, 69.04, 62.16, 61.54, 61.42, 57.63, 52.95, 21.42, 20.56, 14.04, 13.99; HRMS: *m*/*z* calcd. for C_27_H_30_N_2_O_7_ + Na, 517.1951; found, 517.1948.

*2′,5′-Diethyl-4′-phenyl-2-oxo-1′-phenylspiro(indoline-3,3′-pyrrolidine)-2′,4′,5′-tricarboxylate* (**3f**). The mixed two isomers were isolated by flash chromatography (petroleum ether/ethyl acetate = 5:1) in 87% yield (86.9 mg). The *dr* value was calculated to be 4:1 from crude ^1^H-NMR analysis of the mixture. After which, the pure major isomer **3f** was obtained as a white solid after elaborative chromatography (petroleum ether/ethyl acetate = 10:1) in 70% yield (69.5 mg). m.p. 135–137 °C; ^1^H-NMR (400 MHz, CDCl_3_) δ 8.68 (s, 1H), 7.46 (d, *J* = 7.2 Hz, 1H), 7.30 (td, *J* = 7.6, 0.8 Hz, 1H), 7.23–7.14 (m, 4H), 7.13–7.05 (m, 2H), 6.91–6.84 (m, 2H), 6.78 (d, *J* = 8.0 Hz, 2H), 6.28–6.25 (m, 2H), 5.51 (d, *J* = 8.0 Hz, 1H), 5.19 (s, 1H), 4.30 (d, *J* = 8.0 Hz, 1H), 4.11–4.03 (m, 2H), 3.75–3.65 (m, 2H), 1.01 (t, *J* = 7.2 Hz, 3H), 0.74 (t, *J* = 7.2 Hz, 3H); ^13^C-NMR (101 MHz, CDCl_3_) δ 176.29, 171.74, 167.25, 166.36, 149.72, 145.16, 141.62, 129.88, 129.33, 128.80, 126.46, 126.20, 125.62, 122.97, 120.96, 120.50, 116.59, 109.99, 68.88, 64.87, 61.60, 61.20, 58.22, 54.62, 13.88, 13.50; HRMS: *m*/*z* calcd. for C_30_H_28_N_2_O_7_ + Na, 551.1794; found, 551.1798.

*Diethyl-4′,4′-dicyano-2-oxo-1′-phenylspiro(indoline-3,3′-pyrrolidine)-2′,5′-dicarboxylate* (**3g**). The mixed two isomers were isolated by flash chromatography (petroleum ether/ethyl acetate = 5:1) in 86% yield (75.3 mg). The *dr* value was calculated to be 4:1 from crude ^1^H-NMR analysis of the mixture. After which, the pure major isomer **3g** was obtained as a white solid after elaborative chromatography (petroleum ether/ethyl acetate = 10:1) in 69% yield (60.2 mg). m.p. 140–142 °C; ^1^H-NMR (400 MHz, CDCl_3_) δ 8.72 (s, 1H), 7.46 (d, *J* = 7.6 Hz, 1H), 7.38 (t, *J* = 7.6 Hz, 1H), 7.29–7.25 (m, 2H), 7.13 (t, *J* = 7.6 Hz, 1H), 7.02 (d, *J* = 7.6 Hz, 1H), 6.95 (t, *J* = 7.6 Hz, 1H), 6.74 (t, *J* = 8.0 Hz, 2H), 5.44 (s, 1H), 5.23 (s, 1H), 4.36–4.24 (m, 2H), 3.83 (q, *J* = 7.2 Hz, 2H), 1.25–1.21(m, 3H), 0.78 (t, *J* = 7.2 Hz, 3H); ^13^C-NMR (101 MHz, CDCl_3_) δ 172.46, 166.81, 165.21, 143.61, 141.50, 131.86, 129.18, 126.81, 123.48, 121.60, 121.55, 116.69, 111.71, 111.27, 109.85, 69.27, 66.25, 63.14, 61.89, 59.31, 45.06, 13.82, 13.46; HRMS: *m*/*z* calcd. for C_25_H_22_N_4_O_5_ + Na, 481.1488; found, 481.1489.

*Diethyl-2-oxo-1′,4′-diphenylspiro(indoline-3,3′-pyrrolidine)-2′,5′-dicarboxylate* (**3h**). The mixed two isomers were isolated by flash chromatography (petroleum ether/ethyl acetate = 5:1) in 63% yield (57.6 mg). The *dr* value was calculated to be >20:1 from crude ^1^H-NMR analysis of the mixture. After which, the pure major isomer **3h** was obtained as a white solid after elaborative chromatography (petroleum ether/ethyl acetate = 10:1) in 61% yield (55.8 mg). m.p. 130–132 °C; ^1^H-NMR (400 MHz, CDCl_3_) δ 8.34 (s, 1H), 7.48 (d, *J* = 7.2 Hz, 1H), 7.21 (dd, *J* = 8.4, 7.6 Hz, 2H), 7.12–7.04 (m, 6H), 7.01–6.97 (m, 1H), 6.81 (t, *J* = 7.2 Hz, 1H), 6.73 (d, *J* = 8.0 Hz, 2H), 6.63 (d, *J* = 7.6 Hz, 1H), 5.39 (d, *J* = 10.4 Hz, 1H), 5.33 (s, 1H), 4.30 (d, *J* = 10.4 Hz, 1H), 4.00–3.89 (m, 2H), 3.80–3.65 (m, 2H), 0.86 (t, *J* = 7.2 Hz, 3H), 0.68 (t, *J* = 7.2 Hz, 3H); ^13^C-NMR (101 MHz, CDCl_3_) δ 176.48, 171.79, 167.85, 145.42, 140.58, 132.31, 129.10, 128.77, 128.34, 128.04, 128.01, 126.86, 125.52, 122.24, 119.40, 115.35, 109.64, 67.54, 67.20, 61.68, 61.26, 60.93, 57.21, 13.79, 13.46; HRMS: *m*/*z* calcd. for C_29_H_28_N_2_O_5_ + Na, 507.1896; found, 507.1900.

*Diethyl-4′-(4-bromophenyl)-2-oxo-1′-phenylspiro(indoline-3,3′-pyrrolidine)-2′,5′-dicarboxylate* (**3i**). The mixed two isomers were isolated by flash chromatography (petroleum ether/ethyl acetate = 5:1) in 68% yield (73.2 mg). The *dr* value was calculated to be 6:1 from crude ^1^H-NMR analysis of the mixture. After which, the pure major isomer **3i** was obtained as a white solid after elaborative chromatography (petroleum ether/ethyl acetate = 10:1) in 58% yield (62.7 mg). m.p. 79–82 °C; ^1^H-NMR (400 MHz, CDCl_3_) δ 8.20 (s, 1H), 7.47 (d, *J* = 7.6 Hz, 1H), 7.24–7.20 (m, 4H), 7.14 (t, *J* = 7.6 Hz, 1H), 7.02–6.94 (m, 3H), 6.83 (t, *J* = 7.2 Hz, 1H), 6.71 (d, *J* = 8.0 Hz, 2H), 6.66 (d, *J* = 7.6 Hz, 1H), 5.33–5.31 (m, 2H), 4.24 (d, *J* = 10.4 Hz, 1H), 3.99–3.93 (m, 2H), 3.80–3.65 (m, 2H), 0.88 (t, *J* = 7.2 Hz, 3H), 0.68 (t, *J* = 7.2 Hz, 3H); ^13^C-NMR (101 MHz, CDCl_3_) δ 176.26, 171.62, 167.74, 145.25, 140.61, 131.41, 131.27, 130.02, 129.37, 128.81, 126.72, 125.12, 122.38, 122.24, 119.55, 115.34, 109.95, 67.49, 67.16, 61.46, 61.42, 61.02, 56.59, 13.82, 13.45; HRMS: *m*/*z* calcd. for C_29_H_27_BrN_2_O_5_ + Na, 585.1001; found, 585.1003.

*Diethyl-4′-(2-fluorophenyl)-2-oxo-1′-phenylspiro(indoline-3,3′-pyrrolidine)-2′,5′-dicarboxylate* (**3j**). The mixed two isomers were isolated by flash chromatography (petroleum ether/ethyl acetate = 5:1) in 66% yield (63.1 mg). The *dr* value was calculated to be >20:1 from crude ^1^H-NMR analysis of the mixture. After which, the pure major isomer **3j** was obtained as a white solid after elaborative chromatography (petroleum ether/ethyl acetate = 10:1) in 63% yield (60.2 mg). m.p. 137–140 °C; ^1^H-NMR (400 MHz, CDCl_3_) δ 8.46 (s, 1H), 7.39 (d, *J* = 7.6 Hz, 1H), 7.22 (t, *J* = 8.0 Hz, 2H), 7.16–7.11 (m, 2H), 7.09–7.06 (m, 1H), 6.95 (t, *J* = 7.6 Hz, 1H), 6.88–6.80 (m, 3H), 6.73 (d, *J* = 8.4 Hz, 2H), 6.69 (d, *J* = 8.0 Hz, 1H), 5.41 (d, *J* = 9.6 Hz, 1H), 5.34 (s, 1H), 4.66 (d, *J* = 9.6 Hz, 1H), 4.04–3.92 (m, 2H), 3.79–3.66 (m, 2H), 0.88 (t, *J* = 7.2 Hz, 3H), 0.68 (t, *J* = 7.2 Hz, 3H); ^13^C-NMR (101 MHz, CDCl_3_) δ 176.82, 171.65, 167.92, 161.00 (d, *J*_CF_ = 249.5 Hz), 145.44, 141.01, 129.78 (d, *J*_CF_ = 3.0 Hz), 129.55 (d, *J*_CF_ = 9.1 Hz), 129.21, 128.81, 126.98, 125.44, 123.60 (d, *J*_CF_ = 3.0 Hz), 122.04, 120.17 (d, *J*_CF_ = 14.1 Hz), 119.51, 115.46, 115.39 (d, *J*_CF_ = 23.2 Hz), 109.71, 67.75, 67.11, 61.32, 60.98, 60.78, 49.67, 13.75, 13.43; HRMS: *m*/*z* calcd. for C_29_H_27_FN_2_O_5_ + Na; 525.1802; found, 525.1804.

*Diethyl-4′-(4-nitrophenyl)-2-oxo-1′-phenylspiro(indoline-3,3′-pyrrolidine)-2′,5′-dicarboxylate* (**3k**). The mixed two isomers were isolated by flash chromatography (petroleum ether/ethyl acetate = 5:1) in 62% yield (62.8 mg). The *dr* value was calculated to be 5:1 from crude ^1^H-NMR analysis of the mixture. After which, the pure major isomer **3k** was obtained as a white solid after elaborative chromatography (petroleum ether/ethyl acetate = 10:1) in 52% yield (52.3 mg). m.p. 90–93 °C; ^1^H-NMR (400 MHz, CDCl_3_) δ 7.95 (d, *J* = 8.8 Hz, 2H), 7.94–7.89 (m, 1H), 7.48 (d, *J* = 7.6 Hz, 1H), 7.29 (d, *J* = 8.8 Hz, 2H), 7.24 (d, *J* = 8.0 Hz, 2H), 7.14 (t, *J* = 7.6 Hz, 1H), 7.02 (t, *J* = 7.6 Hz, 1H), 6.85 (t, *J* = 7.2 Hz, 1H), 6.73 (d, *J* = 8.0 Hz, 2H), 6.65 (d, *J* = 7.6 Hz, 1H), 5.41 (d, *J* = 10.0 Hz, 1H), 5.34 (s, 1H), 4.38 (d, *J* = 10.4 Hz, 1H), 4.02–3.92 (m, 2H), 3.82–3.67 (m, 2H), 0.88 (t, *J* = 7.2 Hz, 3H), 0.69 (t, *J* = 7.2 Hz, 3H); ^13^C-NMR (101 MHz, CDCl_3_) δ 175.72, 171.33, 167.55, 147.58, 145.05, 140.37, 140.00, 129.69, 129.31, 128.88, 126.70, 124.65, 123.26, 122.61, 119.84, 115.42, 109.97, 67.58, 66.90, 61.63, 61.41, 61.13, 56.49, 13.81, 13.46; HRMS: *m*/*z* calcd. for C_29_H_27_N_3_O_7_ + Na, 552.1747; found, 552.1744.

*Diethyl-2-oxo-1′-phenyl-4′-(p-tolyl)spiro(indoline-3,3′-pyrrolidine)-2′,5′-dicarboxylate* (**3l**). The mixed two isomers were isolated by flash chromatography (petroleum ether/ethyl acetate = 5:1) in 58% yield (55.3 mg). The *dr* value was calculated to be >20:1 from crude ^1^H-NMR analysis of the mixture. After which, the pure major isomer **3l** was obtained as a white solid after elaborative chromatography (petroleum ether/ethyl acetate = 10:1) in 56% yield (53.4 mg). m.p. 85–88 °C; ^1^H-NMR (400 MHz, CDCl_3_) δ 8.14 (s, 1H), 7.49 (d, *J* = 7.6 Hz, 1H), 7.21 (t, *J* = 7.6 Hz, 2H), 7.11 (t, *J* = 7.6 Hz, 1H), 7.00 (t, *J* = 7.6 Hz, 1H), 6.95–6.80 (m, 5H), 6.73 (d, *J* = 8.4 Hz, 2H), 6.63 (d, *J* = 8.0 Hz, 1H), 5.35 (d, *J* = 10.4 Hz, 1H), 5.31 (s, 1H), 4.25 (d, *J* = 10.0 Hz, 1H), 4.00–3.89 (m, 2H), 3.80–3.65 (m, 2H), 2.13 (s, 3H), 0.86 (t, *J* = 7.2 Hz, 3H), 0.68 (t, *J* = 7.2 Hz, 3H); ^13^C-NMR (101 MHz, CDCl_3_) δ 176.55, 171.87, 167.89, 145.47, 140.63, 137.59, 132.16, 129.25, 129.06, 128.76, 127.83, 126.92, 125.33, 122.14, 119.38, 115.37, 109.69, 67.54, 67.44, 61.68, 61.23, 60.92, 57.20, 21.24, 13.79, 13.46; HRMS: *m*/*z* calcd. for C_30_H_30_N_2_O_5_ + Na, 521.2052; found, 521.2056.

*Diethyl-4′-(3,4-dimethoxyphenyl)-2-oxo-1′-phenylspiro(indoline-3,3′-pyrrolidine)-2′,5′-dicarboxylate* (**3m**). The mixed two isomers were isolated by flash chromatography (petroleum ether/ethyl acetate = 5:1) in 57% yield (59.4 mg). The *dr* value was calculated to be 4:1 from crude ^1^H-NMR analysis of the mixture. After which, the pure major isomer **3m** was obtained as a white solid after elaborative chromatography (petroleum ether/ethyl acetate = 10:1) in 46% yield (47.5 mg). m.p. 100–103 °C; ^1^H-NMR (400 MHz, CDCl_3_) δ 7.52 (d, *J* = 7.2 Hz, 1H), 7.28 (s, 1H), 7.22 (dd, *J* = 8.0, 7.6 Hz, 2H), 7.14 (t, *J* = 7.6 Hz, 1H), 7.02 (t, *J* = 7.6 Hz, 1H), 6.82 (t, *J* = 7.6 Hz, 1H), 6.72 (d, *J* = 8.0 Hz, 2H), 6.66–6.63 (m, 2H), 6.57 (d, *J* = 8.0 Hz, 1H), 6.46 (d, *J* = 2.0 Hz, 1H), 5.30 (s, 1H), 5.24 (d, *J* = 10.4 Hz, 1H), 4.22 (d, *J* = 10.4 Hz, 1H), 4.02–3.91 (m, 2H), 3.83–3.79 (m, 1H), 3.75 (s, 3H), 3.71–3.67 (m, 1H), 3.61 (s, 3H), 0.88 (t, *J* = 7.2 Hz, 3H), 0.69 (t, *J* = 7.2 Hz, 3H); ^13^C-NMR (101 MHz, CDCl_3_) δ 175.17, 172.71, 168.64, 148.59, 148.03, 145.23, 141.06, 129.07, 128.81, 128.74, 124.30, 123.10, 122.96, 120.62, 118.92, 114.75, 111.36, 110.49, 109.64, 68.45, 65.15, 61.66, 61.29, 61.13, 58.39, 55.60, 55.49, 13.98, 13.64; HRMS: *m*/*z* calcd. for C_31_H_32_N_2_O_7_ + Na, 567.2107; found, 567.2110.

*Diethyl-4′-(naphthalen-2-yl)-2-oxo-1′-phenylspiro(indoline-3,3′-pyrrolidine)-2′,5′-dicarboxylate* (**3n**). The mixed two isomers were isolated by flash chromatography (petroleum ether/ethyl acetate = 5:1) in 55% yield (55.7 mg). The *dr* value was calculated to be >20:1 from crude ^1^H-NMR analysis of the mixture. After which, the pure major isomer **3n** was obtained as a white solid after elaborative chromatography (petroleum ether/ethyl acetate = 10:1) in 53% yield (53.5 mg). m.p. 105–107 °C; ^1^H-NMR (400 MHz, CDCl_3_) δ 8.02 (s, 1H), 7.67–7.65 (m, 2H), 7.59–7.51 (m, 3H), 7.41–7.37 (m, 2H), 7.24–7.14 (m, 3H), 7.07–6.99 (m, 2H), 6.82 (t, *J* = 7.2 Hz, 1H), 6.75 (d, *J* = 8.0 Hz, 2H), 6.53 (d, *J* = 7.2 Hz, 1H), 5.50 (d, *J* = 10.4 Hz, 1H), 5.37 (s, 1H), 4.47 (d, *J* = 10.0 Hz, 1H), 3.96–3.88 (m, 2H), 3.81–3.62 (m, 2H), 0.83 (t, *J* = 7.2 Hz, 3H), 0.66 (t, *J* = 7.2 Hz, 3H); ^13^C-NMR (101 MHz, CDCl_3_) δ 176.39, 171.83, 167.84, 145.44, 140.61, 132.89, 132.87, 129.93, 129.18, 128.79, 128.14, 127.96, 127.62, 127.44, 126.91, 126.12, 126.02, 125.71, 125.47, 122.25, 119.46, 115.41, 109.78, 67.62, 67.48, 61.75, 61.31, 60.96, 57.42, 13.80, 13.46; HRMS: *m*/*z* calcd. for C_33_H_30_N_2_O_5_ + Na, 557.2052; found, 557.2049.

*Diethyl-4′-(furan-2-yl)-2-oxo-1′-phenylspiro(indoline-3,3′-pyrrolidine)-2′,5′-dicarboxylate* (**3o**). The mixed two isomers were isolated by flash chromatography (petroleum ether/ethyl acetate = 5:1) in 46% yield (41.3 mg). The *dr* value was calculated to be 3:1 from crude ^1^H-NMR analysis of the mixture. After which, the pure major isomer **3o** was obtained as a white solid after elaborative chromatography (petroleum ether/ethyl acetate = 10:1) in 35% yield (31.1 mg). m.p. 140–143 °C; ^1^H-NMR (400 MHz, CDCl_3_) δ 8.41 (s, 1H), 7.34 (d, *J* = 7.6 Hz, 1H), 7.22 (t, *J* = 8.0 Hz, 2H), 7.15 (t, *J* = 7.6 Hz, 1H), 7.05 (s, 1H), 6.95 (t, *J* = 7.6 Hz, 1H), 6.85–6.77 (m, 2H), 6.72 (d, *J* = 8.0 Hz, 2H), 6.07–6.01 (m, 2H), 5.27 (d, *J* = 10.0 Hz, 1H), 5.25 (s, 1H), 4.43 (d, *J* = 9.6 Hz, 1H), 4.11–3.94 (m, 2H), 3.79–3.66 (m, 2H), 0.94 (t, *J* = 7.2 Hz, 3H), 0.71 (t, *J* = 7.2 Hz, 3H); ^13^C-NMR (101 MHz, CDCl_3_) δ 176.58, 171.71, 167.64, 147.90, 145.28, 142.35, 140.79, 129.15, 128.80, 126.74, 125.63, 122.31, 119.66, 115.53, 110.03, 109.57, 107.99, 67.67, 66.83, 61.47, 61.02, 60.03, 50.42, 13.81, 13.48; HRMS: *m*/*z* calcd. for C_27_H_26_N_2_O_6_ + Na, 497.1689; found, 497.1687.

*Diethyl-2-oxo-1′-phenyl-4′-(thiophen-2-yl)spiro(indoline-3,3′-pyrrolidine)-2′,5′-dicarboxylate* (**3p**). The mixed two isomers were isolated by flash chromatography (petroleum ether/ethyl acetate = 5:1) in 52% yield (48.1 mg). The *dr* value was calculated to be 3:1 from crude ^1^H-NMR analysis of the mixture. After which, the pure major isomer **3p** was obtained as a white solid after elaborative chromatography (petroleum ether/ethyl acetate = 10:1) in 39% yield (36.2 mg). m.p. 192–194 °C; ^1^H-NMR (400 MHz, CDCl_3_) δ 8.14 (s, 1H), 7.48 (d, *J* = 7.6 Hz, 1H), 7.23–7.18 (m, 3H), 7.06–7.01 (m, 2H), 6.84–6.70 (m, 6H), 5.29 (s, 1H), 5.17 (d, *J* = 10.4 Hz, 1H), 4.59 (d, *J* = 10.0 Hz, 1H), 4.05–3.93 (m, 2H), 3.82–3.67 (m, 2H), 0.90 (t, *J* = 7.2 Hz, 3H), 0.70 (t, *J* = 7.2 Hz, 3H); ^13^C-NMR (101 MHz, CDCl_3_) δ 176.14, 171.42, 167.67, 145.21, 141.10, 134.68, 129.51, 128.77, 127.24, 127.05, 126.19, 125.70, 125.39, 122.54, 119.60, 115.44, 109.82, 69.71, 67.23, 61.41, 61.21, 61.01, 53.06, 13.82, 13.47; HRMS: *m*/*z* calcd. for C_27_H_26_N_2_O_5_S + Na, 513.1460; found, 513.1458.

*Triethyl-1-benzyl-2-oxo-1′-phenylspiro(indoline-3,3′-pyrrolidine)-2′,4′,5′-tricarboxylate* (**3q**). The mixed two isomers were isolated by flash chromatography (petroleum ether/ethyl acetate = 5:1) in 88% yield (95.5 mg). The *dr* value was calculated to be 7:1 from crude ^1^H-NMR analysis of the mixture. After which, the pure major isomer **3q** was obtained as a white solid after elaborative chromatography (petroleum ether/ethyl acetate = 10:1) in 77% yield (83.6 mg). m.p. 170–172 °C; ^1^H-NMR (400 MHz, CDCl_3_) δ 7.48 (d, *J* = 8.0 Hz, 2H), 7.36–7.34 (m, 3H), 7.31–7.29 (m, 1H), 7.24–7.16 (m, 3H), 6.96 (t, *J* = 7.6 Hz, 1H), 6.84 (t, *J* = 7.2 Hz, 1H), 6.76 (t, *J* = 8.4 Hz 3H), 5.44 (dd, *J* = 8.0, 1.0 Hz, 1H), 5.18 (s, 1H), 4.98 (d, *J* = 5.2 Hz, 2H), 4.10 (d, *J* = 8.4 Hz, 1H), 4.06–4.02 (m, 2H), 3.79–3.75 (m, 1H), 3.64–3.55 (m, 3H), 1.01–0.98 (m, 3H), 0.56–0.50 (m, 6H); ^13^C-NMR (101 MHz, CDCl_3_) δ 174.83, 171.78, 167.32, 167.28, 145.35, 143.43, 135.68, 129.38, 128.72, 128.68, 128.08, 127.92, 125.95, 125.38, 122.68, 120.20, 116.41, 108.67, 68.94, 65.08, 61.40, 60.99, 57.50, 54.78, 44.65, 22.66, 13.86, 13.30, 13.24; HRMS: *m*/*z* calcd. for C_33_H_34_N_2_O_7_ + Na, 593.2264; found, 593.2266.

*1-(Tert-butyl)-2′,4′,5′-triethyl-2-oxo-1′-phenylspiro(indoline-3,3′-pyrrolidine)-1,2′,4′,5′-tetracarboxylate* (**3r**). The mixed two isomers were isolated by flash chromatography (petroleum ether/ethyl acetate = 5:1) in 82% yield (90.3 mg). The *dr* value was calculated to be 6:1 from crude ^1^H-NMR analysis of the mixture. After which, the pure major isomer **3r** was obtained as a white solid after elaborative chromatography (petroleum ether/ethyl acetate = 10:1) in 71% yield (77.4 mg). m.p. 150–153 °C; ^1^H-NMR (400 MHz, CDCl_3_) δ 7.86 (d, *J* = 8.4 Hz, 1H), 7.40 (dd, *J* = 7.6, 0.8 Hz, 1H), 7.35–7.30 (m, 1H), 7.21 (dd, *J* = 8.0, 7.2 Hz, 2H), 7.12 (dd, *J* = 8.0, 7.6 Hz, 1H), 6.86 (t, *J* = 7.6 Hz, 1H), 6.74 (d, *J* = 8.0 Hz, 2H), 5.39 (d, *J* = 8.0 Hz, 1H), 5.14 (s, 1H), 4.08–3.96 (m, 3H), 3.78–3.65 (m, 4H), 1.68 (s, 9H), 0.97 (t, *J* = 6.8 Hz, 3H), 0.81–0.76 (m, 6H); ^13^C-NMR (101 MHz, CDCl_3_) δ 173.34, 171.55, 167.06, 166.75, 149.01, 145.13, 140.31, 129.80, 129.21, 128.73, 125.31, 124.64, 120.07, 116.70, 114.59, 84.73, 69.08, 64.90, 61.88, 61.49, 61.12, 58.18, 55.31, 42.99, 28.10, 14.15, 13.83, 13.38, 13.26; HRMS: *m*/*z* calcd. for C_31_H_36_N_2_O_9_ + Na, 603.2319; found, 603.2314.

*Diethyl-2-oxo-4′-phenyl-1′-(m-tolyl)spiro(indoline-3,3′-pyrrolidine)-2′,5′-dicarboxylate* (**3s**). The mixed two isomers were isolated by flash chromatography (petroleum ether/ethyl acetate = 5:1) in 70% yield (62.3 mg). The dr value was calculated to be 5:1 from crude ^1^H-NMR analysis of the mixture. After which, the pure major isomer **3s** was obtained as a white solid after elaborative chromatography (petroleum ether/ethyl acetate = 10:1) in 58% yield (52.1 mg). m.p. 135–138 °C; ^1^H-NMR (400 MHz, CDCl_3_) δ 8.73 (s, 1H), 7.35 (d, *J* = 7.6 Hz, 1H), 7.23 (t, *J* = 8.0 Hz, 1H), 7.08 (t, *J* = 7.6 Hz, 1H), 6.99 (t, *J* = 7.6 Hz, 1H), 6.90 (d, *J* = 7.6 Hz, 1H), 6.66 (d, *J* = 7.6 Hz, 1H), 6.61 (s, 1H), 6.52 (d, *J* = 8.0 Hz, 1H), 5.41 (d, *J* = 8.4 Hz, 1H), 5.11 (s, 1H), 4.09–4.02 (m, 3H), 3.88–3.84 (m, 1H), 3.77–3.68 (m, 3H), 2.27 (s, 3H), 1.02 (t, *J* = 7.2 Hz, 3H), 0.81–0.74 (m, 6H); ^13^C-NMR (101 MHz, CDCl_3_) δ 176.66, 171.99, 167.45, 167.38, 145.19, 141.42, 138.41, 129.59, 128.56, 126.21, 125.70, 122.69, 121.19, 117.19, 113.43, 109.63, 68.72, 64.81, 61.42, 61.22, 61.07, 58.14, 54.64, 21.65, 13.90, 13.49, 13.40; HRMS: *m*/*z* calcd. for C_27_H_30_N_2_O_7_ + Na, 517.1951; found, 517.1954.

*Diethyl-2-oxo-4′-phenyl-1′-(4-(trifluoromethyl)phenyl)spiro(indoline-3,3′-pyrrolidine)-2′,5′-dicarboxylate* (**3t**). The mixed two isomers were isolated by flash chromatography (petroleum ether/ethyl acetate = 5:1) in 67% yield (55.4 mg). The dr value was calculated to be 5:1 from crude ^1^H-NMR analysis of the mixture. After which, the pure major isomer **3t** was obtained as a white solid after elaborative chromatography (petroleum ether/ethyl acetate = 10:1) in 56% yield (46.2 mg). m.p. 145–147 °C; ^1^H-NMR (400 MHz, CDCl_3_) δ 8.62 (s, 1H), 7.47 (d, *J* = 8.8 Hz, 2H), 7.29–7.24 (m, 2H), 7.01 (t, *J* = 7.6 Hz, 1H), 6.91 (d, *J* = 7.6 Hz, 1H), 6.75 (d, *J* = 8.4 Hz, 2H), 5.45 (d, *J* = 8.4 Hz, 1H), 5.14 (s, 1H), 4.16–4.07 (m, 2H), 4.03 (d, *J* = 8.4 Hz, 1H), 3.90–3.84 (m, 1H), 3.81–3.69 (m, 3H), 1.06 (t, *J* = 7.2 Hz, 3H), 0.80 (t, *J* = 7.2 Hz, 3H), 0.76 (t, *J* = 7.2 Hz, 3H); ^13^C-NMR (101 MHz, CDCl_3_) δ 176.21, 171.26, 166.92, 166.85, 147.99, 141.39, 129.90, 126.10, 126.09, 125.22, 124.54 (d, *J*_CF_ = 272.7 Hz), 122.82, 121.67 (d, *J*_CF_ = 33.3 Hz), 115.51, 109.75, 68.63, 64.62, 61.86, 61.61, 61.46, 58.06, 54.78, 13.90, 13.45, 13.40; HRMS: *m*/*z* calcd. for C_27_H_27_F_3_N_2_O_7_ + Na, 571.1668; found, 571.1671.

#### 3.2.2. Synthetic Transformations to Access Other Drug-Like Spirocyclic Scaffolds **3u**–**x**

A mixture of trisubstituted olefins (1.1 mmol), aziridine **2a** (1.0 mmol) and additive TEA (0.5 mmol) in toluene (2 mL) was refluxed at 110 °C under an open atmosphere. The reaction mixture would be cooled to room temperature until most of the 3-ylideneoxindole **1** was consumed (monitored by TLC). Then, the reaction mixture was concentrated and the residue was purified by elaborative chromatography on silica gel to give the final products **3u**–**x** in good yield (up to 78%) with moderate to high diastereoselectivity (up to >20:1). The products were further identified by ^1^H-NMR, ^13^C-NMR and HRMS.

*Diethyl-2-oxo-1′,4′-diphenyl-2H-spiro(acenaphthylene-1,3′-pyrrolidine)-2′,5′-dicarboxylate* (**3u**). The mixed two isomers were isolated by flash chromatography (petroleum ether/ethyl acetate = 5:1) in 76% yield (75.4 mg). The *dr* value was calculated to be 8:1 from crude ^1^H-NMR analysis of the mixture. After which, the pure major isomer **3u** was obtained as a white solid after elaborative chromatography (petroleum ether/ethyl acetate = 10:1) in 68% yield (66.9 mg). m.p. 154–156 °C; ^1^H-NMR (400 MHz, CDCl_3_) δ 7.94–7.91 (m, 2H), 7.76–7.71 (m, 2H), 7.61–7.55 (m, 2H), 7.23 (t, *J* = 8.0 Hz, 2H), 7.00–6.97 (m, 2H), 6.87–6.81 (m, 4H), 6.76 (d, *J* = 8.0 Hz, 2H), 5.53 (d, *J* = 10.4 Hz, 1H), 5.44 (s, 1H), 4.48 (d, *J* = 10.0 Hz, 1H), 4.03–3.93 (m, 2H), 3.46–3.36 (m, 2H), 0.88 (t, *J* = 7.2 Hz, 3H), 0.04 (t, *J* = 7.2 Hz, 3H); ^13^C-NMR (101 MHz, CDCl_3_) δ 201.65, 171.97, 168.09, 145.61, 142.19, 134.83, 132.95, 132.84, 131.87, 130.23, 128.76, 128.22, 128.05, 127.81, 127.76, 127.62, 125.07, 123.99, 121.86, 119.30, 115.42, 68.07, 67.71, 66.11, 61.22, 60.41, 57.32, 13.82, 12.78; HRMS: *m*/*z* calcd. for C_33_H_29_NO_5_ + Na, 542.1943; found, 542.1945.

*Diethyl-1,3-dioxo-1′,4′-diphenyl-1,3-dihydrospiro(indene-2,3′-pyrrolidine)-2′,5′-dicarboxylate* (**3v**). The mixed two isomers were isolated by flash chromatography (petroleum ether/ethyl acetate = 5:1) in 90% yield (85.3 mg). The *dr* value was calculated to be 4:1 from crude ^1^H-NMR analysis of the mixture. After which, the pure major isomer **3v** was obtained as a white solid after elaborative chromatography (petroleum ether/ethyl acetate = 10:1) in 72% yield (68.2 mg). m.p. 133–135 °C; ^1^H-NMR (400 MHz, CDCl_3_) δ 7.94 (d, *J* = 7.6 Hz, 1H), 7.75–7.67 (m, 3H), 7.22–7.15 (m, 4H), 7.10–7.05 (m, 3H), 6.79 (t, *J* = 7.6 Hz, 1H), 6.70 (d, *J* = 7.6 Hz, 2H), 5.57 (d, *J* = 10.0 Hz, 1H), 5.33 (s, 1H), 4.23 (d, *J* = 10.4 Hz, 1H), 4.01–3.92 (m, 2H), 3.84–3.72 (m, 2H), 0.89 (t, *J* = 7.2 Hz, 3H), 0.64 (t, *J* = 7.2 Hz, 3H); ^13^C-NMR (101 MHz, CDCl_3_) δ 198.08, 197.65, 171.57, 168.05, 145.09, 142.18, 142.05, 136.00, 135.74, 131.84, 128.71, 128.64, 128.46, 128.30, 123.27, 123.08, 119.11, 115.14, 66.58, 66.13, 65.76, 61.23, 61.18, 56.29, 13.82, 13.33; HRMS: *m*/*z* calcd. for C_30_H_27_NO_6_ + Na, 520.1736; found, 520.1733.

*Diethyl-1-methyl-4-oxo-3,7,9-triphenyl-2,3,7-triazaspiro(4.4)non-1-ene-6,8-dicarboxylate* (**3w**). The mixed two isomers were isolated by flash chromatography (petroleum ether/ethyl acetate = 5:1) in 93% yield (92.4 mg). The *dr* value was calculated to be 5:1 from crude ^1^H-NMR analysis of the mixture. After which, the pure major isomer **3w** was obtained as a white solid after elaborative chromatography (petroleum ether/ethyl acetate = 10:1) in 78% yield (76.8 mg). m.p. 165–167 °C; ^1^H-NMR (400 MHz, CDCl_3_) δ 7.49 (d, *J* = 8.0 Hz, 2H), 7.32 (d, *J* = 6.8 Hz, 2H), 7.27 (s, 1H), 7.25–7.20 (m, 6H), 7.09 (t, *J* = 7.2 Hz, 1H), 6.82 (t, *J* = 7.2 Hz, 1H), 6.71 (d, *J* = 8.0 Hz, 2H), 5.62 (d, *J* = 9.6 Hz, 1H), 5.12 (s, 1H), 4.08–4.05 (m, 1H), 4.01 (d, *J* = 7.2 Hz, 1H), 3.98–3.90 (m, 3H), 2.47 (s, 3H), 0.94 (q, *J* = 6.8 Hz, 6H); ^13^C-NMR (101 MHz, CDCl_3_) δ 172.16, 169.60, 167.44, 157.03, 144.78, 137.18, 131.10, 128.86, 128.82, 128.64, 128.39, 125.23, 119.48, 118.97, 115.12, 65.83, 64.85, 64.69, 61.71, 61.43, 55.14, 13.88, 13.82, 13.74; HRMS: *m*/*z* calcd. for C_31_H_31_N_3_O_5_ + Na, 548.2161; found, 548.2159.

*Diethyl-3-benzyl-4-oxo-7,9-diphenyl-2-thioxo-1-thia-3,7-diazaspiro(4.4)nonane-6,8-dicarboxylate* (**3x**): The mixed two isomers were isolated by flash chromatography (petroleum ether/ethyl acetate = 5:1) in 72% yield (78.1 mg). The *dr* value was calculated to be >20:1 from crude ^1^H-NMR analysis of the mixture. After which, the pure major isomer **3x** was obtained as a white solid after elaborative chromatography (petroleum ether/ethyl acetate = 10:1) in 69% yield (74.9 mg). m.p. 162–165 °C; ^1^H-NMR (400 MHz, CDCl_3_) δ 7.31–7.27 (m, 5H), 7.25–7.10 (m, 7H), 6.85 (t, *J* = 7.2 Hz, 1H), 6.67 (d, *J* = 7.6 Hz, 2H), 5.54 (s, 1H), 5.09 (d, *J* = 10.0 Hz, 1H), 4.69 (dd, *J* = 41.6, 14.0 Hz, 2H), 4.55 (d, *J* = 10.0 Hz, 1H), 4.07–4.03 (m, 1H), 3.94–3.85 (m, 3H), 0.94 (t, *J* = 7.2 Hz, 3H), 0.84 (t, *J* = 7.2 Hz, 3H); ^13^C-NMR (101 MHz, CDCl_3_) δ 172.68, 170.92, 169.02, 166.92, 144.85, 134.51, 131.38, 128.96, 128.88, 128.82, 128.78, 128.68, 128.66, 128.29, 120.49, 116.10, 69.76, 66.62, 61.87, 61.51, 57.08, 45.52, 43.00, 13.87, 13.74; HRMS: *m*/*z* calcd. for C_31_H_30_N_2_O_5_S_2_ + Na, 597.1494; found, 597.1497.

## 4. Conclusions

In summary, we have developed an efficient single-step reaction between aziridines and 3-ylideneoxindoles to synthesize diverse spirooxindole-pyrrolidines. This is a straightforward technique for constructing fully substituted pyrrolidines bearing phenyl substituents on the nitrogen atom. The reaction also proceeded smoothly with several other synthetically useful activated trisubstituted olefins to afford some pyrrolidine-fused drug-like spirocyclic scaffolds. Further studies on the biological activities of the resulting spiro-architectures are underway in our laboratory.

## Figures and Tables

**Table 1:**
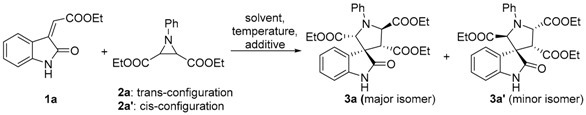
Optimization of the reaction conditions ^a^.

Entry	Solvent	t (h) ^b^	T (°C) ^c^	Additive	Yield (%) ^d^	*dr* ^e^
1	MeCN	4	90	-	40 (30)	3:1
2	EtOH	4	90	-	33 (25)	3:1
3	toluene	4	90	-	45 (36)	4:1
4	1,4-dioxane	4	90	-	35 (26)	3:1
5	DCM	4	90	-	37 (28)	3:1
6	toluene	12	70	-	<10	-
7	toluene	0.5	110	-	55 (37)	2:1
8	toluene	0.5	110	CH_3_COOH	67 (51)	3:1
9	toluene	0.5	110	HCOOH	34 (27)	4:1
10	toluene	0.5	110	ClCH_2_COOH	47 (38)	4:1
11	toluene	0.5	110	CF_3_COOH	n.r.	-
12	toluene	0.5	110	DBU	52 (42)	4:1
13	toluene	0.5	110	DABCO	65 (49)	3:1
14	toluene	0.5	110	K_2_CO_3_	57 (43)	3:1
15	toluene	0.5	110	TEA	78 (65)	5:1
16	toluene	4	90	TEA	72 (58)	4:1
17 ^f^	toluene	0.5	110	TEA	66 (53)	4:1

^a^ Reaction conditions: additive (0.5 mmol) was added to a solution of 3-ylideneoxindole **1a** (1.1 mmol) and aziridine **2a** (1.0 mmol) in solvent (2 mL) at the indicated temperature. ^b^ Time for one-step reaction. ^c^ Temperature of one-step reaction. ^d^ Total yield of the mixed two isomers; the yield of the isolated major isomer **3a** was shown in parentheses. ^e^ Based on crude ^1^H-NMR analysis of the mixed two isomers. ^f^ **2a**′ was used. MeCN = acetonitrile; DCM = dichloromethane; TEA = triethylamine.

**Table 2:**
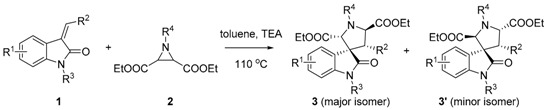
Evaluation of substrate scope under optimized reaction conditions ^a^.

Entry	R^1^	R^2^	R^3^	R^4^	Product	Yield (%) ^b^	*dr* ^c^
1	H	COOEt	H	Ph	**3a**	78 (65)	5:1
2	4-Br	COOEt	H	Ph	**3b**	82 (59)	2.5:1
3	5-F	COOEt	H	Ph	**3c**	83 (67)	4:1
4	5-CH_3_	COOEt	H	Ph	**3d**	81 (69)	6:1
5	H	COO*i*Pr	H	Ph	**3e**	81 (65)	4:1
6	H	COOPh	H	Ph	**3f**	87 (70)	4:1
7	H	(CN)_2_	H	Ph	**3g**	86 (69)	4:1
8	H	Ph	H	Ph	**3h**	63 (61)	>20:1
9	H	4-Br-C_6_H_4_	H	Ph	**3i**	68 (58)	6:1
10	H	2-F-C_6_H_4_	H	Ph	**3j**	66 (63)	>20:1
11	H	4-NO_2_-C_6_H_4_	H	Ph	**3k**	62 (52)	5:1
12	H	4-Me-C_6_H_4_	H	Ph	**3l**	58 (56)	>20:1
13	H	3,4-(MeO)_2_-C_6_H_4_	H	Ph	**3m**	57 (46)	4:1
14	H	2-naphthyl	H	Ph	**3n**	55 (53)	>20:1
15	H	2-furyl	H	Ph	**3o**	46 (35)	3:1
16	H	2-thienyl	H	Ph	**3p**	52 (39)	3:1
17	H	COOEt	Bn	Ph	**3q**	88 (77)	7:1
18	H	COOEt	Boc	Ph	**3r**	82 (71)	6:1
19	H	COOEt	H	3-Me-C_6_H_4_	**3s**	70 (58)	5:1
20	H	COOEt	H	4-CF_3_-C_6_H_4_	**3t**	67 (56)	5:1

^a^ See entry 15 and footnote “a” in Table 1. ^b^ Total yield of the mixed two isomers; the yield of the isolated major isomer **3** was shown in parentheses. ^c^ Based on crude ^1^H-NMR analysis of the mixed two isomers.

## Supplementary Materials

Supplementary Materials can be accessed at: http://www.mdpi.com/1420-3049/21/9/1113/s1.

## Abbreviations

DBU	1,8-Diazabicyclo(5.4.0)undec-7-ene
DABCO	1,4-Diazabicyclo(2.2.2)octane
TEA	Triethylamine
