# Supplementary material for: Efficient Synthesis of Fully Substituted Pyrrolidine-Fused 3-Spirooxindoles via 1,3-Dipolar Cycloaddition of Aziridine and 3-Ylideneoxindole
Source: Molecules. 2016 Aug 24;21(9):1113. doi: 10.3390/molecules21091113 (PMC6274301; doi:10.3390/molecules21091113)
Supplement: Supplementary file 1 [file molecules-21-01113-s001.pdf]

Supplementary Materials: Efficient Synthesis of Fully Efficient Synthesis of Fully Substituted Pyrrolidine-Fused 3-Spirooxindoles via 1,3-Dipolar Cycloaddition of Aziridine and 3-Ylideneoxindole

Wen Ren, Qian Zhao, Chuan Zheng, Qiong Zhao, Li Guo and Wei Huang

1. General Information

NMR data was obtained for ^{1}H at 400 MHz, and for ^{13}C at 101 MHz. Chemical shifts were reported in ppm from tetramethylsilane using solvent resonance in $CDCl_3$ solution as the internal standard. ESI HRMS was performed on a Waters SYNAPT G2. Column chromatography was performed on silica gel (200–300 mesh) using an eluent of ethyl acetate and petroleum ether. TLC was performed on glass-backed silica plates; products were visualized using UV light and I_2. Melting points were determined on a Mel-Temp apparatus and were not corrected. All chemicals were used from Adamas-beta without purification unless otherwise noted.

Compounds **1** were prepared according to the literature [1]. Compound **2** were prepared according to the literature [2].

2. General Producer for the Spirooxindole-Pyrrolidines 3

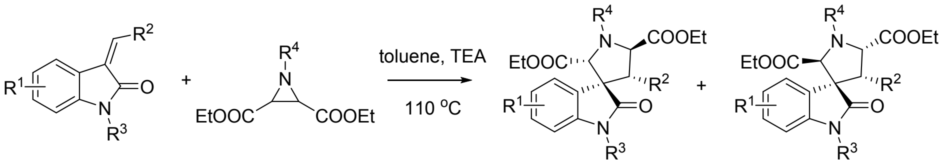

1 **2** **3** (major isomer) **3'** (minor isomer)

A mixture of 3-ylideneoxindole **1** (1.1 mmol), aziridine **2** (1.0 mmol) and additive TEA (0.5 mmol) in toluene (2 mL) was refluxed at 110 °C under an open atmosphere. The reaction mixture was stirred for a specified reaction time until the reaction was completed (monitored by TLC). Then the reaction mixture was concentrated and the residue was isolated by elaborative chromatography on silica gel to give the final product **3**.

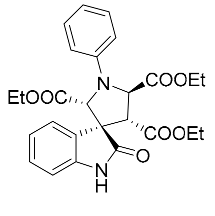

The mixed two isomers were isolated by flash chromatography (petroleum ether/ethyl acetate = 5:1) in 78% yield (71.3 mg). The *dr* value was calculated to be 5:1 from crude ^{1}H-NMR analysis of the mixture. After which, the pure major isomer **3a** was obtained as a white solid after elaborative chromatography (petroleum ether/ethyl acetate = 10:1) in 65% yield (59.4 mg). m.p. 130–132 °C; ^{1}H-NMR (400 MHz, $CDCl_3$) δ 8.04 (s, 1H), 7.35 (d, *J* = 7.2 Hz, 1H), 7.25–7.18 (m, 3H), 6.99 (t, *J* = 7.6 Hz, 1H), 6.85 (dd, *J* = 13.2, 7.6 Hz, 2H), 6.74 (d, *J* = 8.0 Hz, 2H), 5.42 (d, *J* = 8.4 Hz, 1H), 5.11 (s, 1H), 4.09–3.99 (m, 3H), 3.88–3.82 (m, 1H), 3.80–3.66 (m, 3H), 0.99 (t, *J* = 7.2 Hz, 3H), 0.79 (t, *J* = 7.2 Hz, 3H), 0.75 (t, *J* = 7.2 Hz, 3H); ^{13}C-NMR (101 MHz, $CDCl_3$) δ 176.14, 171.80, 167.35, 167.32, 145.26, 141.23, 129.59, 128.73, 126.28, 125.67, 122.71, 120.26, 116.39, 109.40, 68.76, 64.88, 61.45, 61.41, 61.08, 58.06, 54.64, 13.85, 13.49, 13.42; HRMS: *m*/*z* calcd. for $C_{26}H_{28}N_2O_7$+Na, 503.1794; found, 503.1790.

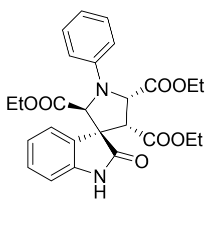

The pure minor isomer **3a′** was obtained as a semi-solid after elaborative chromatography (petroleum ether/ethyl acetate = 10:1) in 13% yield (11.9 mg). ^{1}H-NMR (400 MHz, $CDCl_3$) δ 8.43 (s, 1H), 8.24 (d, *J* = 7.6 Hz, 1H), 7.24–7.20 (m, 3H), 7.05 (t, *J* = 7.6 Hz, 1H), 6.90–6.83 (m, 2H), 6.67 (d, *J* = 8.0 Hz, 2H), 4.99 (d, *J* = 10.8 Hz, 1H), 4.72 (s, 1H), 4.53 (d, *J* = 10.8 Hz, 1H), 4.47–4.39 (m, 1H), 4.29–4.21 (m, 1H), 4.11–4.08 (m, 2H), 3.88–3.80 (m, 1H), 3.75–3.67 (m, 1H), 1.37 (t, *J* = 7.2 Hz, 3H), 1.13 (t, *J* = 7.2 Hz, 3H), 0.76 (t, *J* = 7.2 Hz, 3H); ^{13}C-NMR (101 MHz, $CDCl_3$) δ 175.69, 171.24, 169.74,

167.24, 145.33, 140.19, 130.41, 129.43, 129.08, 126.32, 123.47, 119.93, 114.75, 109.29, 71.19, 62.15, 61.56, 61.42, 61.14, 60.43, 52.95, 14.03, 13.99, 13.37; HRMS: *m*/*z* calcd. for $C_{26}H_{28}N_2O_7$ + Na, 503.1794; found, 503.1798.

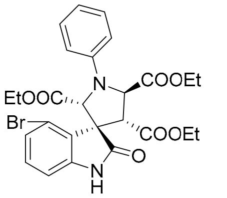

The mixed two isomers were isolated by flash chromatography (petroleum ether/ethyl acetate = 5:1) in 82% yield (86.7 mg). The *dr* value was calculated to be 2.5:1 from crude ^{1}H-NMR analysis of the mixture. The pure major isomer **3b** could not be separated in pure form after elaborative chromatography; the yield of **3b** was calculated to be 59% based on the total yield and *dr* value. m.p. 128–130 °C; ^{1}H-NMR (400 MHz, $CDCl_3$) δ 8.74 (s, 1H), 7.24–7.14 (m, 4H), 6.87–6.82 (m, 2H), 6.72 (d, *J* = 8.0 Hz, 2H), 5.49 (s, 1H), 5.40 (d, *J* = 8.8 Hz, 1H), 4.81 (d, *J* = 8.8 Hz, 1H), 4.18–4.08 (m, 4H), 4.06–4.02 (m, 2H), 1.14 (t, *J* = 7.2 Hz, 3H), 1.11 (t, *J* = 7.2 Hz, 3H), 0.89 (t, *J* = 7.2Hz, 3H); ^{13}C-NMR (101 MHz, $CDCl_3$) δ 176.33, 172.46, 168.35, 168.22, 144.79, 144.09, 130.81, 128.81, 128.67, 127.02, 119.60, 118.46, 116.37, 115.42, 109.43, 64.93, 64.31, 61.69, 61.40, 58.94, 51.01, 14.01, 13.68, 13.62; HRMS: *m*/*z* calcd. for $C_{26}H_{27}BrN_2O_7$ + Na, 581.0899; found, 581.0901.

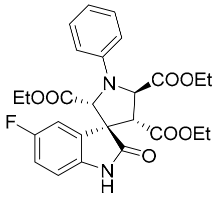

The mixed two isomers were isolated by flash chromatography (petroleum ether/ethyl acetate = 5:1) in 83% yield (78.2 mg). The *dr* value was calculated to be 4:1 from crude ^{1}H-NMR analysis of the mixture. After which, the pure major isomer **3c** was obtained as a white solid after elaborative chromatography (petroleum ether/ethyl acetate = 10:1) in 67% yield (62.6 mg). m.p. 120–122 °C; ^{1}H-NMR (400 MHz, $CDCl_3$) δ 8.90 (s, 1H), 7.22 (t, *J* = 8.0 Hz, 2H), 7.13 (dd, *J* = 8.0, 2.4 Hz, 1H), 6.99–6.92 (m, 1H), 6.89–6.84 (m, 2H), 6.76 (d, *J* = 8.0 Hz, 2H), 5.38 (d, *J* = 8.0 Hz, 1H), 5.11 (s, 1H), 4.09–4.01 (m, 3H), 3.94–3.72 (m, 4H), 1.01 (t, *J* = 7.2 Hz, 3H), 0.85 (t, *J* = 7.2 Hz, 3H), 0.78 (t, *J* = 7.2 Hz, 3H); ^{13}C-NMR (101 MHz, $CDCl_3$) δ 176.48, 171.68, 167.25, 167.13, 158.64 (d, J_{CF} = 243.4 Hz), 145.09, 137.56 (d, J_{CF} = 2.0 Hz), 128.78, 127.28 (d, J_{CF} = 8.1 Hz), 120.59, 116.60, 116.12 (d, J_{CF} = 23.2 Hz), 114.21 (d, J_{CF} = 25.3 Hz), 110.26 (d, J_{CF} = 8.1 Hz), 68.70, 64.70, 61.59, 61.54, 61.21, 58.60, 54.52, 13.86, 13.50, 13.48; HRMS: *m*/*z* calcd. for $C_{26}H_{27}FN_2O_7$ + Na, 521.1700; found, 521.1696.

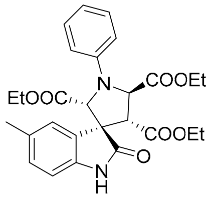

The mixed two isomers were isolated by flash chromatography (petroleum ether/ethyl acetate = 5:1) in 81% yield (75.7 mg). The *dr* value was calculated to be 6:1 from crude ^{1}H-NMR analysis of the mixture. After which, the pure major isomer **3d** was obtained as a white solid after elaborative chromatography (petroleum ether/ethyl acetate = 10:1) in 69% yield (64.9 mg). m.p. 120–123 °C; ^{1}H-NMR (400 MHz, $CDCl_3$) δ 8.61 (s, 1H), 7.21 (dd, *J* = 8.4, 7.6 Hz, 2H), 7.15 (s, 1H), 7.02 (dd, *J* = 8.0, 0.8 Hz, 1H), 6.84 (t, *J* = 7.2 Hz, 1H), 6.79–6.74 (m, 3H), 5.42 (d, *J* = 8.8 Hz, 1H), 5.11 (s, 1H), 4.10–4.01 (m, 3H), 3.90–3.82 (m, 1H), 3.78–3.74 (m, 1H), 3.72–3.69 (m, 2H), 2.27 (s, 3H), 1.00 (t, *J* = 7.2 Hz, 3H), 0.80 (t, *J* = 7.2 Hz, 3H), 0.75 (t, *J* = 7.2 Hz, 3H); ^{13}C-NMR (101 MHz, $CDCl_3$) δ 176.60, 171.87, 167.38, 167.34, 145.31, 138.99, 132.18, 129.90, 128.74, 126.73, 125.68, 120.09, 116.19, 109.32, 68.71, 64.90, 61.44, 61.37, 61.02, 58.23, 54.68, 21.09, 13.86, 13.48, 13.40; HRMS: *m*/*z* calcd. for $C_{27}H_{30}N_2O_7$ + Na, 517.1951; found, 517.1954.

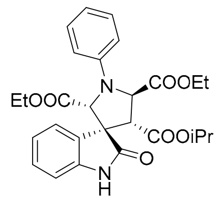

The mixed two isomers were isolated by flash chromatography (petroleum ether/ethyl acetate = 5:1) in 81% yield (76.3 mg). The *dr* value was calculated to be 4:1 from crude ^{1}H-NMR analysis of the mixture. After which, the pure major isomer **3e** was obtained as a white solid after elaborative chromatography (petroleum ether/ethyl acetate = 10:1) in 65% yield (61.2 mg). m.p. 110–115 °C; ^{1}H-NMR (400 MHz, $CDCl_3$) δ 8.27 (d, *J* = 7.2 Hz, 1H), 7.92 (s, 1H), 7.25–7.20 (m, 3H), 7.05 (td, *J* = 7.6, 0.8 Hz, 1H), 6.89–6.83 (m, 2H), 6.67 (d, *J* = 8.0 Hz, 2H), 4.97 (d, *J* = 10.8 Hz, 1H), 4.70 (s, 1H), 4.66–4.58 (m, 1H), 4.50 (d, *J* = 10.8 Hz, 1H), 4.48–4.40 (m, 1H), 4.30–4.21 (m, 1H), 4.16–4.04 (m, 2H), 1.37 (t, *J* = 7.2 Hz, 3H), 1.13 (t, *J* = 7.2 Hz, 3H), 1.03 (d, *J* = 6.4 Hz, 3H), 0.56 (d, *J* = 6.4 Hz, 3H).^{13}C-NMR (101 MHz, $CDCl_3$) δ 175.43, 171.37, 169.77, 166.74, 145.38, 140.19, 130.54, 129.42, 129.03, 126.41, 123.49, 119.91, 114.74, 109.18, 71.32, 69.04, 62.16, 61.54, 61.42, 57.63, 52.95, 21.42, 20.56, 14.04, 13.99; HRMS: *m*/*z* calcd. for $C_{27}H_{30}N_2O_7$ + Na, 517.1951; found, 517.1948.

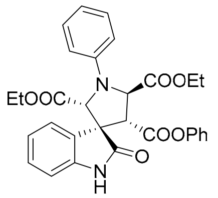

The mixed two isomers were isolated by flash chromatography (petroleum ether/ethyl acetate = 5:1) in 87% yield (86.9 mg). The *dr* value was calculated to be 4:1 from crude ^{1}H-NMR analysis of the mixture. After which, the pure major isomer **3f** was obtained as a white solid after elaborative chromatography (petroleum ether/ethyl acetate = 10:1) in 70% yield (69.5 mg). m.p. 135–137 °C; ^{1}H-NMR (400 MHz, $CDCl_3$) δ 8.68 (s, 1H), 7.46 (d, *J* = 7.2 Hz, 1H), 7.30 (td, *J* = 7.6, 0.8 Hz, 1H), 7.23–7.14 (m, 4H), 7.13–7.05 (m, 2H), 6.91–6.84 (m, 2H), 6.78 (d, *J* = 8.0 Hz, 2H), 6.28–6.25 (m, 2H), 5.51 (d, *J* = 8.0 Hz, 1H), 5.19 (s, 1H), 4.30 (d, *J* = 8.0 Hz, 1H), 4.11–4.03 (m, 2H), 3.75–3.65 (m, 2H), 1.01 (t, *J* = 7.2 Hz, 3H), 0.74 (t, *J* = 7.2 Hz, 3H); ^{13}C-NMR (101 MHz, $CDCl_3$) δ 176.29, 171.74, 167.25, 166.36, 149.72, 145.16, 141.62, 129.88, 129.33, 128.80, 126.46, 126.20, 125.62, 122.97, 120.96, 120.50, 116.59, 109.99, 68.88, 64.87, 61.60, 61.20, 58.22, 54.62, 13.88, 13.50; HRMS: *m*/*z* calcd. for $C_{30}H_{28}N_2O_7$ + Na, 551.1794; found, 551.1798.

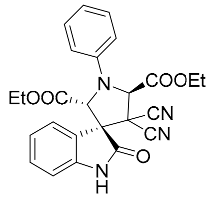

The mixed two isomers were isolated by flash chromatography (petroleum ether/ethyl acetate = 5:1) in 86% yield (75.3 mg). The *dr* value was calculated to be 4:1 from crude ^{1}H-NMR analysis of the mixture. After which, the pure major isomer **3g** was obtained as a white solid after elaborative chromatography (petroleum ether/ethyl acetate = 10:1) in 69% yield (60.2 mg). m.p. 140–142 °C; ^{1}H-NMR (400 MHz, $CDCl_3$) δ 8.72 (s, 1H), 7.46 (d, *J* = 7.6 Hz, 1H), 7.38 (t, *J* = 7.6 Hz, 1H), 7.29–7.25 (m, 2H), 7.13 (t, *J* = 7.6 Hz, 1H), 7.02 (d, *J* = 7.6 Hz, 1H), 6.95 (t, *J* = 7.6 Hz, 1H), 6.74 (t, *J* = 8.0 Hz, 2H), 5.44 (s, 1H), 5.23 (s, 1H), 4.36–4.24 (m, 2H), 3.83 (q, *J* = 7.2 Hz, 2H), 1.25–1.21(m, 3H), 0.78 (t, *J* = 7.2 Hz, 3H); ^{13}C-NMR (101 MHz, $CDCl_3$) δ 172.46, 166.81, 165.21, 143.61, 141.50, 131.86, 129.18, 126.81, 123.48, 121.60, 121.55, 116.69, 111.71, 111.27, 109.85, 69.27, 66.25, 63.14, 61.89, 59.31, 45.06, 13.82, 13.46; HRMS: *m*/*z* calcd. for $C_{25}H_{22}N_4O_5$ + Na, 481.1488; found, 481.1489.

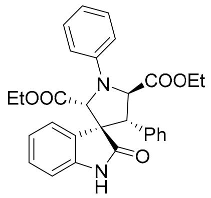

The mixed two isomers were isolated by flash chromatography (petroleum ether/ethyl acetate = 5:1) in 63% yield (57.6 mg). The *dr* value was calculated to be >20:1 from crude ^{1}H-NMR analysis of the mixture. After which, the pure major isomer **3h** was obtained as a white solid after elaborative chromatography (petroleum ether/ethyl acetate = 10:1) in 61% yield (55.8 mg). m.p. 130–132 °C; ^{1}H-NMR (400 MHz, $CDCl_3$) δ 8.34 (s, 1H), 7.48 (d, *J* = 7.2 Hz, 1H), 7.21 (dd, *J* = 8.4, 7.6 Hz, 2H), 7.12–7.04 (m, 6H), 7.01–6.97 (m, 1H), 6.81 (t, *J* = 7.2 Hz, 1H), 6.73 (d, *J* = 8.0 Hz, 2H), 6.63 (d, *J* = 7.6 Hz, 1H), 5.39 (d, *J* = 10.4 Hz, 1H), 5.33 (s, 1H), 4.30 (d, *J* = 10.4 Hz, 1H), 4.00–3.89 (m, 2H), 3.80–3.65 (m, 2H), 0.86 (t, *J* = 7.2 Hz, 3H), 0.68 (t, *J* = 7.2 Hz, 3H); ^{13}C-NMR (101 MHz, $CDCl_3$) δ 176.48, 171.79, 167.85, 145.42, 140.58, 132.31, 129.10, 128.77, 128.34, 128.04, 128.01, 126.86, 125.52, 122.24, 119.40, 115.35, 109.64, 67.54, 67.20, 61.68, 61.26, 60.93, 57.21, 13.79, 13.46; HRMS: *m*/*z* calcd. for $C_{29}H_{28}N_2O_5$ + Na, 507.1896; found, 507.1900.

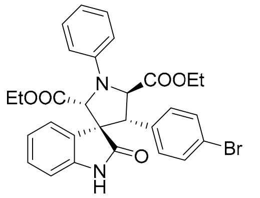

The mixed two isomers were isolated by flash chromatography (petroleum ether/ethyl acetate = 5:1) in 68% yield (73.2 mg). The *dr* value was calculated to be 6:1 from crude ^{1}H-NMR analysis of the mixture. After which, the pure major isomer **3i** was obtained as a white solid after elaborative chromatography (petroleum ether/ethyl acetate = 10:1) in 58% yield (62.7 mg). m.p. 79–82 °C; ^{1}H-NMR (400 MHz, $CDCl_3$) δ 8.20 (s, 1H), 7.47 (d, *J* = 7.6 Hz, 1H), 7.24–7.20 (m, 4H), 7.14 (t, *J* = 7.6 Hz, 1H), 7.02–6.94 (m, 3H), 6.83 (t, *J* = 7.2 Hz, 1H), 6.71 (d, *J* = 8.0 Hz, 2H), 6.66 (d, *J* = 7.6 Hz, 1H), 5.33–5.31 (m, 2H), 4.24 (d, *J* = 10.4 Hz, 1H), 3.99–3.93 (m, 2H), 3.80–3.65 (m, 2H), 0.88 (t, *J* = 7.2 Hz, 3H), 0.68 (t, *J* = 7.2 Hz, 3H); ^{13}C-NMR (101 MHz, $CDCl_3$) δ 176.26, 171.62, 167.74, 145.25, 140.61, 131.41, 131.27, 130.02, 129.37, 128.81, 126.72, 125.12, 122.38, 122.24, 119.55, 115.34, 109.95, 67.49, 67.16, 61.46, 61.42, 61.02, 56.59, 13.82, 13.45; HRMS: *m*/*z* calcd. for $C_{29}H_{27}BrN_2O_5$ + Na, 585.1001; found, 585.1003.

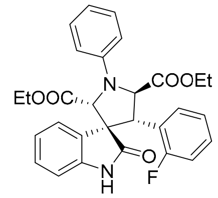

The mixed two isomers were isolated by flash chromatography (petroleum ether/ethyl acetate = 5:1) in 66% yield (63.1 mg). The *dr* value was calculated to be >20:1 from crude ^{1}H-NMR analysis of the mixture. After which, the pure major isomer **3j** was obtained as a white solid after elaborative chromatography (petroleum ether/ethyl acetate = 10:1) in 63% yield (60.2 mg). m.p. 137–140 °C; ^{1}H-NMR (400 MHz, $CDCl_3$) δ 8.46 (s, 1H), 7.39 (d, *J* = 7.6 Hz, 1H), 7.22 (t, *J* = 8.0 Hz, 2H), 7.16–7.11 (m, 2H), 7.09–7.06 (m, 1H), 6.95 (t, *J* = 7.6 Hz, 1H), 6.88–6.80 (m, 3H), 6.73 (d, *J* = 8.4 Hz, 2H), 6.69 (d, *J* = 8.0 Hz, 1H), 5.41 (d, *J* = 9.6 Hz, 1H), 5.34 (s, 1H), 4.66 (d, *J* = 9.6 Hz, 1H), 4.04–3.92 (m, 2H), 3.79–3.66 (m, 2H), 0.88 (t, *J* = 7.2 Hz, 3H), 0.68 (t, *J* = 7.2 Hz, 3H); ^{13}C-NMR (101 MHz, $CDCl_3$) δ 176.82, 171.65, 167.92, 161.00 (d, J_{CF} = 249.5 Hz), 145.44, 141.01, 129.78 (d, J_{CF} = 3.0 Hz), 129.55 (d, J_{CF} = 9.1 Hz), 129.21, 128.81, 126.98, 125.44, 123.60 (d, J_{CF} = 3.0 Hz), 122.04, 120.17 (d, J_{CF} = 14.1 Hz), 119.51, 115.46, 115.39 (d, J_{CF} = 23.2 Hz), 109.71, 67.75, 67.11, 61.32, 60.98, 60.78, 49.67, 13.75, 13.43; HRMS: *m*/*z* calcd. for $C_{29}H_{27}FN_2O_5$ + Na, 525.1802; found, 525.1804.

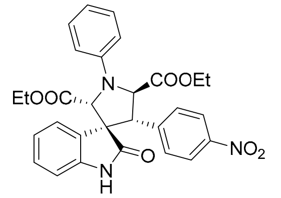

The mixed two isomers were isolated by flash chromatography (petroleum ether/ethyl acetate = 5:1) in 62% yield (62.8 mg). The *dr* value was calculated to be 5:1 from crude ^{1}H-NMR analysis of the mixture. After which, the pure major isomer **3k** was obtained as a white solid after elaborative chromatography (petroleum ether/ethyl acetate = 10:1) in 52% yield (52.3 mg). m.p. 90–93 °C; ^{1}H-NMR (400 MHz, $CDCl_3$) δ 7.95 (d, *J* = 8.8 Hz, 2H), 7.94–7.89 (m, 1H), 7.48 (d, *J* = 7.6 Hz, 1H), 7.29 (d, *J* = 8.8 Hz, 2H), 7.24 (d, *J* = 8.0 Hz, 2H), 7.14 (t, *J* = 7.6 Hz, 1H), 7.02 (t, *J* = 7.6 Hz, 1H), 6.85 (t, *J* = 7.2 Hz, 1H), 6.73 (d, *J* = 8.0 Hz, 2H), 6.65 (d, *J* = 7.6 Hz, 1H), 5.41 (d, *J* = 10.0 Hz, 1H), 5.34 (s, 1H), 4.38 (d, *J* = 10.4 Hz, 1H), 4.02–3.92 (m, 2H), 3.82–3.67 (m, 2H), 0.88 (t, *J* = 7.2 Hz, 3H), 0.69 (t, *J* = 7.2 Hz, 3H); ^{13}C-NMR (101 MHz, $CDCl_3$) δ 175.72, 171.33, 167.55, 147.58, 145.05, 140.37, 140.00, 129.69, 129.31, 128.88, 126.70, 124.65, 123.26, 122.61, 119.84, 115.42, 109.97, 67.58, 66.90, 61.63, 61.41, 61.13, 56.49, 13.81, 13.46; HRMS: *m*/*z* calcd. for $C_{29}H_{27}N_3O_7$ + Na, 552.1747; found, 552.1744.

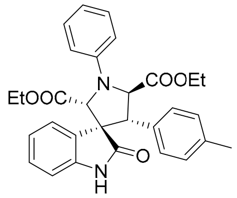

The mixed two isomers were isolated by flash chromatography (petroleum ether/ethyl acetate = 5:1) in 58% yield (55.3 mg). The *dr* value was calculated to be >20:1 from crude ^{1}H-NMR analysis of the mixture. After which, the pure major isomer **3l** was obtained as a white solid after elaborative chromatography (petroleum ether/ethyl acetate = 10:1) in 56% yield (53.4 mg). m.p. 85–88 °C; ^{1}H-NMR (400 MHz, $CDCl_3$) δ 8.14 (s, 1H), 7.49 (d, *J* = 7.6 Hz, 1H), 7.21 (t, *J* = 7.6 Hz, 2H), 7.11 (t, *J* = 7.6 Hz, 1H), 7.00 (t, *J* = 7.6 Hz, 1H), 6.95–6.80 (m, 5H), 6.73 (d, *J* = 8.4 Hz, 2H), 6.63 (d, *J* = 8.0 Hz, 1H), 5.35 (d, *J* = 10.4 Hz, 1H), 5.31 (s, 1H), 4.25 (d, *J* = 10.0 Hz, 1H), 4.00–3.89 (m, 2H), 3.80–3.65 (m, 2H), 2.13 (s, 3H), 0.86 (t, *J* = 7.2 Hz, 3H), 0.68 (t, *J* = 7.2 Hz, 3H); ^{13}C-NMR (101 MHz, $CDCl_3$) δ 176.55, 171.87, 167.89, 145.47, 140.63, 137.59, 132.16, 129.25, 129.06, 128.76, 127.83, 126.92, 125.33, 122.14, 119.38, 115.37, 109.69, 67.54, 67.44, 61.68, 61.23, 60.92, 57.20, 21.24, 13.79, 13.46; HRMS: *m*/*z* calcd. for $C_{30}H_{30}N_2O_5$ + Na, 521.2052; found, 521.2056.

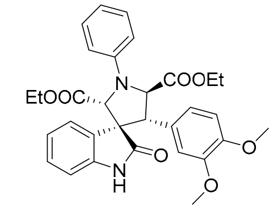

The mixed two isomers were isolated by flash chromatography (petroleum ether/ethyl acetate = 5:1) in 57% yield (59.4 mg). The *dr* value was calculated to be 4:1 from crude ^{1}H-NMR analysis of the mixture. After which, the pure major isomer **3m** was obtained as a white solid after elaborative chromatography (petroleum ether/ethyl acetate = 10:1) in 46% yield (47.5 mg). m.p. 100–103 °C; ^{1}H-NMR (400 MHz, $CDCl_3$) δ 7.52 (d, *J* = 7.2 Hz, 1H), 7.28 (s, 1H), 7.22 (dd, *J* = 8.0, 7.6 Hz, 2H), 7.14 (t, *J* = 7.6 Hz, 1H), 7.02 (t, *J* = 7.6 Hz, 1H), 6.82 (t, *J* = 7.6 Hz, 1H), 6.72 (d, *J* = 8.0 Hz, 2H), 6.66–6.63 (m, 2H), 6.57 (d, *J* = 8.0 Hz, 1H), 6.46 (d, *J* = 2.0 Hz, 1H), 5.30 (s, 1H), 5.24 (d, *J* = 10.4 Hz, 1H), 4.22 (d, *J* = 10.4 Hz, 1H), 4.02–3.91 (m, 2H), 3.83–3.79 (m, 1H), 3.75 (s, 3H), 3.71–3.67 (m, 1H), 3.61 (s, 3H), 0.88 (t, *J* = 7.2 Hz, 3H), 0.69 (t, *J* = 7.2 Hz, 3H); ^{13}C-NMR (101 MHz, $CDCl_3$) δ 175.17, 172.71, 168.64, 148.59, 148.03, 145.23, 141.06, 129.07, 128.81, 128.74, 124.30, 123.10,

122.96, 120.62, 118.92, 114.75, 111.36, 110.49, 109.64, 68.45, 65.15, 61.66, 61.29, 61.13, 58.39, 55.60, 55.49, 13.98, 13.64; HRMS: *m*/*z* calcd. for $C_{31}H_{32}N_2O_7$ + Na, 567.2107; found, 567.2110.

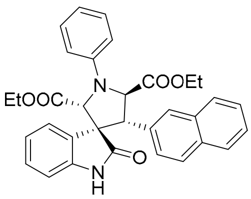

The mixed two isomers were isolated by flash chromatography (petroleum ether/ethyl acetate = 5:1) in 55% yield (55.7 mg). The *dr* value was calculated to be >20:1 from crude ^{1}H-NMR analysis of the mixture. After which, the pure major isomer **3n** was obtained as a white solid after elaborative chromatography (petroleum ether/ethyl acetate = 10:1) in 53% yield (53.5 mg). m.p. 105–107 °C; ^{1}H-NMR (400 MHz, $CDCl_3$) δ 8.02 (s, 1H), 7.67–7.65 (m, 2H), 7.59–7.51 (m, 3H), 7.41–7.37 (m, 2H), 7.24–7.14 (m, 3H), 7.07–6.99 (m, 2H), 6.82 (t, *J* = 7.2 Hz, 1H), 6.75 (d, *J* = 8.0 Hz, 2H), 6.53 (d, *J* = 7.2 Hz, 1H), 5.50 (d, *J* = 10.4 Hz, 1H), 5.37 (s, 1H), 4.47 (d, *J* = 10.0 Hz, 1H), 3.96–3.88 (m, 2H), 3.81–3.62 (m, 2H), 0.83 (t, *J* = 7.2 Hz, 3H), 0.66 (t, *J* = 7.2 Hz, 3H); ^{13}C-NMR (101 MHz, $CDCl_3$) δ 176.39, 171.83, 167.84, 145.44, 140.61, 132.89, 132.87, 129.93, 129.18, 128.79, 128.14, 127.96, 127.62, 127.44, 126.91, 126.12, 126.02, 125.71, 125.47, 122.25, 119.46, 115.41, 109.78, 67.62, 67.48, 61.75, 61.31, 60.96, 57.42, 13.80, 13.46; HRMS: *m*/*z* calcd. for $C_{33}H_{30}N_2O_5$ + Na, 557.2052; found, 557.2049.

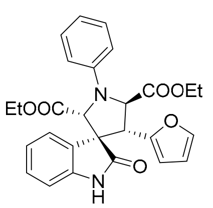

The mixed two isomers were isolated by flash chromatography (petroleum ether/ethyl acetate = 5:1) in 46% yield (41.3 mg). The *dr* value was calculated to be 3:1 from crude ^{1}H-NMR analysis of the mixture. After which, the pure major isomer **3o** was obtained as a white solid after elaborative chromatography (petroleum ether/ethyl acetate = 10:1) in 35% yield (31.1 mg). m.p. 140–143 °C; ^{1}H-NMR (400 MHz, $CDCl_3$) δ 8.41 (s, 1H), 7.34 (d, *J* = 7.6 Hz, 1H), 7.22 (t, *J* = 8.0 Hz, 2H), 7.15 (t, *J* = 7.6 Hz, 1H), 7.05 (s, 1H), 6.95 (t, *J* = 7.6 Hz, 1H), 6.85–6.77 (m, 2H), 6.72 (d, *J* = 8.0 Hz, 2H), 6.07–6.01 (m, 2H), 5.27 (d, *J* = 10.0 Hz, 1H), 5.25 (s, 1H), 4.43 (d, *J* = 9.6 Hz, 1H), 4.11–3.94 (m, 2H), 3.79–3.66 (m, 2H), 0.94 (t, *J* = 7.2 Hz, 3H), 0.71 (t, *J* = 7.2 Hz, 3H); ^{13}C-NMR (101 MHz, $CDCl_3$) δ 176.58, 171.71, 167.64, 147.90, 145.28, 142.35, 140.79, 129.15, 128.80, 126.74, 125.63, 122.31, 119.66, 115.53, 110.03, 109.57, 107.99, 67.67, 66.83, 61.47, 61.02, 60.03, 50.42, 13.81, 13.48; HRMS: *m*/*z* calcd. for $C_{27}H_{26}N_2O_6$ + Na, 497.1689; found, 497.1687.

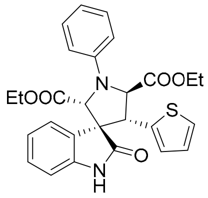

The mixed two isomers were isolated by flash chromatography (petroleum ether/ethyl acetate = 5:1) in 52% yield (48.1 mg). The *dr* value was calculated to be 3:1 from crude ^{1}H-NMR analysis of the mixture. After which, the pure major isomer **3p** was obtained as a white solid after elaborative chromatography (petroleum ether/ethyl acetate = 10:1) in 39% yield (36.2 mg). m.p. 192–194 °C; ^{1}H-NMR (400 MHz, $CDCl_3$) δ 8.14 (s, 1H), 7.48 (d, *J* = 7.6 Hz, 1H), 7.23–7.18 (m, 3H), 7.06–7.01 (m, 2H), 6.84–6.70 (m, 6H), 5.29 (s, 1H), 5.17 (d, *J* = 10.4 Hz, 1H), 4.59 (d, *J* = 10.0 Hz, 1H), 4.05–3.93 (m, 2H), 3.82–3.67 (m, 2H), 0.90 (t, *J* = 7.2 Hz, 3H), 0.70 (t, *J* = 7.2 Hz, 3H); ^{13}C-NMR (101 MHz, $CDCl_3$) δ 176.14, 171.42, 167.67, 145.21, 141.10, 134.68, 129.51, 128.77, 127.24, 127.05, 126.19, 125.70, 125.39, 122.54, 119.60, 115.44, 109.82, 69.71, 67.23, 61.41, 61.21, 61.01, 53.06, 13.82, 13.47; HRMS: *m*/*z* calcd. for $C_{27}H_{26}N_2O_5S$ + Na, 513.1460; found, 513.1458.

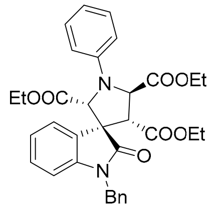

The mixed two isomers were isolated by flash chromatography (petroleum ether/ethyl acetate = 5:1) in 88% yield (95.5 mg). The *dr* value was calculated to be 7:1 from crude ^{1}H-NMR analysis of the mixture. After which, the pure major isomer **3q** was obtained as a white solid after elaborative chromatography (petroleum ether/ethyl acetate = 10:1) in 77% yield (83.6 mg). m.p. 170–172 °C; ^{1}H-NMR (400 MHz, $CDCl_3$) δ 7.48 (d, *J* = 8.0 Hz, 2H), 7.36–7.34 (m, 3H), 7.31–7.29 (m, 1H), 7.24–7.16 (m, 3H), 6.96 (t, *J* = 7.6 Hz, 1H), 6.84 (t, *J* = 7.2 Hz, 1H), 6.76 (t, *J* = 8.4 Hz 3H), 5.44 (dd, *J* = 8.0, 1.0 Hz, 1H), 5.18 (s, 1H), 4.98 (d, *J* = 5.2 Hz, 2H), 4.10 (d, *J* = 8.4 Hz, 1H), 4.06–4.02 (m, 2H), 3.79–3.75 (m, 1H), 3.64–3.55 (m, 3H), 1.01–0.98 (m, 3H), 0.56–0.50 (m, 6H); ^{13}C-NMR (101 MHz, $CDCl_3$) δ 174.83, 171.78, 167.32, 167.28, 145.35, 143.43, 135.68, 129.38, 128.72, 128.68, 128.08, 127.92, 125.95, 125.38, 122.68, 120.20, 116.41, 108.67, 68.94, 65.08, 61.40, 60.99, 57.50, 54.78, 44.65, 22.66, 13.86, 13.30, 13.24; HRMS: *m*/*z* calcd. for $C_{33}H_{34}N_2O_7$ + Na, 593.2264; found, 593.2266.

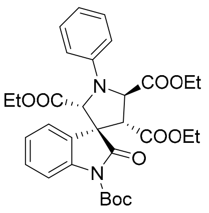

The mixed two isomers were isolated by flash chromatography (petroleum ether/ethyl acetate = 5:1) in 82% yield (90.3 mg). The *dr* value was calculated to be 6:1 from crude ^{1}H-NMR analysis of the mixture. After which, the pure major isomer **3r** was obtained as a white solid after elaborative chromatography (petroleum ether/ethyl acetate = 10:1) in 71% yield (77.4 mg). m.p. 150–153 °C; ^{1}H-NMR (400 MHz, CDCl$_3$) δ 7.86 (d, *J* = 8.4 Hz, 1H), 7.40 (dd, *J* = 7.6, 0.8 Hz, 1H), 7.35–7.30 (m, 1H), 7.21 (dd, *J* = 8.0, 7.2 Hz, 2H), 7.12 (dd, *J* = 8.0, 7.6 Hz, 1H), 6.86 (t, *J* = 7.6 Hz, 1H), 6.74 (d, *J* = 8.0 Hz, 2H), 5.39 (d, *J* = 8.0 Hz, 1H), 5.14 (s, 1H), 4.08–3.96 (m, 3H), 3.78–3.65 (m, 4H), 1.68 (s, 9H), 0.97 (t, *J* = 6.8 Hz, 3H), 0.81–0.76 (m, 6H); ^{13}C-NMR (101 MHz, CDCl$_3$) δ 173.34, 171.55, 167.06, 166.75, 149.01, 145.13, 140.31, 129.80, 129.21, 128.73, 125.31, 124.64, 120.07, 116.70, 114.59, 84.73, 69.08, 64.90, 61.88, 61.49, 61.12, 58.18, 55.31, 42.99, 28.10, 14.15, 13.83, 13.38, 13.26; HRMS: *m*/*z* calcd. for $C_{31}H_{36}N_2O_9$ + Na, 603.2319; found, 603.2314.

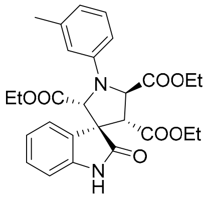

The mixed two isomers were isolated by flash chromatography (petroleum ether/ethyl acetate = 5:1) in 70% yield (62.3 mg). The *dr* value was calculated to be 5:1 from crude ^{1}H-NMR analysis of the mixture. After which, the pure major isomer **3s** was obtained as a white solid after elaborative chromatography (petroleum ether/ethyl acetate = 10:1) in 58% yield (52.1 mg). m.p. 135–138 °C ; ^{1}H-NMR (400 MHz, CDCl$_3$) δ 8.73 (s, 1H), 7.35 (d, *J* = 7.6 Hz, 1H), 7.23 (t, *J* = 8.0 Hz, 1H), 7.08 (t, *J* = 7.6 Hz, 1H), 6.99 (t, *J* = 7.6 Hz, 1H), 6.90 (d, *J* = 7.6 Hz, 1H), 6.66 (d, *J* = 7.6 Hz, 1H), 6.61 (s, 1H), 6.52 (d, *J* = 8.0 Hz, 1H), 5.41 (d, *J* = 8.4 Hz, 1H), 5.11 (s, 1H), 4.09–4.02 (m, 3H), 3.88–3.84 (m, 1H), 3.77–3.68 (m, 3H), 2.27 (s, 3H), 1.02 (t, *J* = 7.2 Hz, 3H), 0.81–0.74 (m, 6H); ^{13}C-NMR (101 MHz, CDCl$_3$) δ 176.66, 171.99, 167.45, 167.38, 145.19, 141.42, 138.41, 129.59, 128.56, 126.21, 125.70, 122.69, 121.19, 117.19, 113.43, 109.63, 68.72, 64.81, 61.42, 61.22, 61.07, 58.14, 54.64, 21.65, 13.90, 13.49, 13.40; HRMS: *m*/*z* calcd. for $C_{27}H_{30}N_2O_7$ + Na, 517.1951; found, 517.1954.

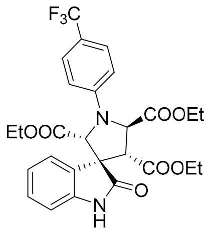

The mixed two isomers were isolated by flash chromatography (petroleum ether/ethyl acetate = 5:1) in 67% yield (55.4 mg). The *dr* value was calculated to be 5:1 from crude ^{1}H-NMR analysis of the mixture. After which, the pure major isomer **3t** was obtained as a white solid after elaborative chromatography (petroleum ether/ethyl acetate = 10:1) in 56% yield (46.2 mg). m.p. 145–147 °C; ^{1}H-NMR (400 MHz, CDCl$_3$) δ 8.62 (s, 1H), 7.47 (d, *J* = 8.8 Hz, 2H), 7.29–7.24 (m, 2H), 7.01 (t, *J* = 7.6 Hz, 1H), 6.91 (d, *J* = 7.6 Hz, 1H), 6.75 (d, *J* = 8.4 Hz, 2H), 5.45 (d, *J* = 8.4 Hz, 1H), 5.14 (s, 1H), 4.16–4.07 (m, 2H), 4.03 (d, *J* = 8.4 Hz, 1H), 3.90–3.84 (m, 1H), 3.81–3.69 (m, 3H), 1.06 (t, *J* = 7.2 Hz, 3H), 0.80 (t, *J* = 7.2 Hz, 3H), 0.76 (t, *J* = 7.2 Hz, 3H); ^{13}C-NMR (101 MHz, CDCl$_3$) δ 176.21, 171.26, 166.92, 166.85, 147.99, 141.39, 129.90, 126.10, 126.09, 125.22, 124.54 (d, J_{CF} = 272.7 Hz), 122.82, 121.67 (d, J_{CF} = 33.3 Hz), 115.51, 109.75, 68.63, 64.62, 61.86, 61.61, 61.46, 58.06, 54.78, 13.90, 13.45, 13.40; HRMS: *m*/*z* calcd. for $C_{27}H_{27}F_3N_2O_7$ + Na, 571.1668; found, 571.1671.

3. Synthetic Transformations to Access Other Drug-Like Spirocyclic Scaffolds 3

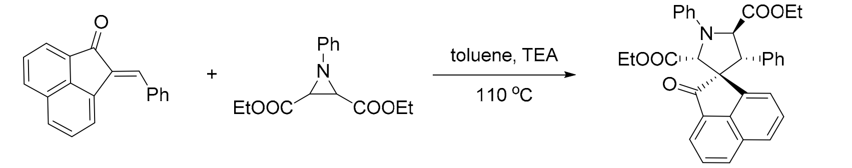

olefinic acenaphthene **2a** **3u**, 76% (68%) yield, 8:1 dr

A mixture of olefinic acenaphthene (1.1 mmol), aziridine **2a** (1.0 mmol) and additive TEA (0.5 mmol) in toluene (2 mL) was refluxed at 110 °C under an open atmosphere. The reaction mixture would be cooled to room tempreture until most of olefinic acenaphthene was consumed (monitored by TLC). Then the reaction mixture was concentrated and the residue was isolated by flash chromatography on silica gel (petroleum ether/ethyl acetate = 5:1) to give the mixed two isomers in 76% yield (75.4 mg). The *dr* value was calculated to be 8:1 from crude ^{1}H-NMR analysis of the mixture. After which, the pure major isomer **3u** was obtained as a white solid after elaborative

chromatography (petroleum ether/ethyl acetate = 10:1) in 68% yield (66.9 mg). m.p. 154–156 °C; ^{1}H-NMR (400 MHz, $CDCl_3$) δ 7.94–7.91 (m, 2H), 7.76–7.71 (m, 2H), 7.61–7.55 (m, 2H), 7.23 (t, *J* = 8.0 Hz, 2H), 7.00–6.97 (m, 2H), 6.87–6.81 (m, 4H), 6.76 (d, *J* = 8.0 Hz, 2H), 5.53 (d, *J* = 10.4 Hz, 1H), 5.44 (s, 1H), 4.48 (d, *J* = 10.0 Hz, 1H), 4.03–3.93 (m, 2H), 3.46–3.36 (m, 2H), 0.88 (t, *J* = 7.2 Hz, 3H), 0.04 (t, *J* = 7.2 Hz, 3H); ^{13}C-NMR (101 MHz, $CDCl_3$) δ 201.65, 171.97, 168.09, 145.61, 142.19, 134.83, 132.95, 132.84, 131.87, 130.23, 128.76, 128.22, 128.05, 127.81, 127.76, 127.62, 125.07, 123.99, 121.86, 119.30, 115.42, 68.07, 67.71, 66.11, 61.22, 60.41, 57.32, 13.82, 12.78; HRMS: *m*/*z* calcd. for $C_{33}H_{29}NO_5$ + Na, 542.1943; found, 542.1945.

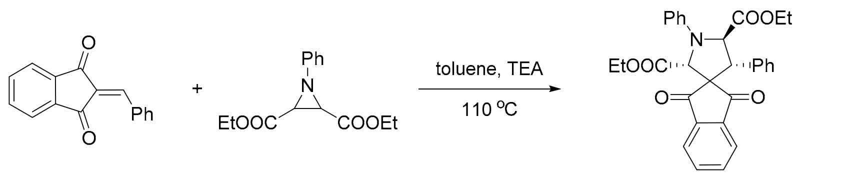

olefinic indenedione **2a** **3v**, 90% (72%) yield, 4:1 dr

A mixture of olefinic indenedione (1.1 mmol), aziridine **2a** (1.0 mmol) and additive TEA (0.5 mmol) in toluene (2 mL) was refluxed at 110 °C under an open atmosphere. The reaction mixture would be cooled to room tempreture until most of olefinic indenedione was consumed (monitored by TLC). Then the reaction mixture was concentrated and the residue was isolated by flash chromatography on silica gel (petroleum ether/ethyl acetate = 5:1) to give the mixed two isomers in 90% yield (85.3 mg). The *dr* value was calculated to be 4:1 from crude ^{1}H-NMR analysis of the mixture. After which, the pure major isomer **3v** was obtained as a white solid after elaborative chromatography (petroleum ether/ethyl acetate = 10:1) in 72% yield (68.2 mg). The *dr* value was calculated to be 4:1 by ^{1}H-NMR analysis of the crude reaction mixture; m.p. 133–135 °C; ^{1}H-NMR (400 MHz, $CDCl_3$) δ 7.94 (d, *J* = 7.6 Hz, 1H), 7.75–7.67 (m, 3H), 7.22–7.15 (m, 4H), 7.10–7.05 (m, 3H), 6.79 (t, *J* = 7.6 Hz, 1H), 6.70 (d, *J* = 7.6 Hz, 2H), 5.57 (d, *J* = 10.0 Hz, 1H), 5.33 (s, 1H), 4.23 (d, *J* = 10.4 Hz, 1H), 4.01–3.92 (m, 2H), 3.84–3.72 (m, 2H), 0.89 (t, *J* = 7.2 Hz, 3H), 0.64 (t, *J* = 7.2 Hz, 3H); ^{13}C-NMR (101 MHz, $CDCl_3$) δ 198.08, 197.65, 171.57, 168.05, 145.09, 142.18, 142.05, 136.00, 135.74, 131.84, 128.71, 128.64, 128.46, 128.30, 123.27, 123.08, 119.11, 115.14, 66.58, 66.13, 65.76, 61.23, 61.18, 56.29, 13.82, 13.33; HRMS: *m*/*z* calcd. for $C_{30}H_{27}NO_6$ + Na, 520.1736; found, 520.1733.

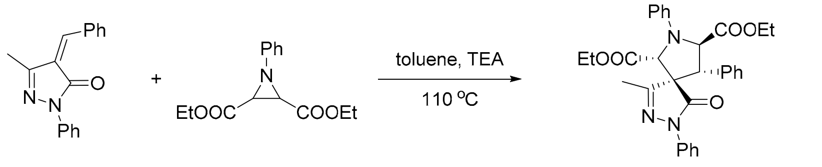

olefinic pyrazolone **2a** **3w**, 93% (78%) yield, 5:1 dr

A mixture of olefinic pyrazolone (1.1 mmol), aziridine **2a** (1.0 mmol) and additive TEA (0.5 mmol) in toluene (2 mL) was refluxed at 110 °C under an open atmosphere. The reaction mixture would be cooled to room tempreture until most of olefinic pyrazolone was consumed (monitored by TLC). Then the reaction mixture was concentrated and the residue was isolated by flash chromatography on silica gel (petroleum ether/ethyl acetate = 5:1) to give the mixed two isomers in 93% yield (92.4 mg). The *dr* value was calculated to be 5:1 from crude ^{1}H-NMR analysis of the mixture. After which, the pure major isomer **3w** was obtained as a white solid after elaborative chromatography (petroleum ether/ethyl acetate = 10:1) in 78% yield (76.8 mg). m.p. 165–167 °C; ^{1}H-NMR (400 MHz, $CDCl_3$) δ 7.49 (d, *J* = 8.0 Hz, 2H), 7.32 (d, *J* = 6.8 Hz, 2H), 7.27 (s, 1H), 7.25–7.20 (m, 6H), 7.09 (t, *J* = 7.2 Hz, 1H), 6.82 (t, *J* = 7.2 Hz, 1H), 6.71 (d, *J* = 8.0 Hz, 2H), 5.62 (d, *J* = 9.6 Hz, 1H), 5.12 (s, 1H), 4.08–4.05 (m, 1H), 4.01 (d, *J* = 7.2 Hz, 1H), 3.98–3.90 (m, 3H), 2.47 (s, 3H), 0.94 (q, *J* = 6.8 Hz, 6H); ^{13}C-NMR (101 MHz, $CDCl_3$)δ 172.16, 169.60, 167.44, 157.03, 144.78, 137.18, 131.10, 128.86, 128.82, 128.64, 128.39, 125.23, 119.48, 118.97, 115.12, 65.83, 64.85, 64.69, 61.71, 61.43, 55.14, 13.88, 13.82, 13.74; HRMS: *m*/*z* calcd. for $C_{31}H_{31}N_3O_5$ + Na, 548.2161; found, 548.2159.

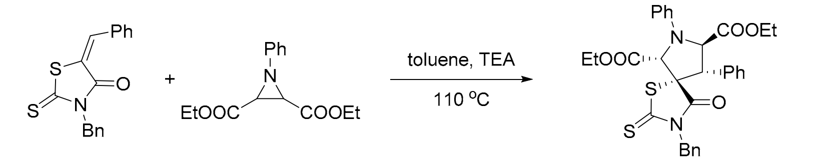

olefinic rhodanine **2a** **3x**, 72% (69%) yield, >20:1 dr

A mixture of olefinic rhodanine (1.1 mmol), aziridine **2a** (1.0 mmol) and additive TEA (0.5 mmol) in toluene (2 mL) was refluxed at 110 °C under an open atmosphere. The reaction mixture would be cooled to room tempreture until most of olefinic rhodanine was consumed (monitored by TLC). Then the reaction mixture was concentrated and the residue was isolated by flash chromatography on silica gel (petroleum ether/ethyl acetate = 5:1) to give the mixed two isomers in 72% yield (78.1 mg). The *dr* value was calculated to be >20:1 from crude ^{1}H-NMR analysis of the mixture. After which, the pure major isomer **3x** was obtained as a white solid after elaborative chromatography (petroleum ether/ethyl acetate = 10:1) in 69% yield (74.9 mg). m.p. 162–165 °C; ^{1}H-NMR (400 MHz, $CDCl_3$) δ 7.31–7.27 (m, 5H), 7.25–7.10 (m, 7H), 6.85 (t, *J* = 7.2 Hz, 1H), 6.67 (d, *J* = 7.6 Hz, 2H), 5.54 (s, 1H), 5.09 (d, *J* = 10.0 Hz, 1H), 4.69 (dd, *J* = 41.6, 14.0 Hz, 2H), 4.55 (d, *J* = 10.0 Hz, 1H), 4.07–4.03 (m, 1H), 3.94–3.85 (m, 3H), 0.94 (t, *J* = 7.2 Hz, 3H), 0.84 (t, *J* = 7.2 Hz, 3H); ^{13}C-NMR (101 MHz, $CDCl_3$) δ 172.68, 170.92, 169.02, 166.92, 144.85, 134.51, 131.38, 128.96, 128.88, 128.82, 128.78, 128.68, 128.66, 128.29, 120.49, 116.10, 69.76, 66.62, 61.87, 61.51, 57.08, 45.52, 43.00, 13.87, 13.74; HRMS: *m*/*z* calcd. for $C_{31}H_{30}N_2O_5S_2$ + Na, 597.1494; found, 597.1497.

4. Crystal Data of 3a

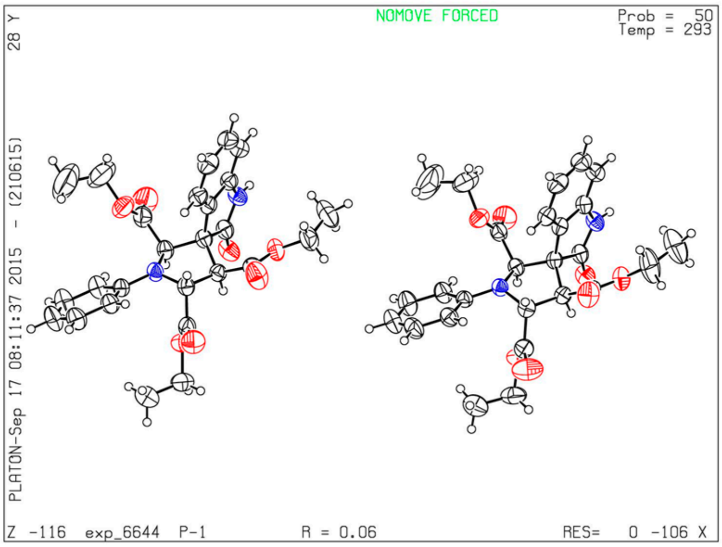

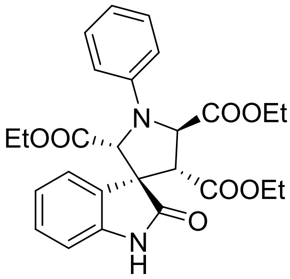

3a

Bond precision:	C-C = 0.0036 A	Wavelength = 1.54184	
Cell:	A = 7.7992(5)	B = 15.5450(9)	C = 20.9801(11)
	Alpha = 100.568(5)	Beta = 95.160(5)	Gamma = 94.057(5)
Temperature:	293 K		
	Calculated	Reported	
Volume	2480.4(3)	2480.4(3)	
Space group	P-1	P-1	
Hall group	-P-1		
Moiety formula	C26 H28 N2 O7		
Sum formula	C26 H28 N2 O7	C52 H56 N4 O14	
Mr	480.50	961.01	
Dx, g cm^{-3}	1.287	1.287	
Z	4	2	
Mu (mm^{-1})	0.779	0.779	
F000	1016.0	1016.0	
F000′	1019.36		

h,k,lmax	9,18,25	9, 18, 25
Nref	8895	8879
Tmin,Tmax	0.940,0.947	0.940, 0.947
Tmin′	0.940	
Correction method= # Reported T Limits: Tmin = 0.940 Tmax = 0.947		
AbsCorr = MULTI-SCAN		
Data completeness = 0.998		Theta(max) = 67.240
R(reflections) = 0.0553(6288)		wR2(reflections) = 0.1515(8879)
S = 1.044		Npar = 637

5. NMR Spectra

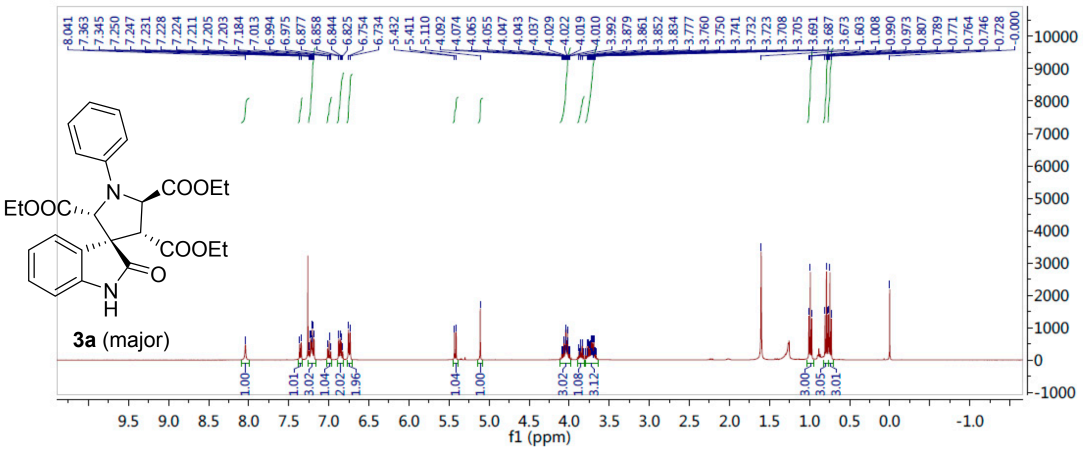

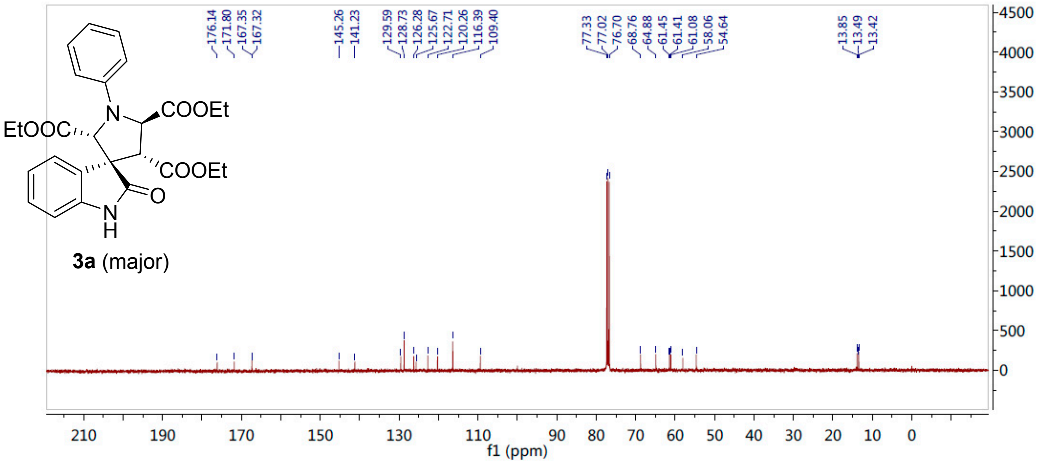

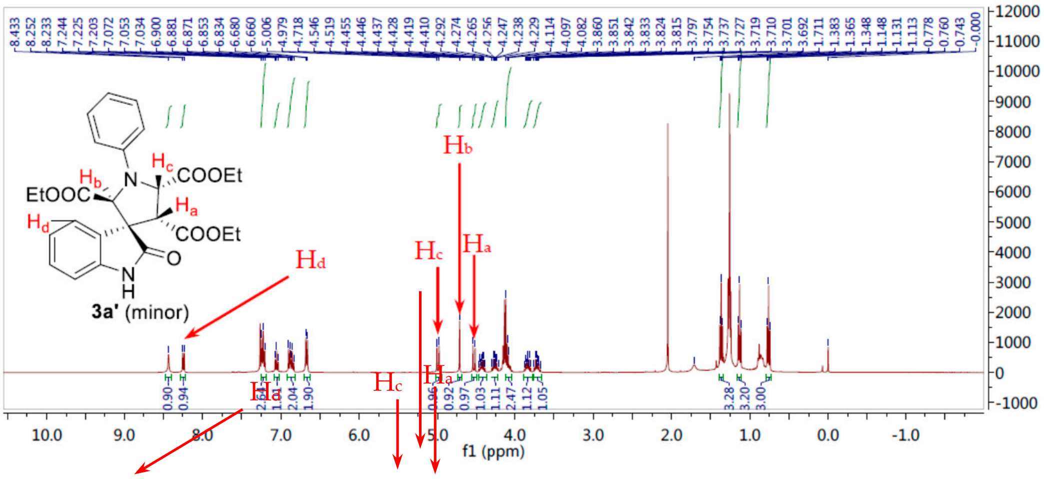

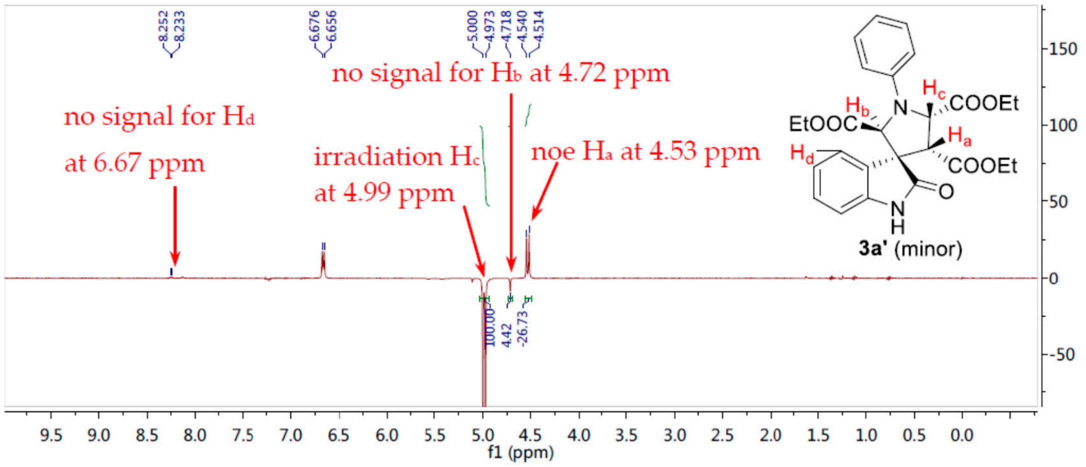
no signal for Hd
at 6.67 ppm
no signal for Hb at 4.72 ppm
irradiation Hc
at 4.99 ppm
noe Ha at 4.53 ppm
3a' (minor)
f1 (ppm)

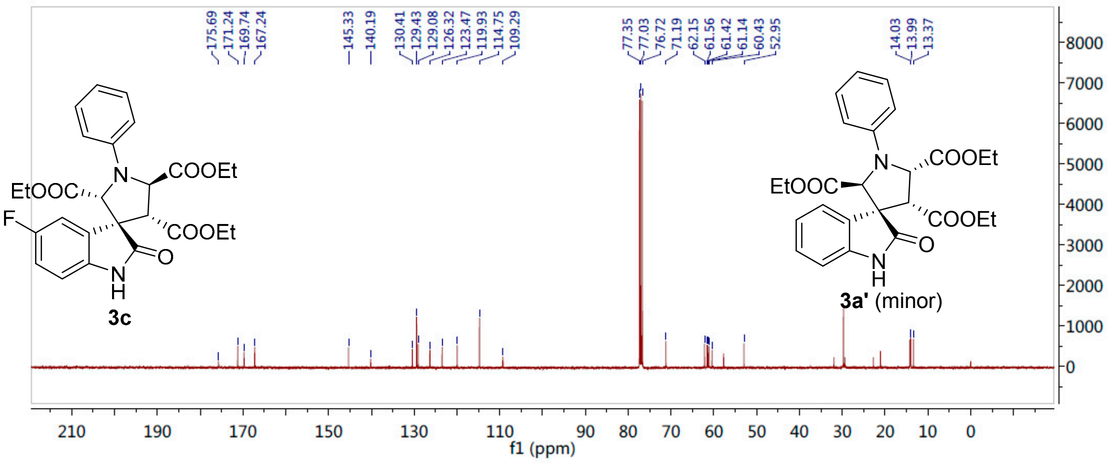
3c
3a' (minor)
f1 (ppm)

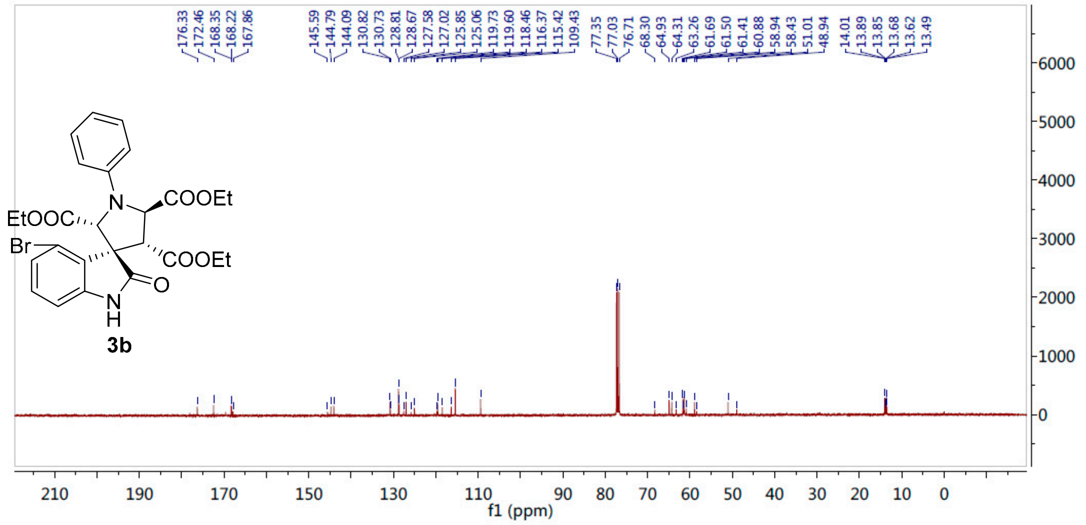
3b
f1 (ppm)

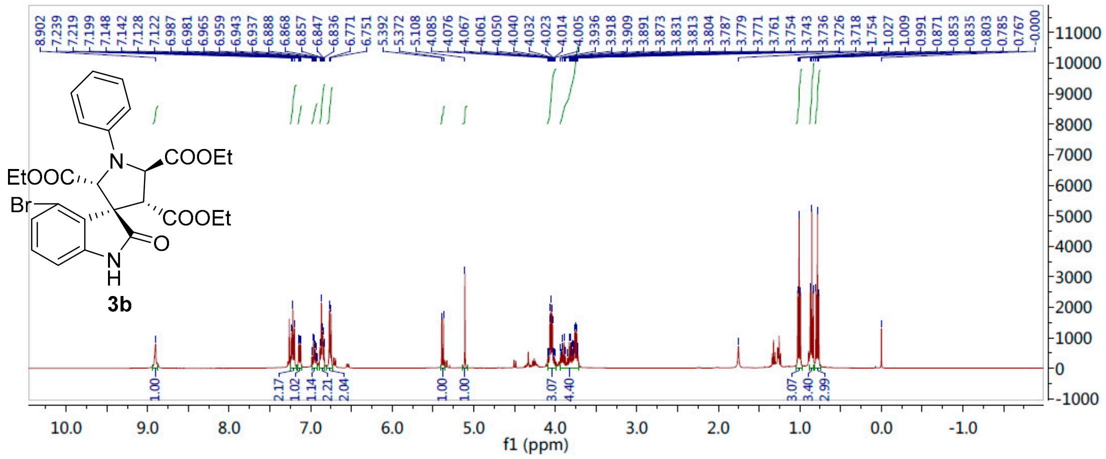

3b

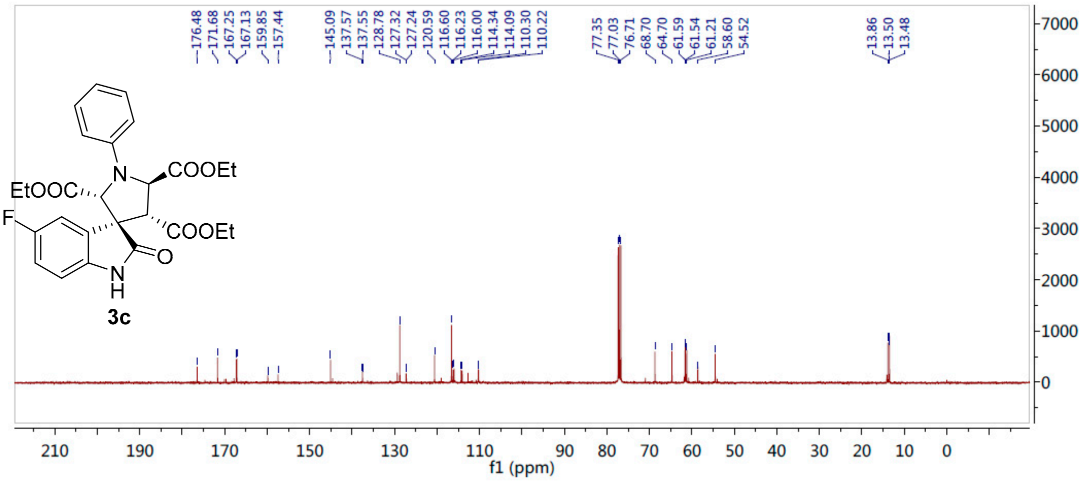

3c

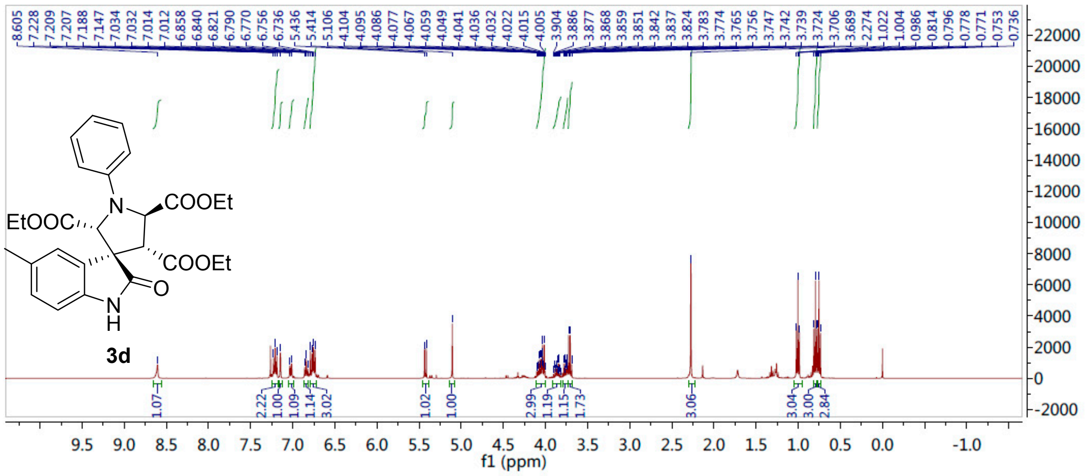

3d

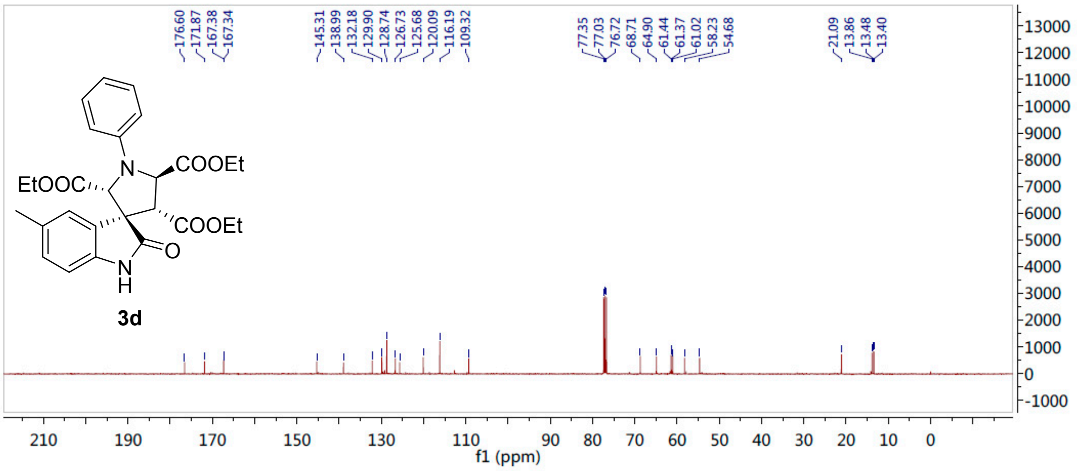
176.60
171.87
167.38
167.34
145.31
138.99
132.18
129.90
128.74
126.73
125.68
120.09
116.19
109.32
77.35
77.03
76.72
68.71
64.90
61.44
61.37
61.02
58.23
54.68
21.09
13.86
13.48
13.40
EtOOC
COOEt
COOEt
N
O
N
H
3d
f1 (ppm)

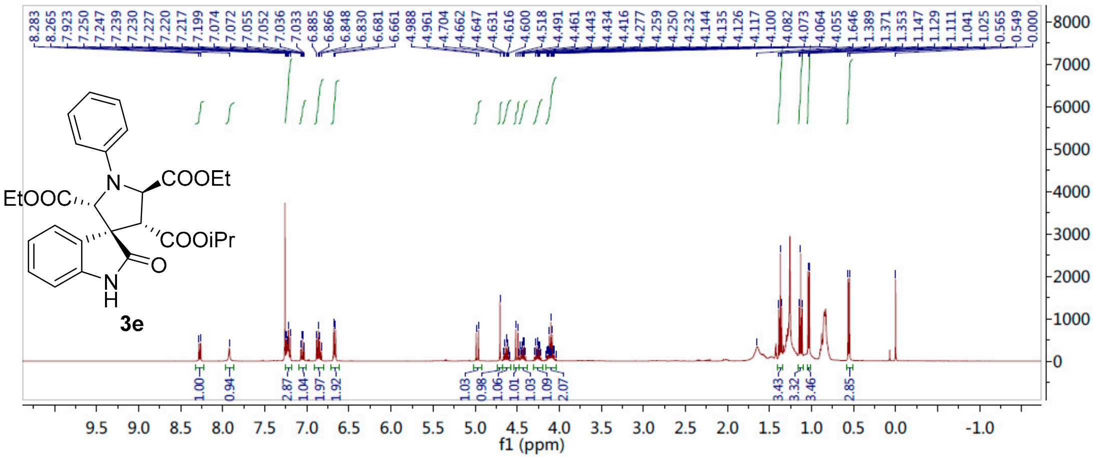
EtOOC
COOEt
COOiPr
N
O
N
H
3e
f1 (ppm)

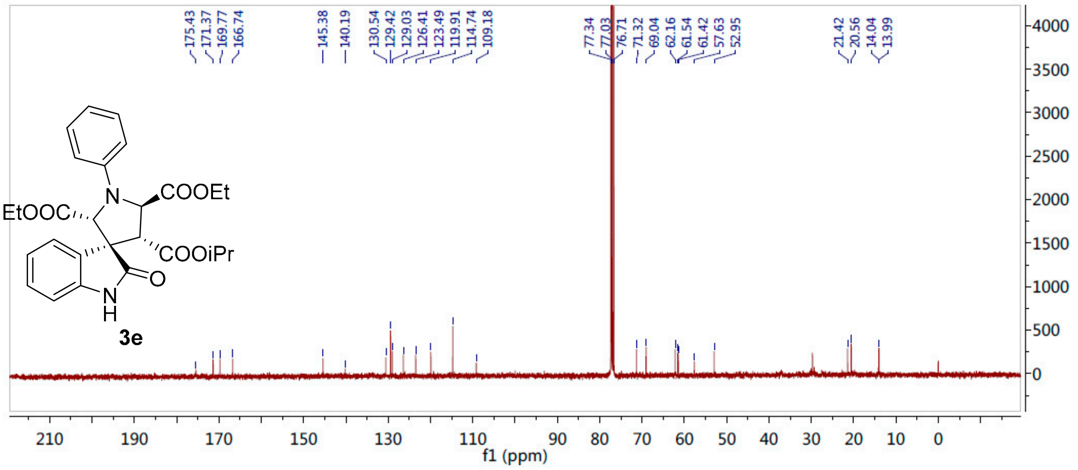
175.43
171.37
169.77
166.74
145.38
140.19
130.54
129.42
129.03
126.41
123.49
119.91
114.74
109.18
77.34
77.03
76.71
71.32
69.04
62.16
61.54
61.42
57.63
52.95
21.42
20.56
14.04
13.99
EtOOC
COOEt
COOiPr
N
O
N
H
3e
f1 (ppm)

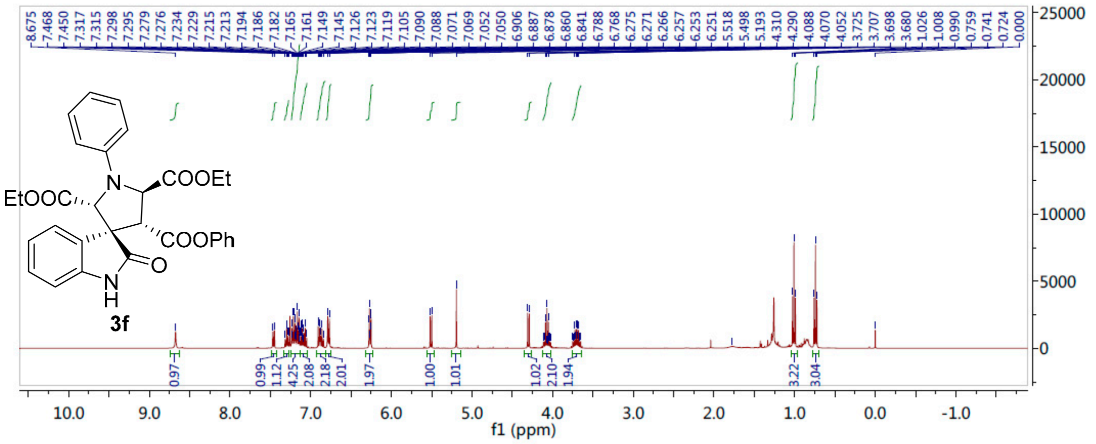

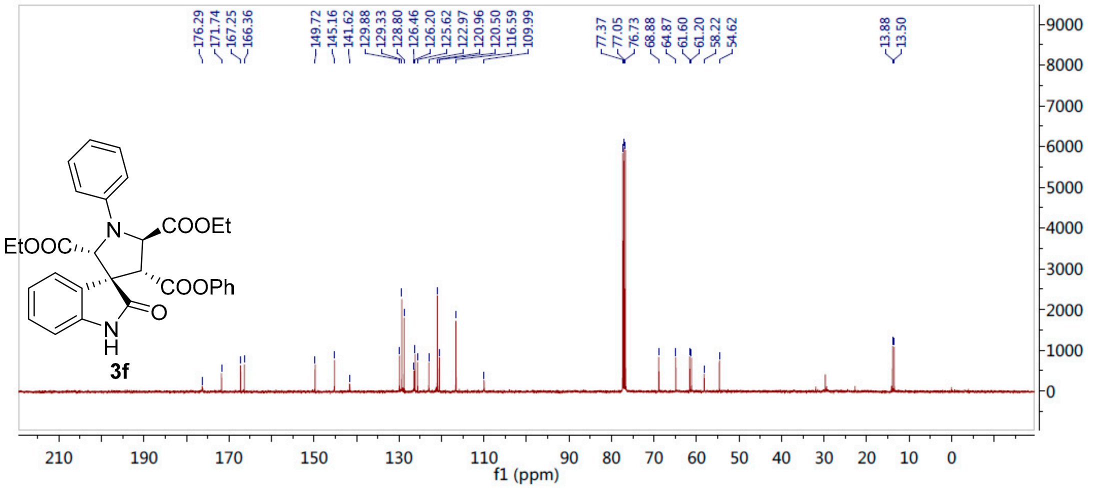

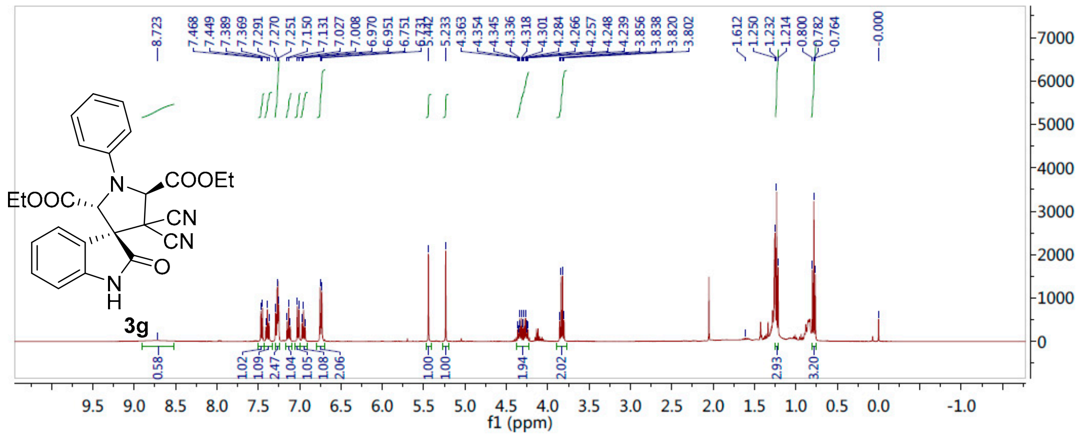

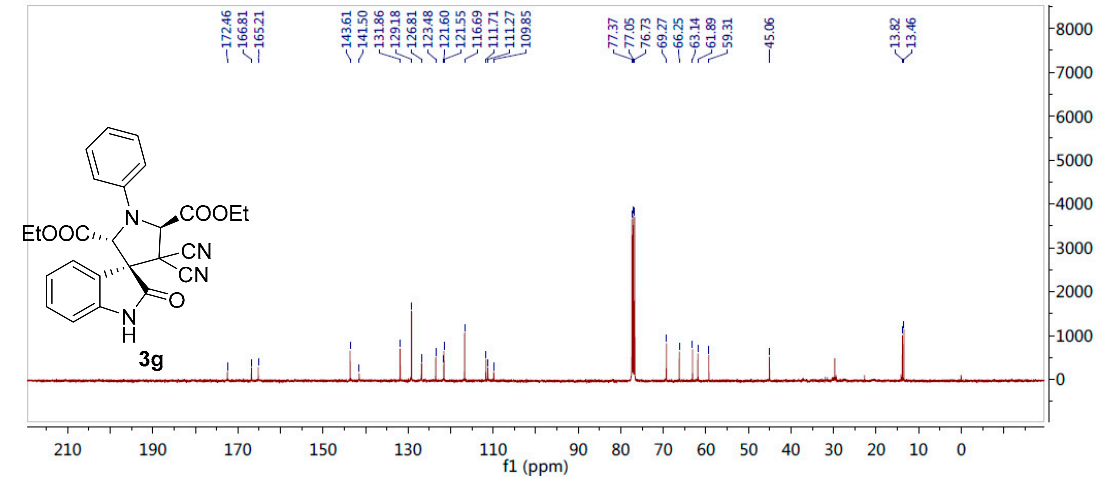

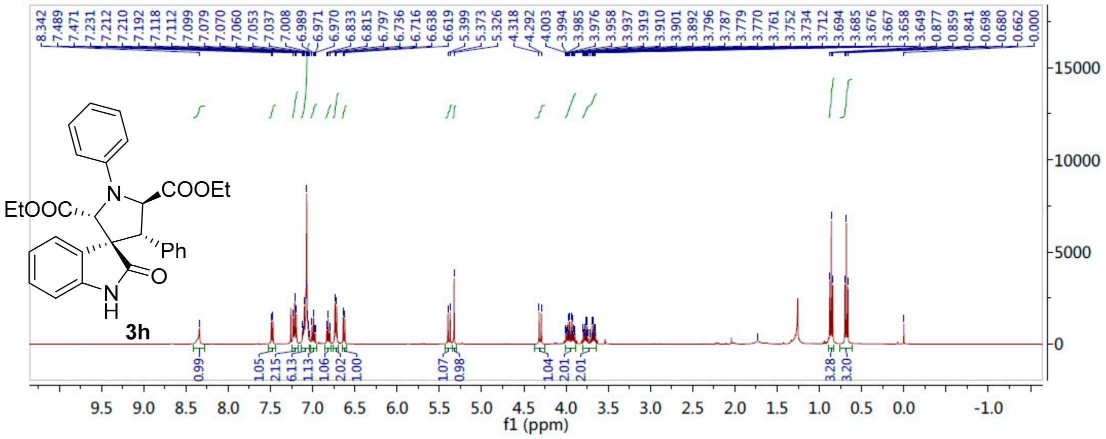

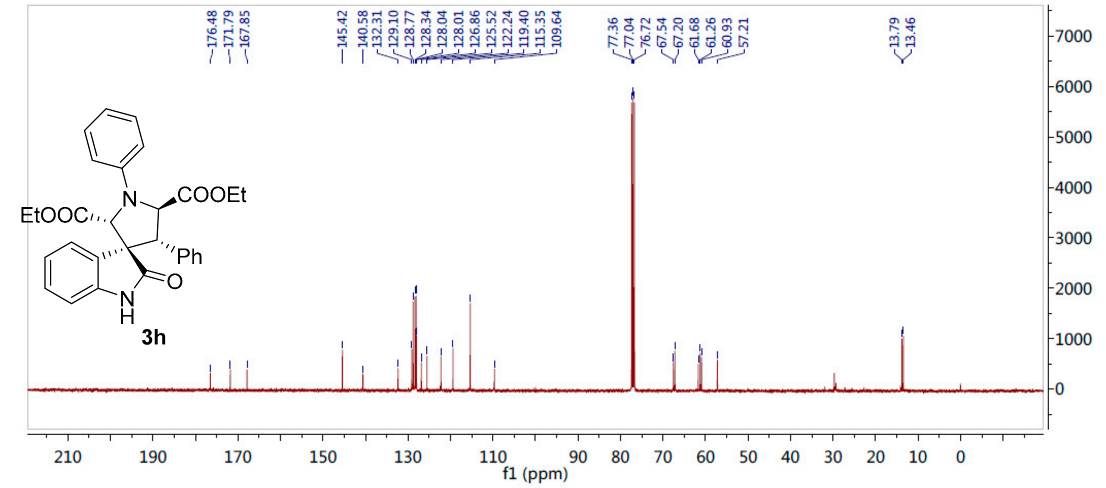

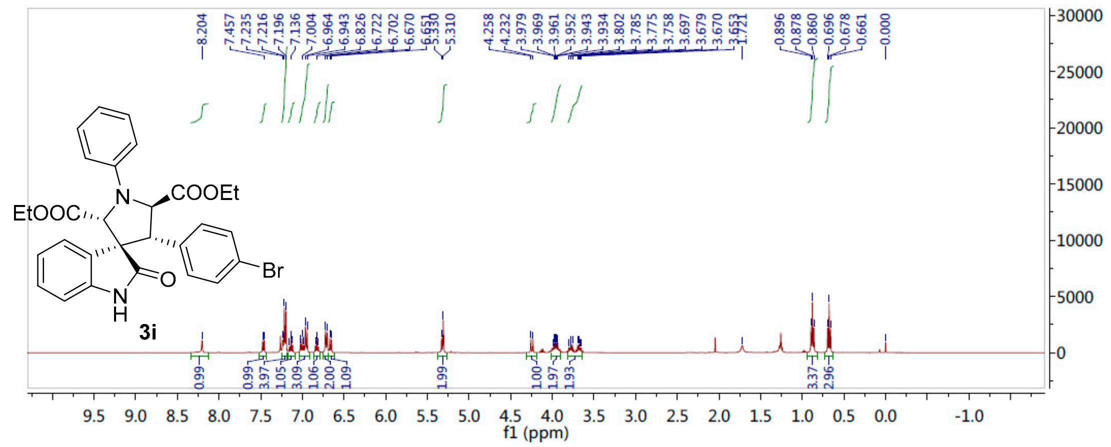
8.204
7.457
7.235
7.216
7.196
7.136
7.004
6.964
6.943
6.826
6.722
6.702
6.670
6.651
5.330
5.310
4.258
4.232
3.979
3.969
3.961
3.952
3.943
3.934
3.802
3.785
3.775
3.758
3.697
3.679
3.670
3.653
1.721
0.896
0.878
0.860
0.696
0.678
0.661
0.000
0.99
0.99
3.97
1.05
3.09
1.06
2.00
1.09
1.99
1.00
1.97
1.93
3.37
2.96
EtOOC
N
COOEt
Br
O
N
H
3i
f1 (ppm)

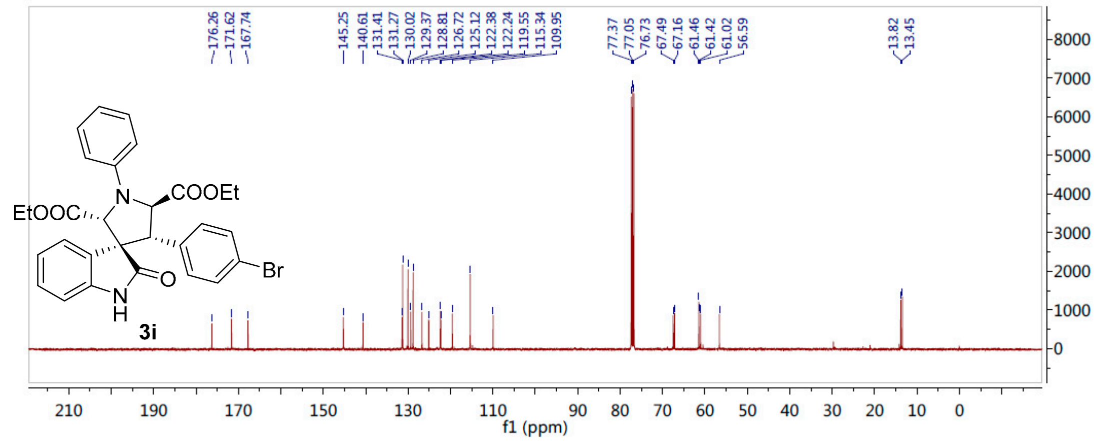
176.26
171.62
167.74
145.25
140.61
131.41
131.27
130.02
129.37
128.81
126.72
125.12
122.38
122.24
119.55
115.34
109.95
77.37
77.05
76.73
67.49
67.16
61.46
61.42
61.02
56.59
13.82
13.45
EtOOC
N
COOEt
Br
O
N
H
3i
f1 (ppm)

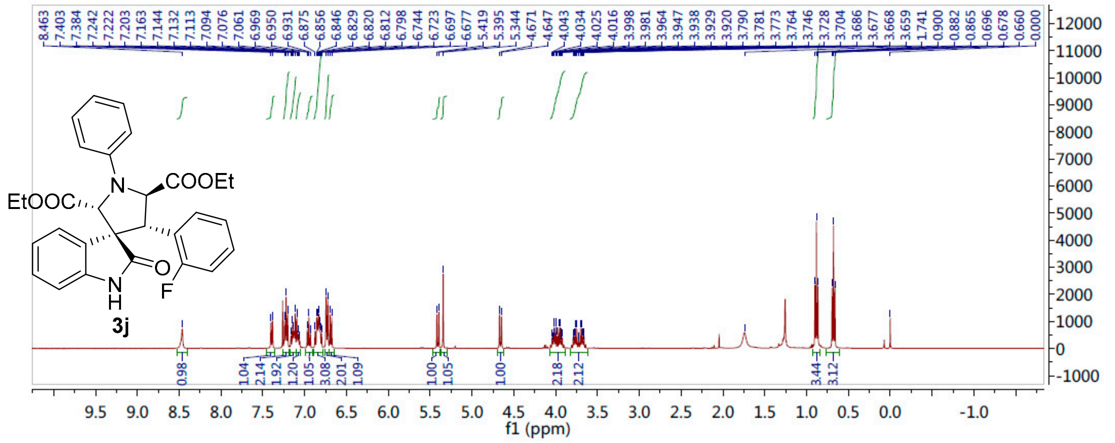
8.463
7.403
7.384
7.242
7.222
7.203
7.163
7.144
7.132
7.113
7.094
7.076
7.061
6.969
6.950
6.931
6.875
6.856
6.846
6.829
6.820
6.812
6.798
6.744
6.723
6.697
6.677
5.419
5.395
5.344
4.671
4.647
4.043
4.034
4.025
4.016
3.998
3.981
3.964
3.947
3.938
3.929
3.920
3.790
3.781
3.773
3.764
3.746
3.728
3.704
3.686
3.677
3.668
3.659
1.741
0.900
0.882
0.865
0.696
0.678
0.660
0.000
0.98
1.04
2.14
1.92
1.20
1.05
3.08
2.01
1.09
1.00
1.05
1.00
2.18
2.12
3.44
3.12
EtOOC
N
COOEt
O
N
H
F
3j
f1 (ppm)

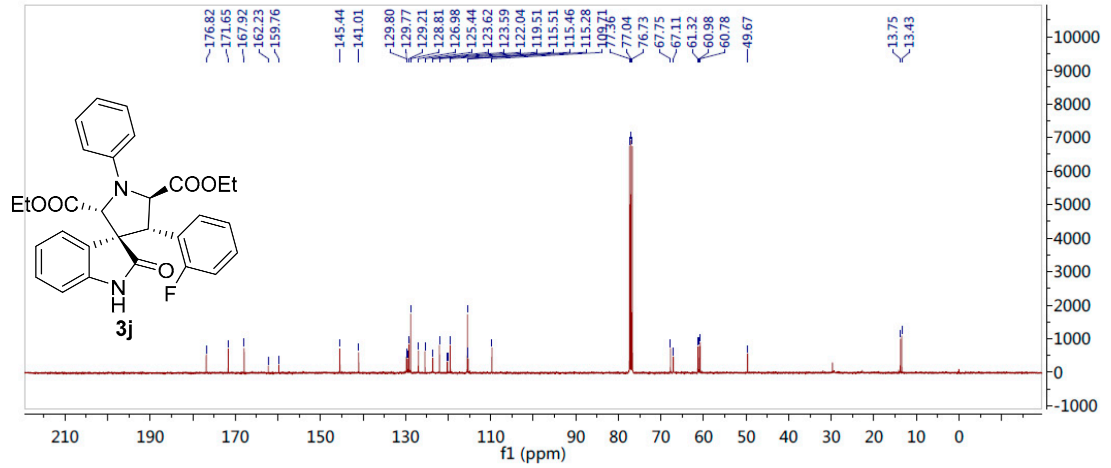
176.82
171.65
167.92
162.23
159.76
145.44
141.01
129.80
129.77
129.21
128.81
126.98
125.44
123.62
123.59
122.04
119.51
115.51
115.46
115.28
109.71
77.36
77.04
76.73
67.75
67.11
61.32
60.98
60.78
49.67
13.75
13.43
EtOOC
N
COOEt
O
N
H
F
3j
f1 (ppm)

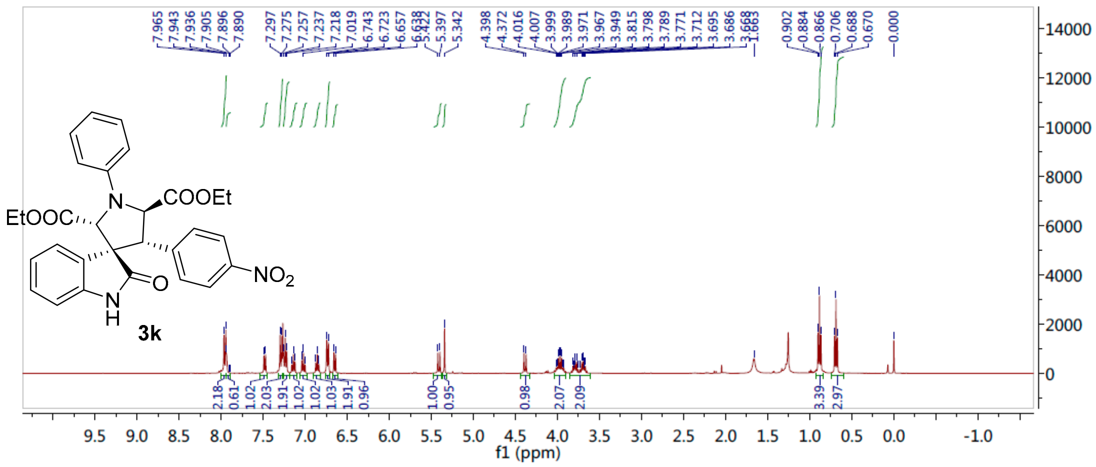

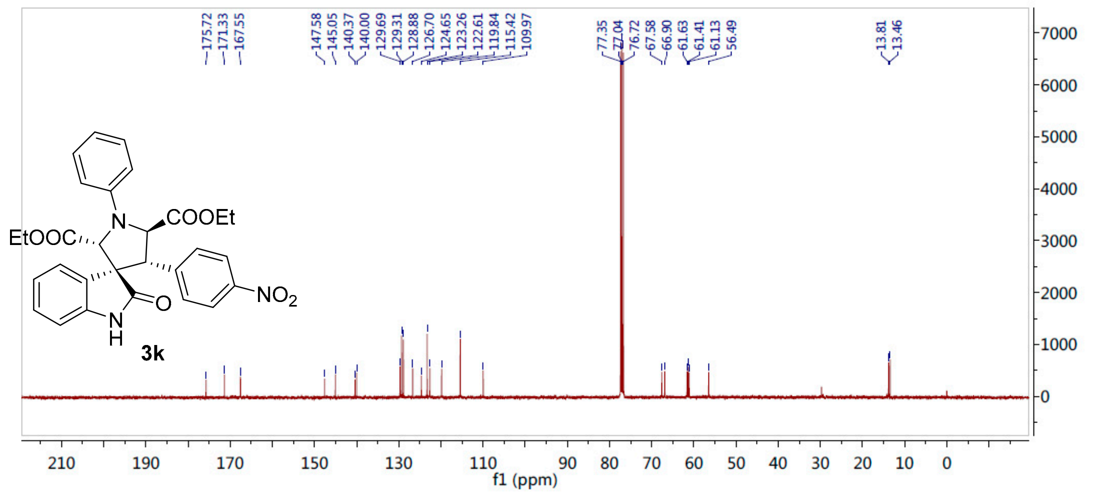

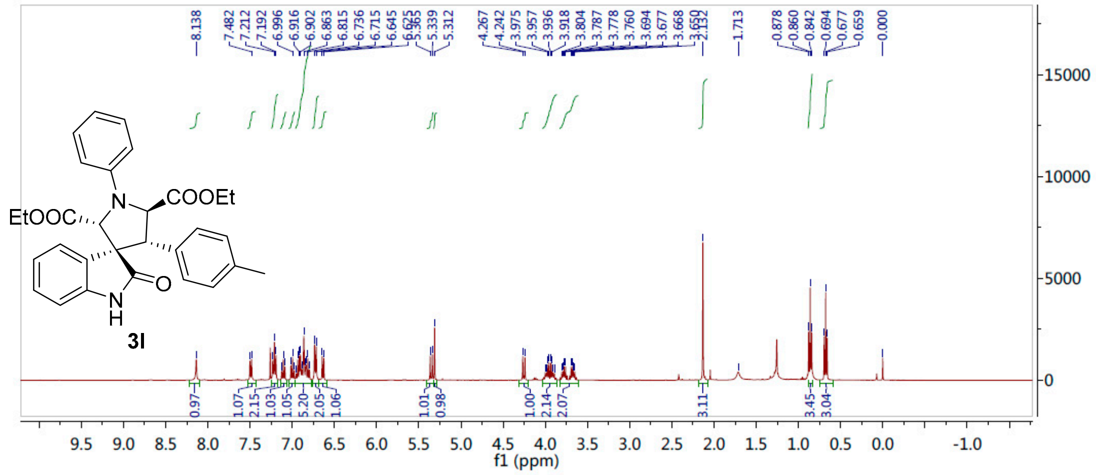

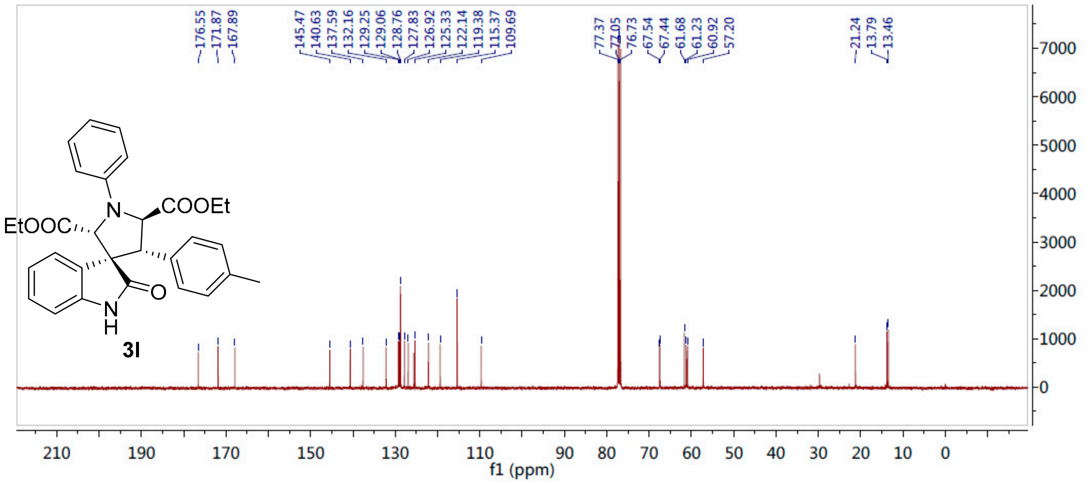
176.55
171.87
167.89
145.47
140.63
137.59
132.16
129.25
129.06
128.76
127.83
126.92
125.33
122.14
119.38
115.37
109.69
77.37
77.05
76.73
67.54
67.44
61.68
61.23
60.92
57.20
21.24
13.79
13.46
EtOOC
N
COOEt
N
H
O
3l
f1 (ppm)

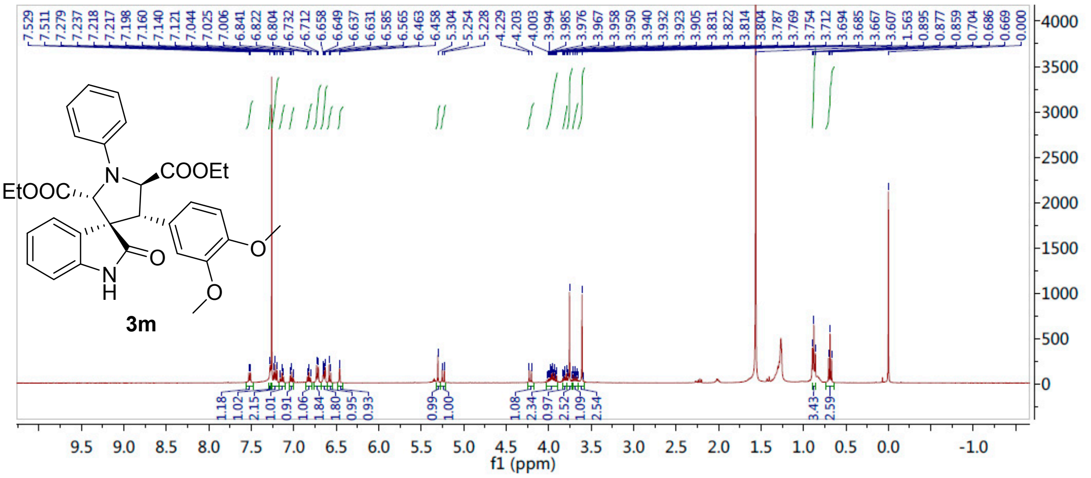
7.529
7.511
7.279
7.237
7.218
7.217
7.198
7.160
7.140
7.121
7.044
7.025
7.006
6.841
6.822
6.804
6.732
6.712
6.658
6.649
6.637
6.631
6.585
6.565
6.463
6.458
5.304
5.254
5.228
4.229
4.203
4.003
3.994
3.985
3.976
3.967
3.958
3.950
3.940
3.932
3.923
3.905
3.831
3.822
3.814
3.804
3.787
3.769
3.754
3.712
3.694
3.685
3.667
3.607
1.563
0.895
0.877
0.859
0.704
0.686
0.669
0.000
EtOOC
N
COOEt
N
H
O
3m
f1 (ppm)

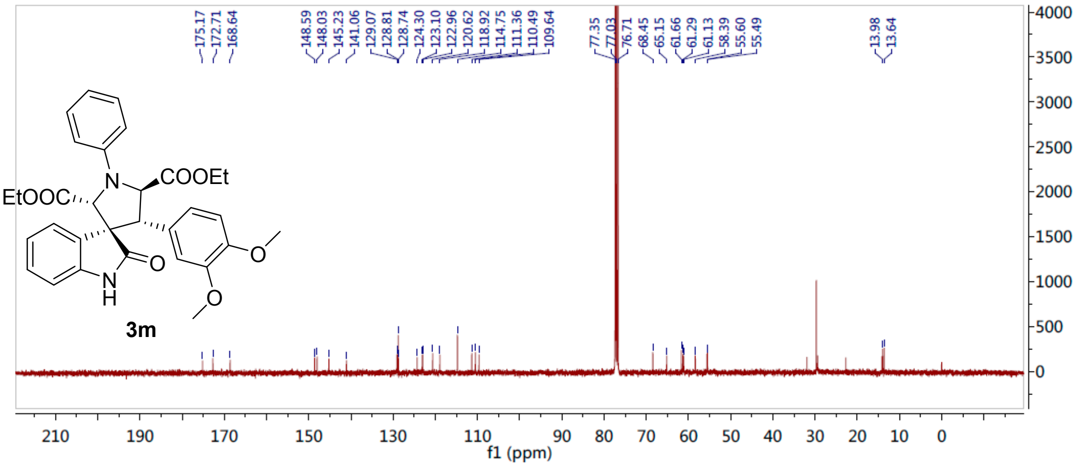
175.17
172.71
168.64
148.59
148.03
145.23
141.06
129.07
128.81
128.74
124.30
123.10
122.96
120.62
118.92
114.75
111.36
110.49
109.64
77.35
77.03
76.71
68.45
65.15
61.66
61.29
61.13
58.39
55.60
55.49
13.98
13.64
EtOOC
N
COOEt
N
H
O
3m
f1 (ppm)

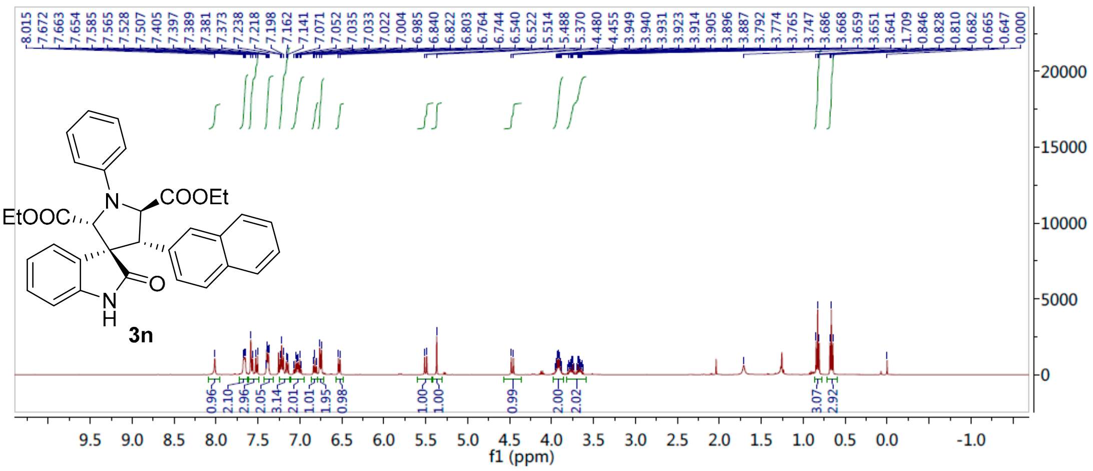
EtOOC
COOEt
N
O
N
H
3n
f1 (ppm)

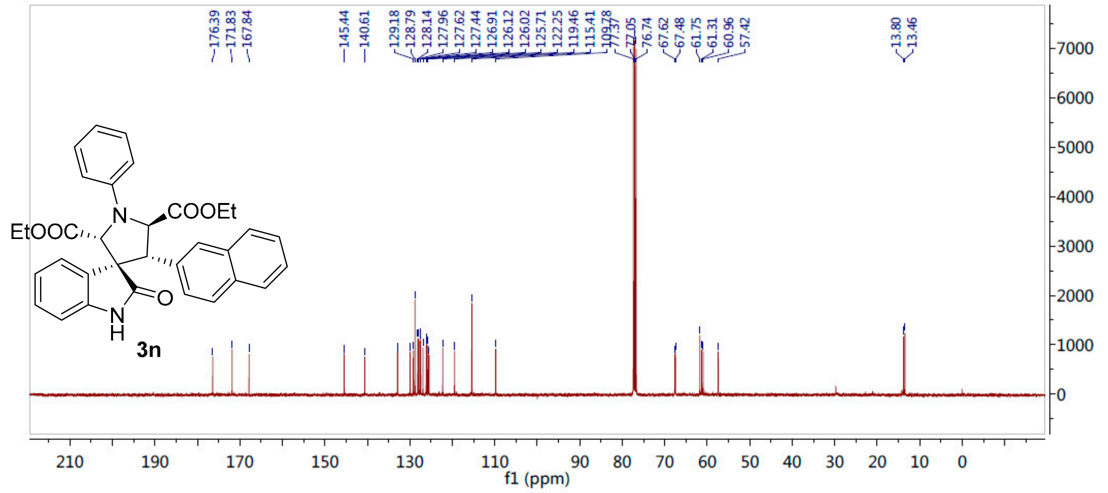
EtOOC
COOEt
N
O
N
H
3n
f1 (ppm)

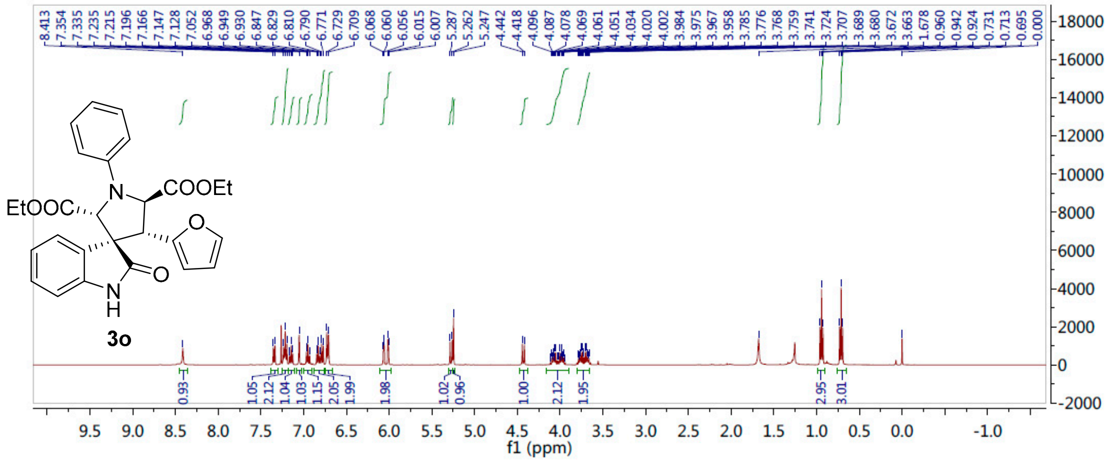
EtOOC
COOEt
N
O
N
H
3o
f1 (ppm)

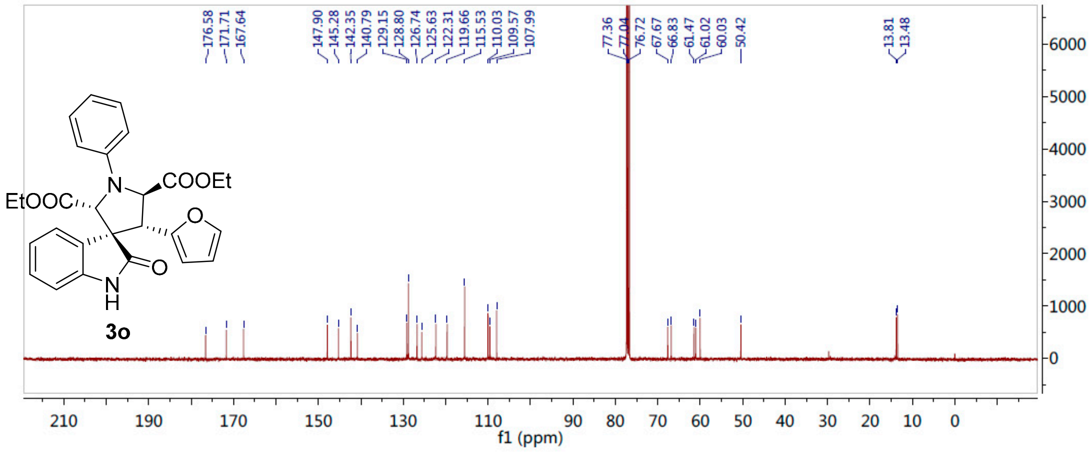
176.58
171.71
167.64
147.90
145.28
142.35
140.79
129.15
128.80
126.74
125.63
122.31
119.66
115.53
110.03
109.57
107.99
77.36
77.04
76.72
67.67
66.83
61.47
61.02
60.03
50.42
13.81
13.48
EtOOC
N
COOEt
O
O
N
H
3o
f1 (ppm)

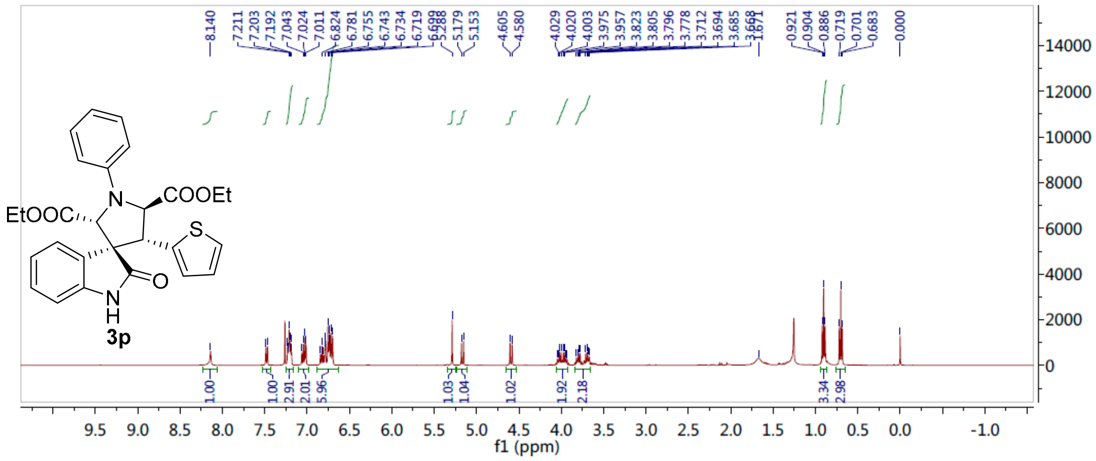
8.140
7.211
7.203
7.192
7.043
7.024
7.011
6.824
6.781
6.755
6.743
6.734
6.719
5.179
5.153
4.605
4.580
4.029
4.020
4.003
3.975
3.957
3.823
3.805
3.796
3.778
3.712
3.694
3.685
0.921
0.904
0.886
0.719
0.701
0.683
0.000
EtOOC
N
COOEt
S
O
N
H
3p
f1 (ppm)

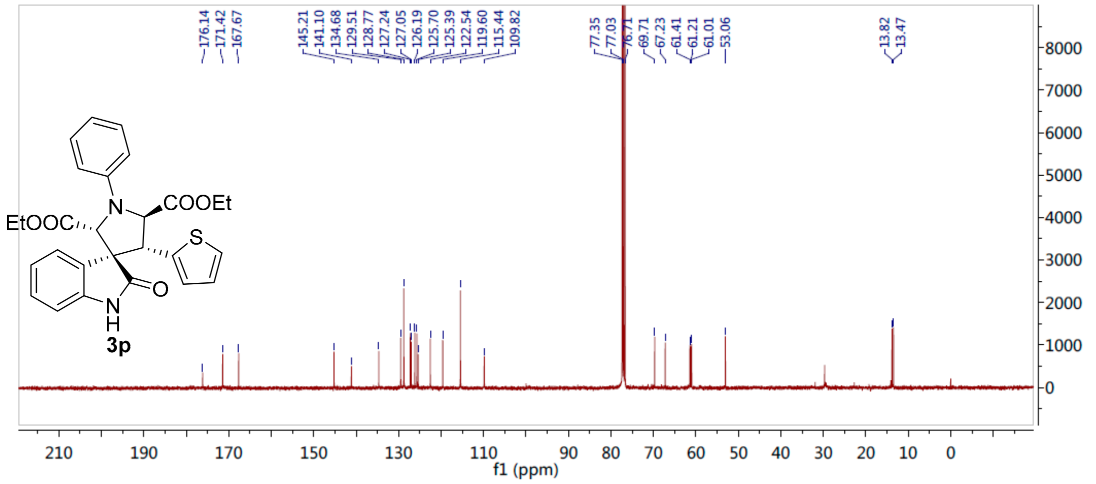
176.14
171.42
167.67
145.21
141.10
134.68
129.51
128.77
127.24
127.05
126.19
125.70
125.39
122.54
119.60
115.44
109.82
77.35
77.03
76.71
69.71
67.23
61.41
61.21
61.01
53.06
13.82
13.47
EtOOC
N
COOEt
S
O
N
H
3p
f1 (ppm)

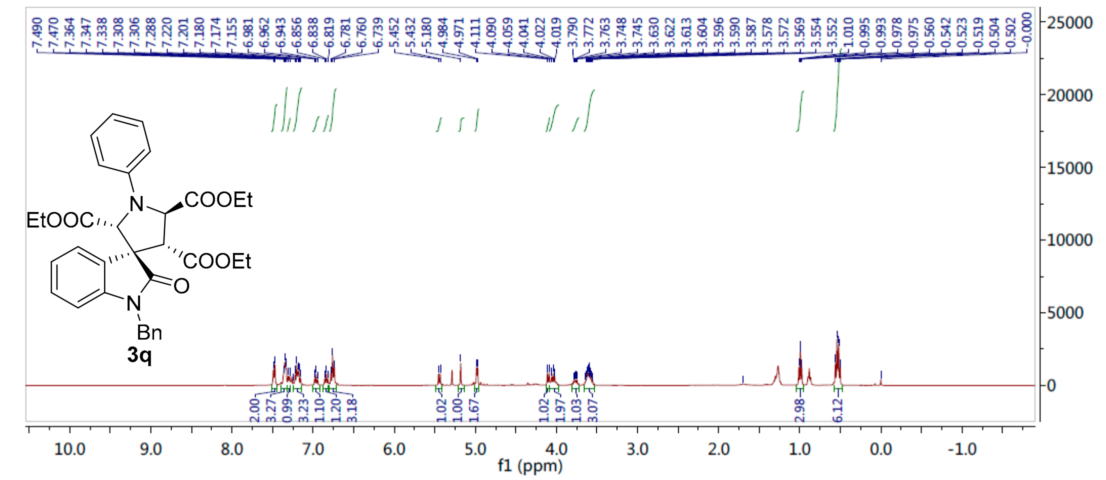
EtOOC
COOEt
COOEt
N
O
Bn
3q
f1 (ppm)

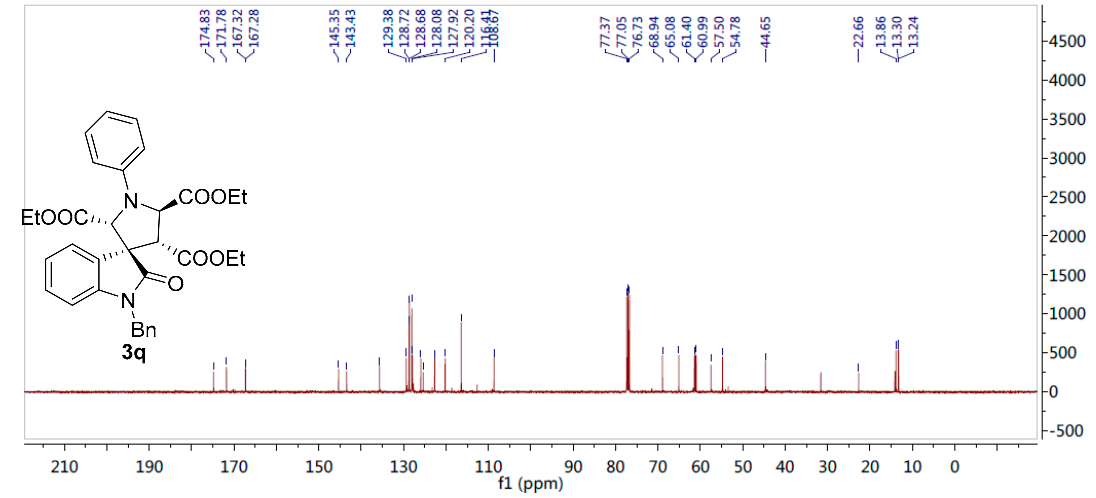
EtOOC
COOEt
COOEt
N
O
Bn
3q
f1 (ppm)

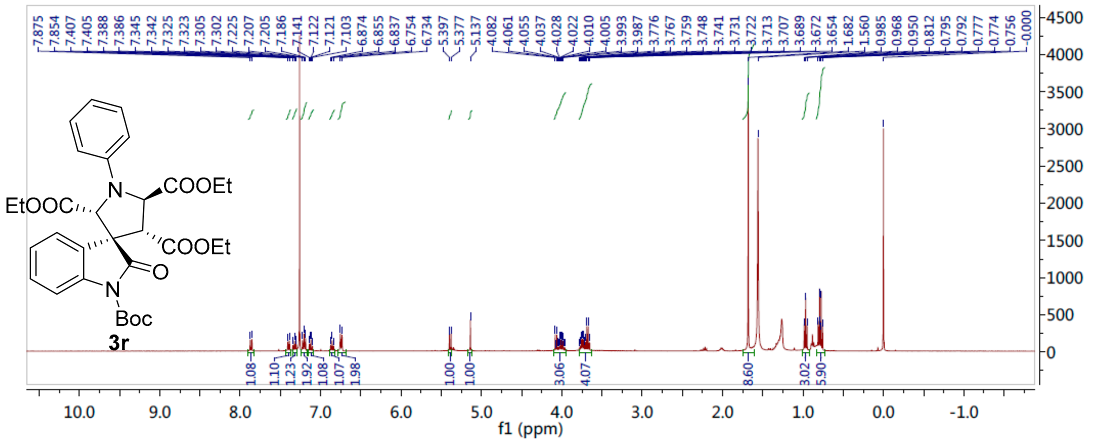
EtOOC
COOEt
COOEt
N
O
Boc
3r
f1 (ppm)

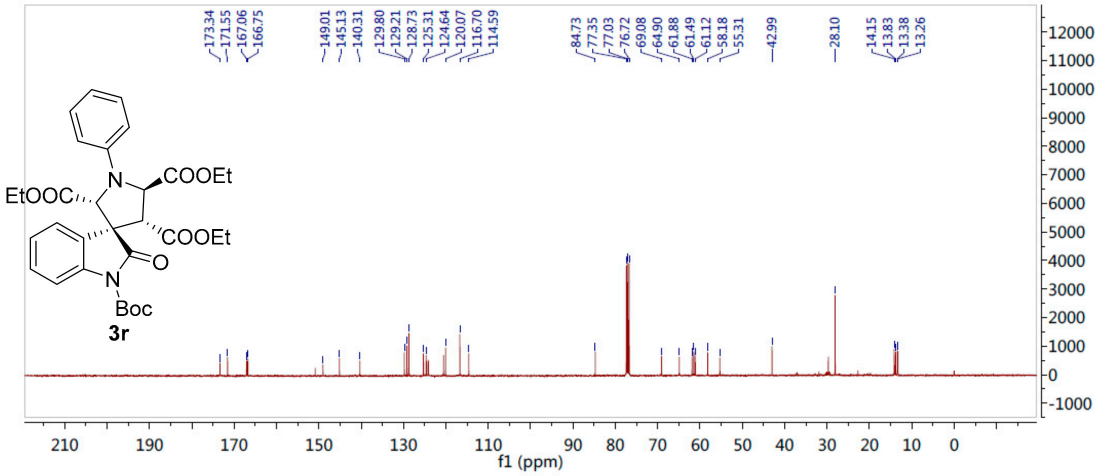
173.34
171.55
167.06
166.75
149.01
145.13
140.31
129.80
129.21
128.73
125.31
124.64
120.07
116.70
114.59
84.73
77.35
77.03
76.72
69.08
64.90
61.88
61.49
61.12
58.18
55.31
42.99
28.10
14.15
13.83
13.38
13.26
EtOOC
COOEt
COOEt
N
N
O
Boc
3r
f1 (ppm)

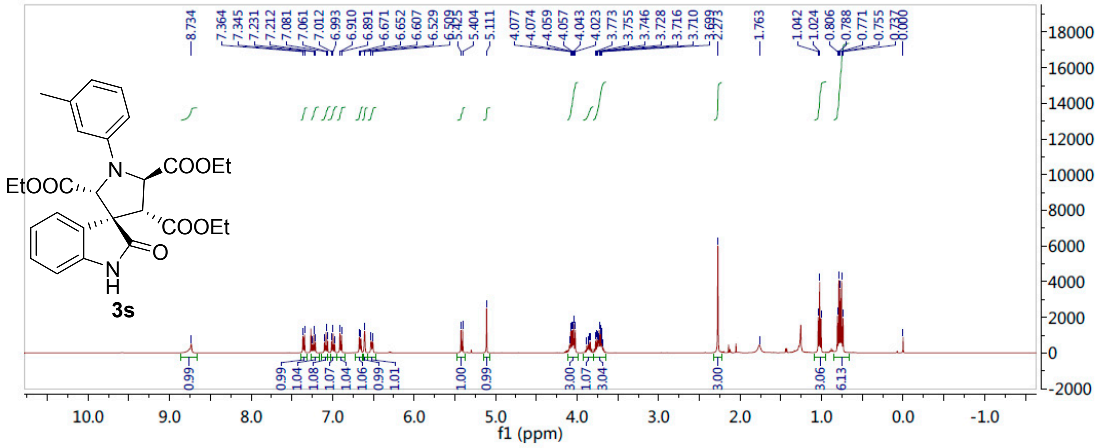
8.734
7.364
7.345
7.231
7.212
7.081
7.061
7.012
6.993
6.910
6.891
6.671
6.652
6.607
6.529
5.404
5.111
4.077
4.074
4.059
4.057
4.043
4.023
3.773
3.755
3.746
3.728
3.716
3.710
2.273
1.763
1.042
1.024
0.806
0.788
0.771
0.755
0.000
0.99
0.99
1.04
1.08
1.07
1.04
1.06
0.99
1.01
1.00
0.99
3.00
1.07
3.04
3.00
3.06
6.13
EtOOC
COOEt
COOEt
N
N
H
O
3s
f1 (ppm)

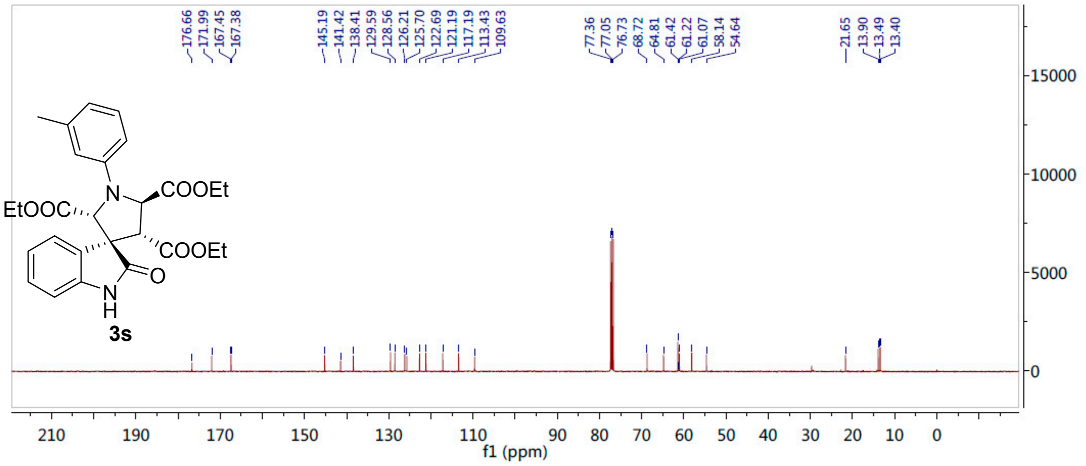
176.66
171.99
167.45
167.38
145.19
141.42
138.41
129.59
128.56
126.21
125.70
122.69
121.19
117.19
113.43
109.63
77.36
77.05
76.73
68.72
64.81
61.42
61.22
61.07
58.14
54.64
21.65
13.90
13.49
13.40
EtOOC
COOEt
COOEt
N
N
H
O
3s
f1 (ppm)

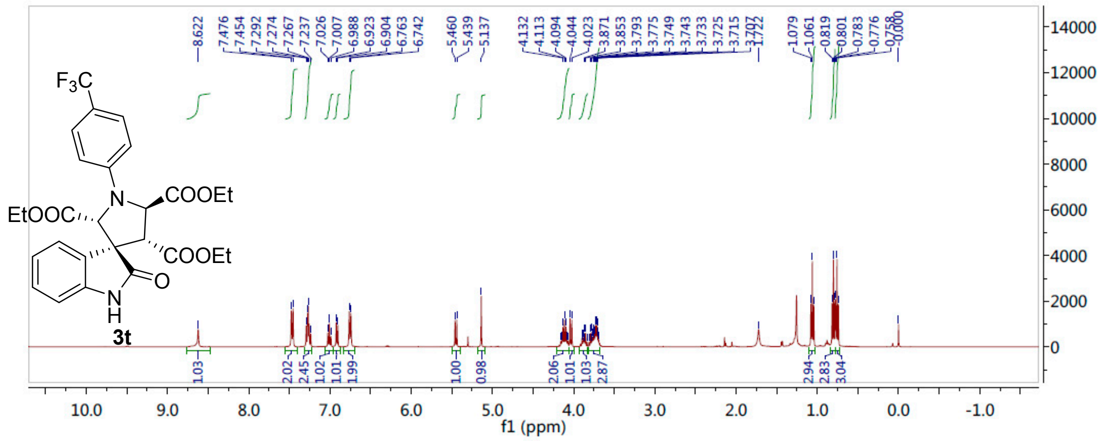
F3C
EtOOC
N
COOEt
COOEt
O
NH
3t
f1 (ppm)

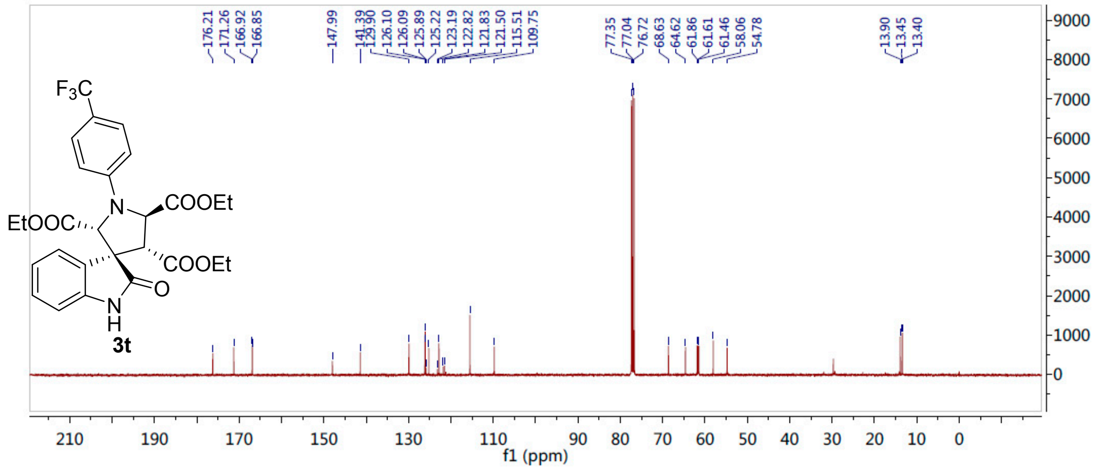
F3C
EtOOC
N
COOEt
COOEt
O
NH
3t
f1 (ppm)

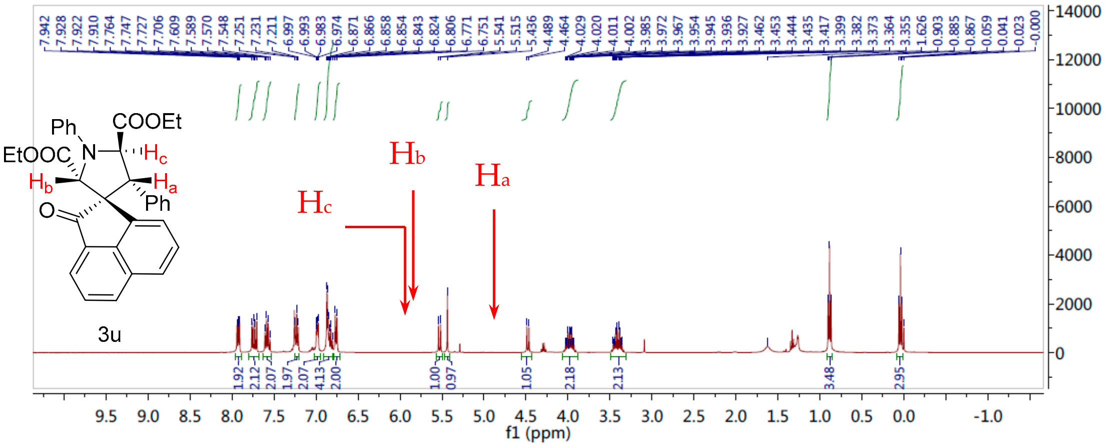
Ph
COOEt
EtOOC
N
Hc
Hb
Ha
Ph
O
3u
Hc
Hb
Ha
f1 (ppm)

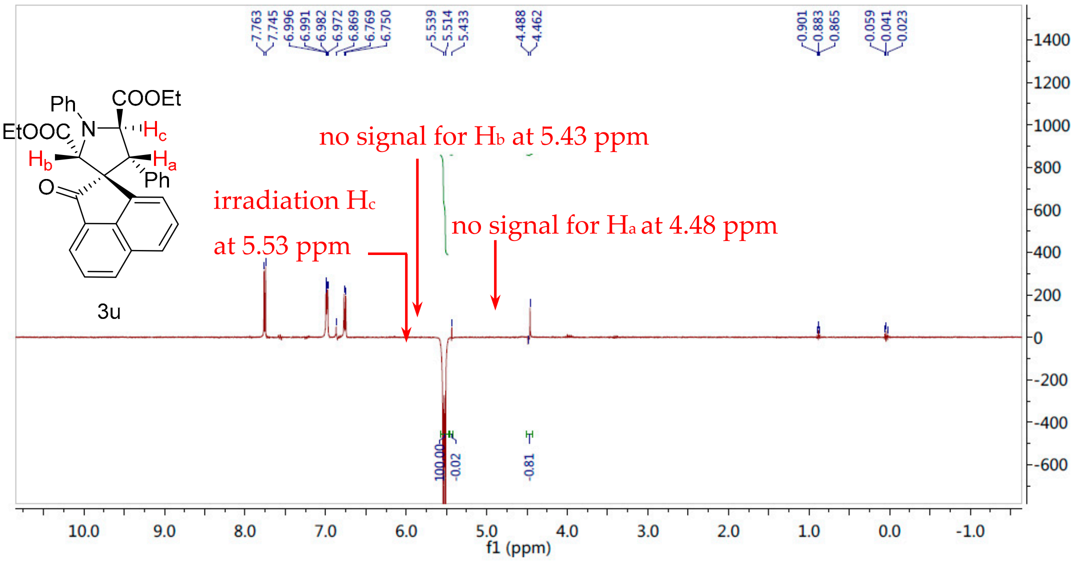

7.763 7.745 6.996 6.991 6.982 6.972 6.869 6.769 6.750 5.539 5.514 5.433 4.488 4.462 0.901 0.883 0.865 0.059 0.041 0.023
Ph
COOEt
EtOOC
N
Hc
Hb
Ha
Ph
O
3u
no signal for Hb at 5.43 ppm
irradiation Hc at 5.53 ppm
no signal for Ha at 4.48 ppm
100.00
-0.02
-0.81
f1 (ppm)

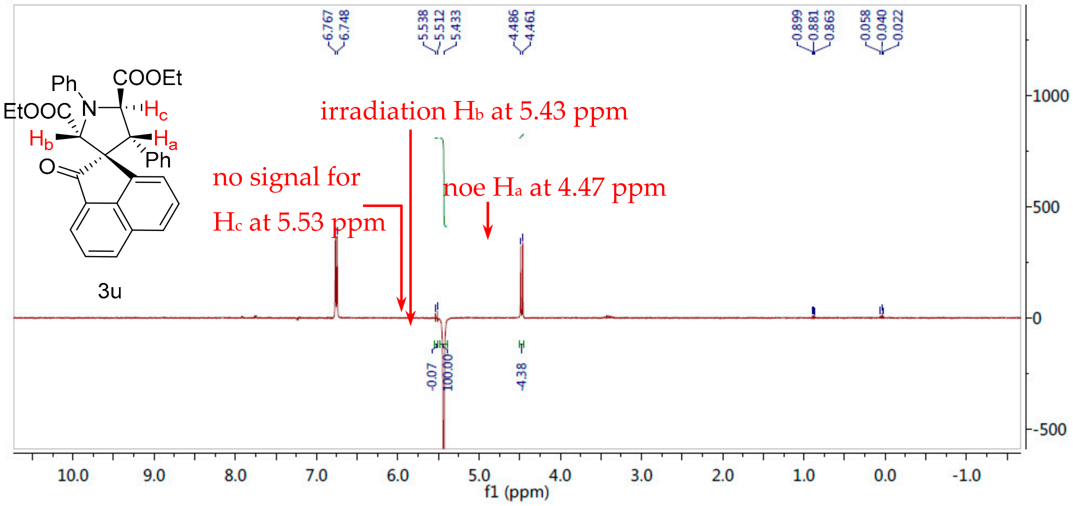

6.767 6.748 5.538 5.512 5.433 4.486 4.461 0.899 0.881 0.863 0.058 0.040 0.022
Ph
COOEt
EtOOC
N
Hc
Hb
Ha
Ph
O
3u
irradiation Hb at 5.43 ppm
no signal for Hc at 5.53 ppm
noe Ha at 4.47 ppm
-0.07
100.00
-4.38
f1 (ppm)

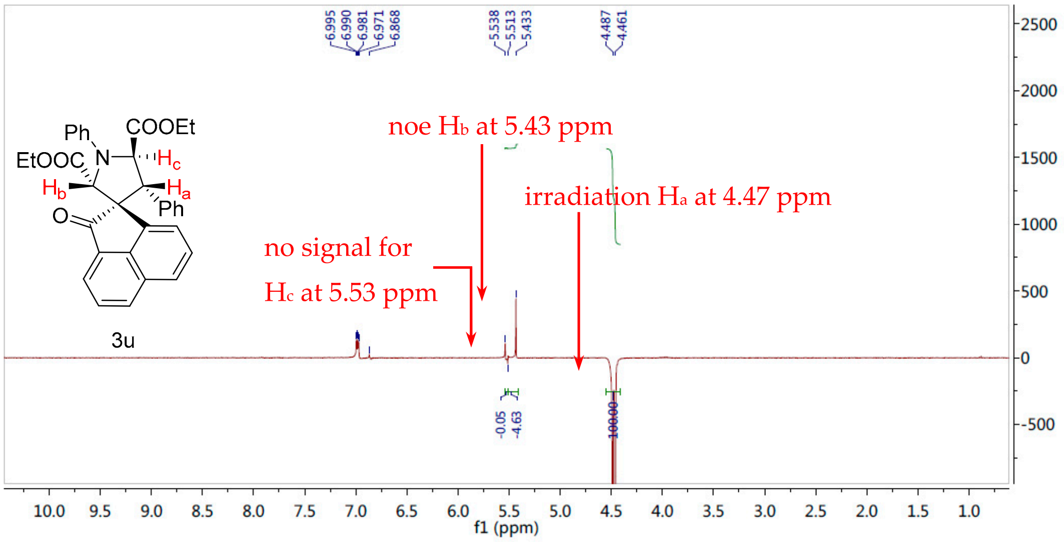

6.995 6.990 6.981 6.971 6.868 5.538 5.513 5.433 4.487 4.461
Ph
COOEt
EtOOC
N
Hc
Hb
Ha
Ph
O
3u
noe Hb at 5.43 ppm
irradiation Ha at 4.47 ppm
no signal for Hc at 5.53 ppm
-0.05
-4.63
100.00
f1 (ppm)

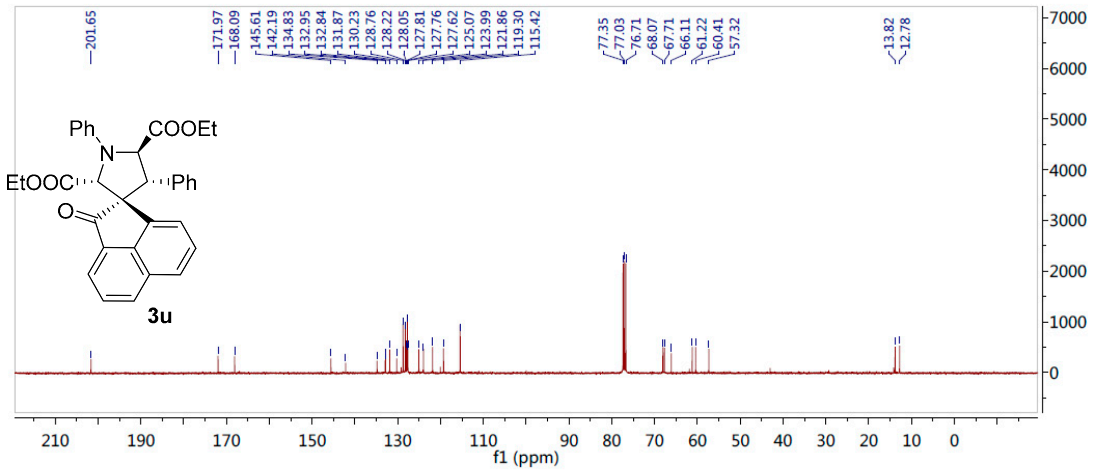
201.65
171.97
168.09
145.61
142.19
134.83
132.95
132.84
131.87
130.23
128.76
128.22
128.05
127.81
127.76
127.62
125.07
123.99
121.86
119.30
115.42
77.35
77.03
76.71
68.07
67.71
66.11
61.22
60.41
57.32
13.82
12.78
Ph
N
COOEt
EtOOC
Ph
O
3u
f1 (ppm)

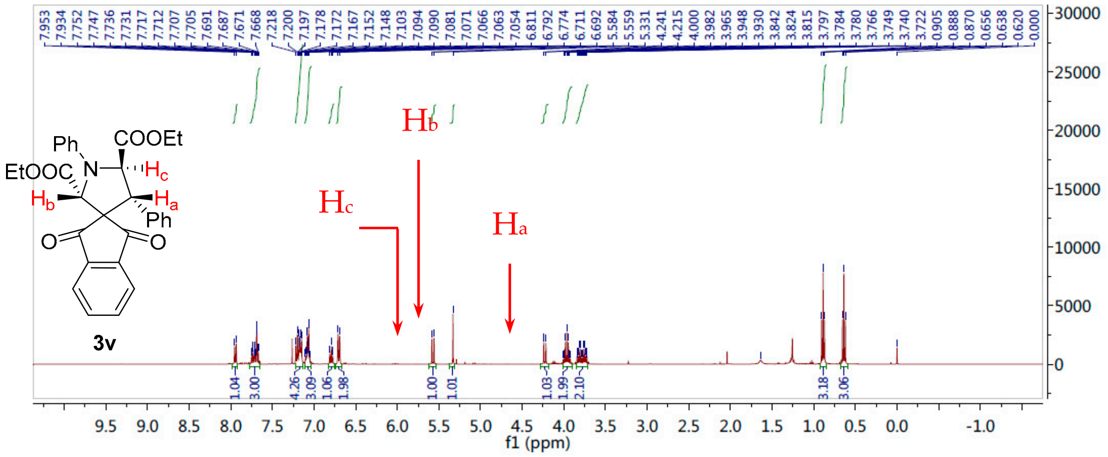
7.953
7.934
7.752
7.747
7.736
7.731
7.717
7.712
7.707
7.705
7.691
7.687
7.671
7.668
7.218
7.200
7.197
7.178
7.172
7.167
7.152
7.148
7.103
7.094
7.090
7.081
7.071
7.066
7.063
7.054
6.811
6.792
6.774
6.711
6.692
5.584
5.559
5.331
4.241
4.215
4.000
3.982
3.965
3.948
3.930
3.842
3.824
3.815
3.797
3.784
3.780
3.766
3.749
3.740
3.722
0.905
0.888
0.870
0.656
0.638
0.620
0.000
Ph
COOEt
EtOOC
N
Hc
Hb
Ha
Ph
O
O
3v
Hb
Hc
Ha
1.04
3.00
4.26
3.09
1.06
1.98
1.00
1.01
1.03
1.99
2.10
3.18
3.06
f1 (ppm)

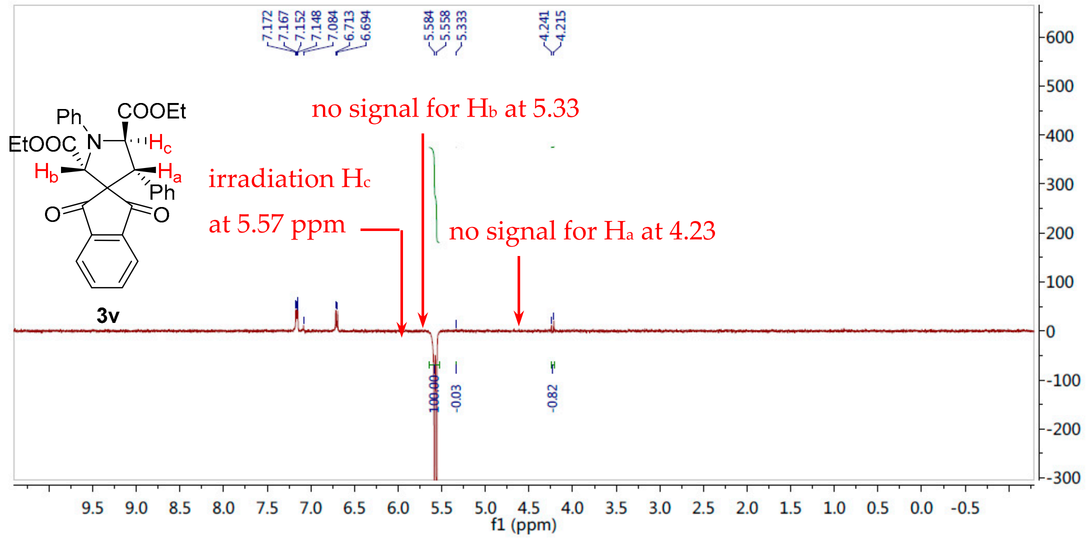
7.172
7.167
7.152
7.148
7.084
6.713
6.694
5.584
5.558
5.333
4.241
4.215
Ph
COOEt
EtOOC
N
Hc
Hb
Ha
Ph
O
O
3v
no signal for Hb at 5.33
irradiation Hc
at 5.57 ppm
no signal for Ha at 4.23
100.00
-0.03
-0.82
f1 (ppm)

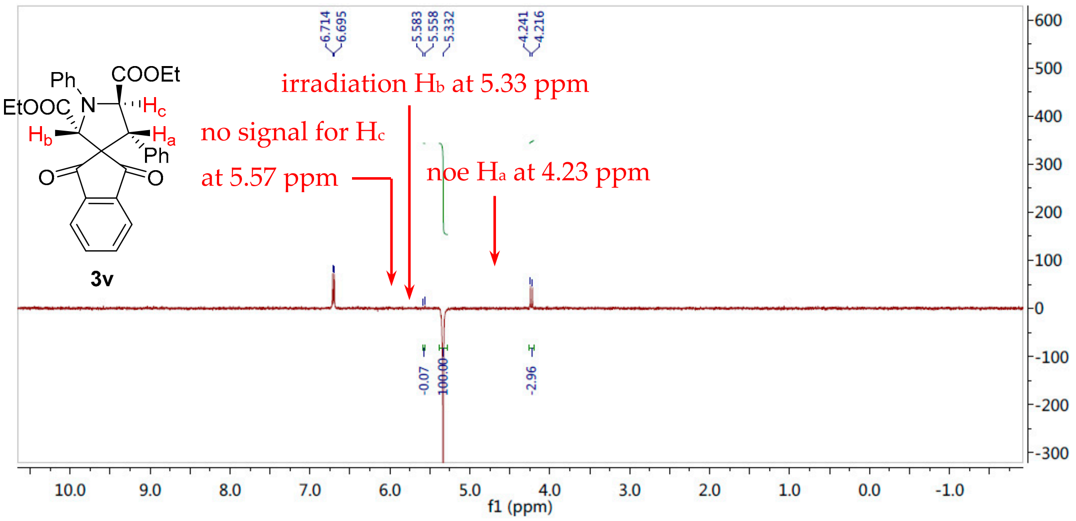
6.714
6.695
5.583
5.558
5.332
4.241
4.216
irradiation Hb at 5.33 ppm
no signal for Hc
at 5.57 ppm
noe Ha at 4.23 ppm
Ph
COOEt
EtOOC
N
Hc
Hb
Ha
Ph
O
O
3v
-0.07
100.00
-2.96
f1 (ppm)

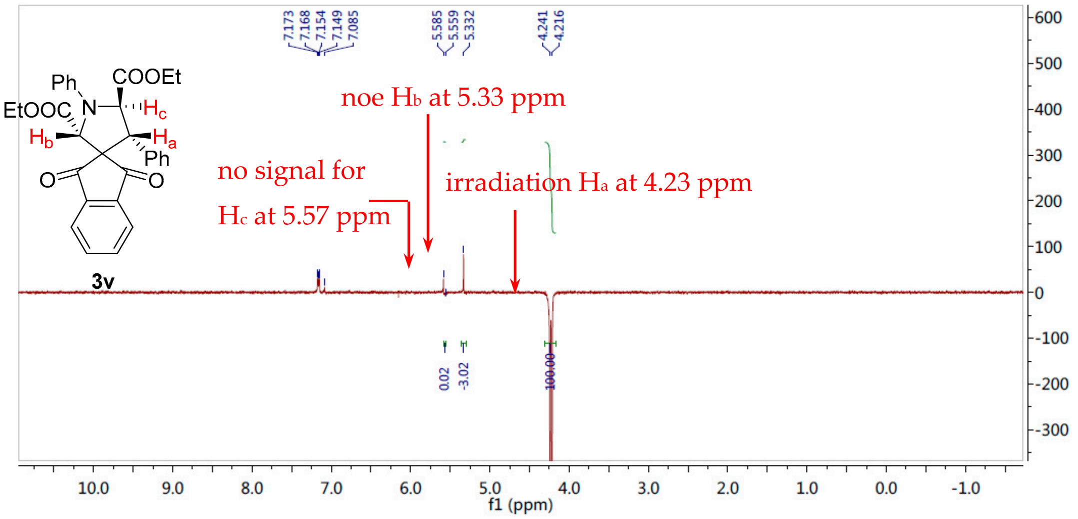
7.173
7.168
7.154
7.149
7.085
5.585
5.559
5.332
4.241
4.216
noe Hb at 5.33 ppm
no signal for
Hc at 5.57 ppm
irradiation Ha at 4.23 ppm
Ph
COOEt
EtOOC
N
Hc
Hb
Ha
Ph
O
O
3v
0.02
-3.02
100.00
f1 (ppm)

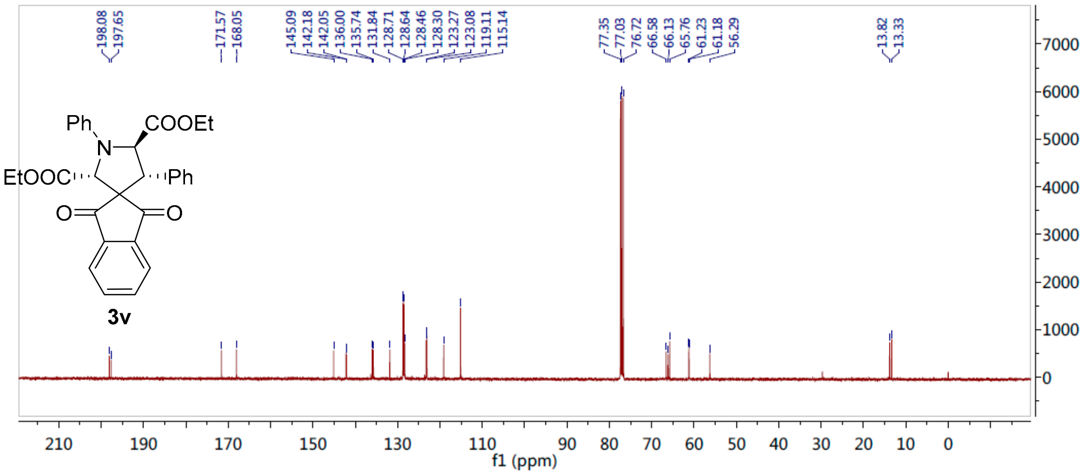
198.08
197.65
171.57
168.05
145.09
142.18
142.05
136.00
135.74
131.84
128.71
128.64
128.46
128.30
123.27
123.08
119.11
115.14
77.35
77.03
76.72
66.58
66.13
65.76
61.23
61.18
56.29
13.82
13.33
Ph
COOEt
N
EtOOC
Ph
O
O
3v
f1 (ppm)

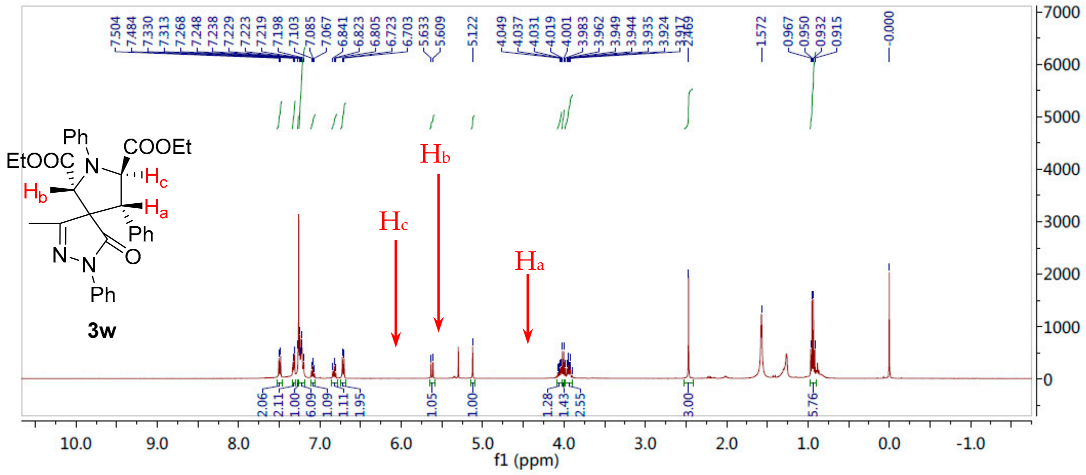
Hc
Hb
Ha
3w
f1 (ppm)

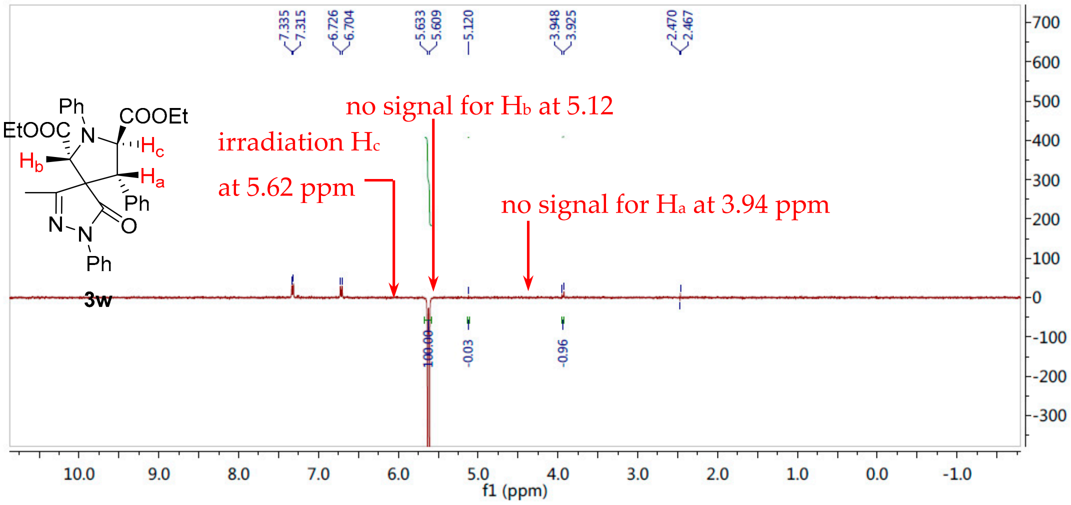
no signal for Hb at 5.12
irradiation Hc
at 5.62 ppm
no signal for Ha at 3.94 ppm
3w
f1 (ppm)

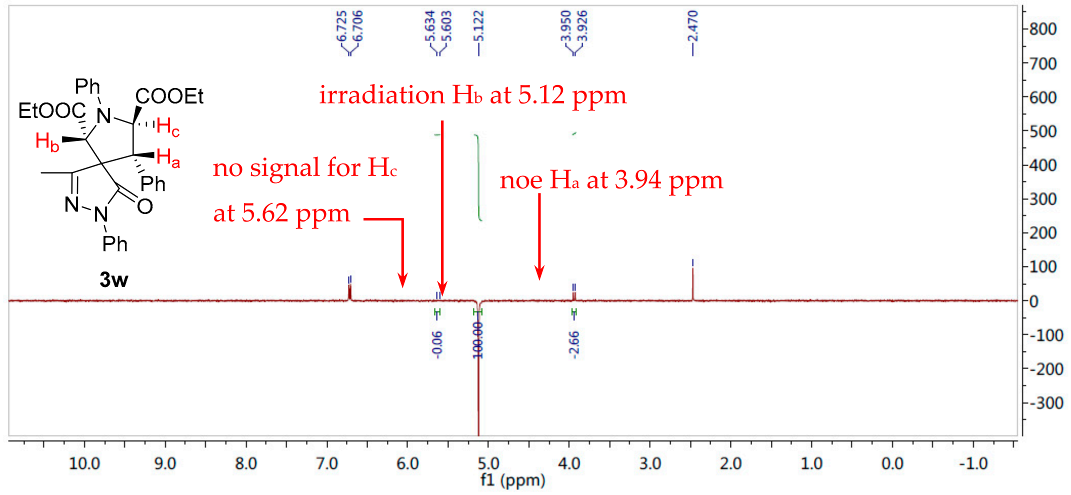
irradiation Hb at 5.12 ppm
no signal for Hc
at 5.62 ppm
noe Ha at 3.94 ppm
3w
f1 (ppm)

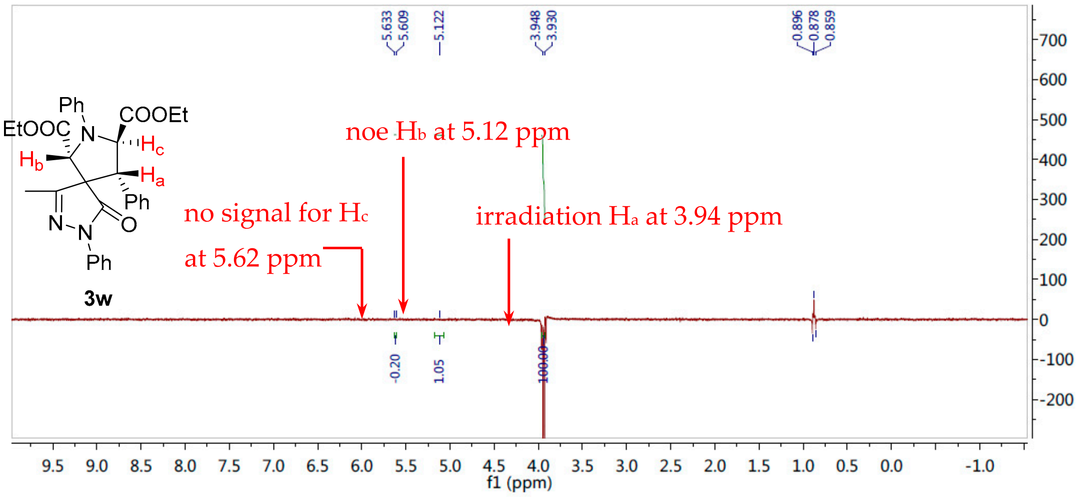

Ph
COOEt
EtOOC
N
Hc
Hb
Ha
Ph
N
N
O
Ph
3w
noe Hb at 5.12 ppm
no signal for Hc
at 5.62 ppm
irradiation Ha at 3.94 ppm
f1 (ppm)

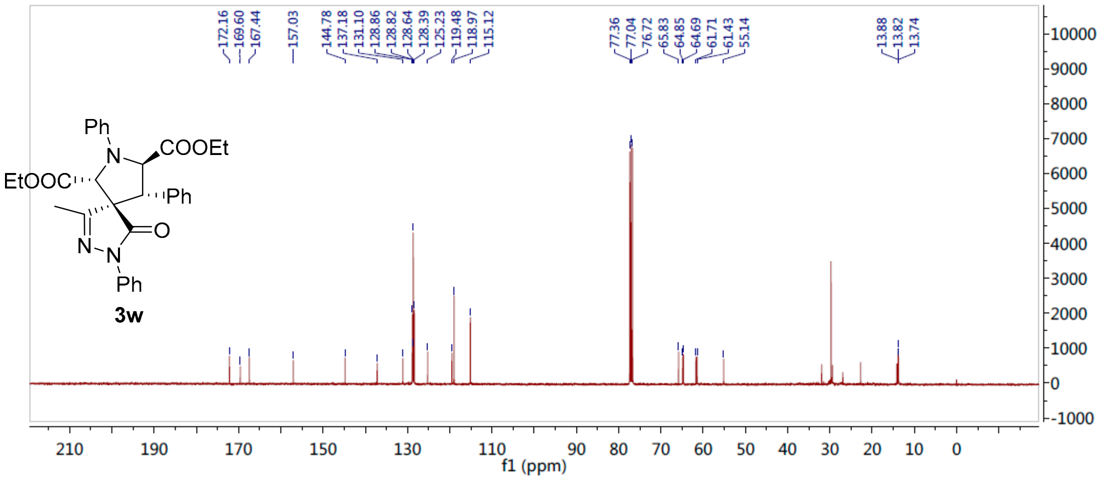

Ph
N
COOEt
EtOOC
Ph
O
N
N
Ph
3w
f1 (ppm)

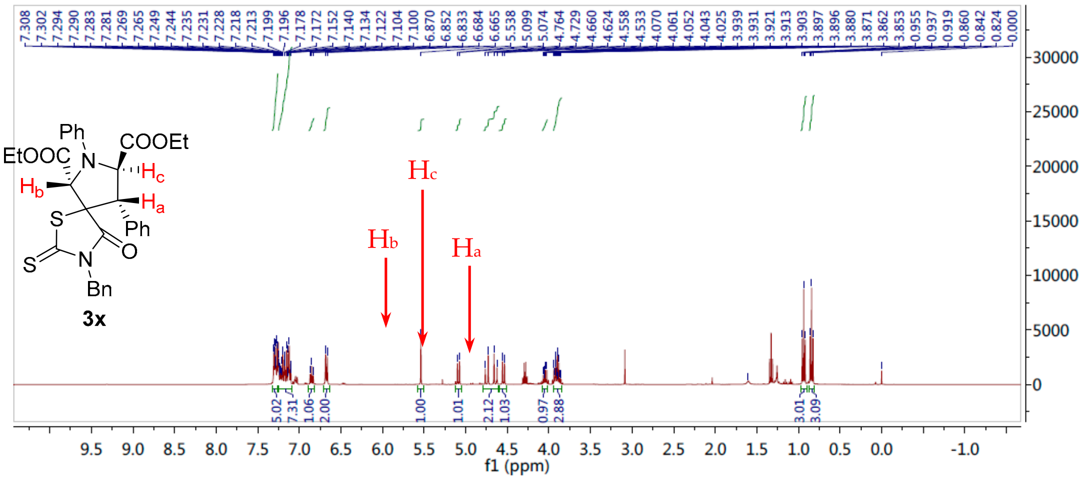

Ph
COOEt
EtOOC
N
Hc
Hb
Ha
Ph
S
S
N
O
Bn
3x
Hc
Hb
Ha
f1 (ppm)

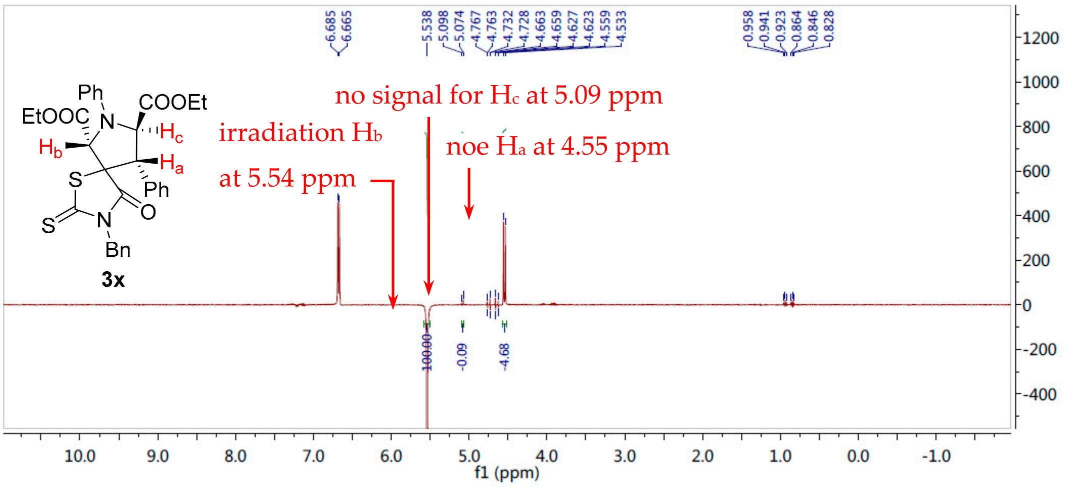
no signal for Hc at 5.09 ppm
irradiation Hb
at 5.54 ppm
noe Ha at 4.55 ppm
3x
f1 (ppm)

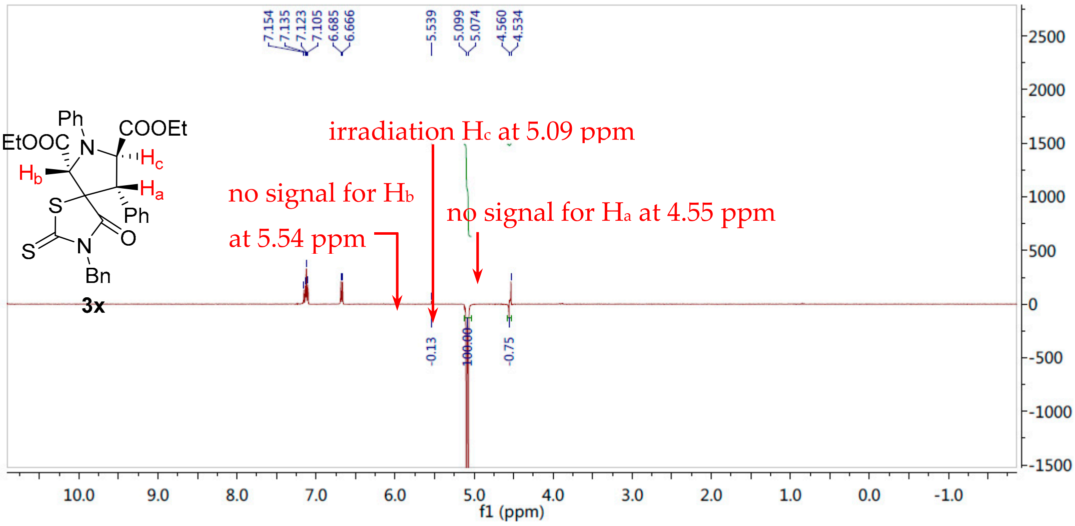
irradiation Hc at 5.09 ppm
no signal for Hb
at 5.54 ppm
no signal for Ha at 4.55 ppm
3x
f1 (ppm)

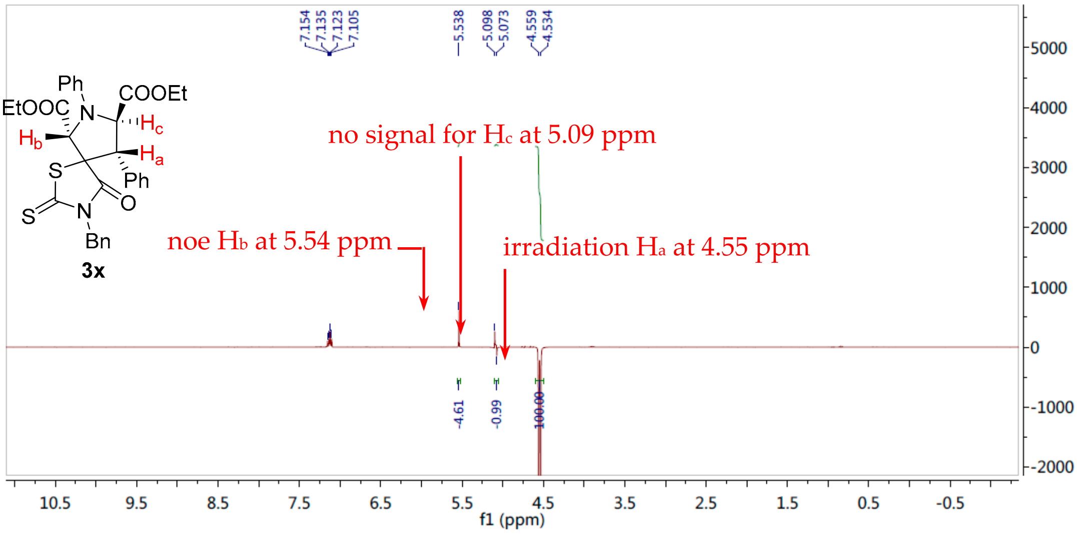
no signal for Hc at 5.09 ppm
noe Hb at 5.54 ppm
irradiation Ha at 4.55 ppm
3x
f1 (ppm)

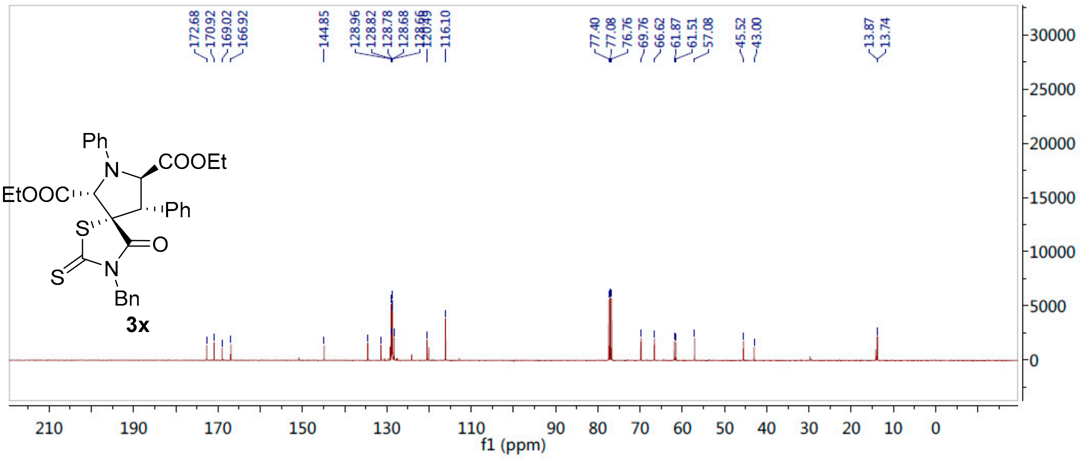

6. HRMS Spectra and Elemental Analysis Reports

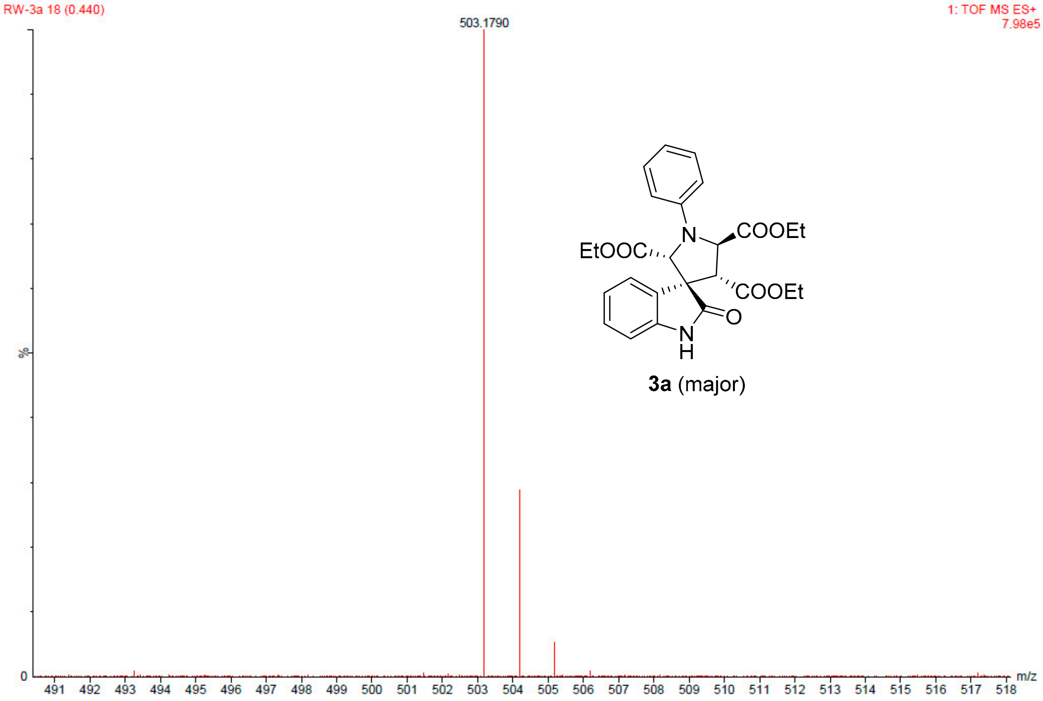

Elemental Composition Report Page 1

Single Mass Analysis
Tolerance = 2.0 mDa / DBE: min = -1.5, max = 50.0
Element prediction: Off
Number of isotope peaks used for i-FIT = 3

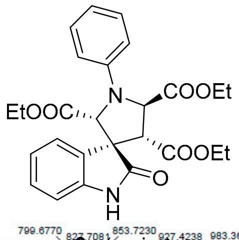

Monoisotopic Mass, Even Electron Ions
1550 formula(e) evaluated with 8 results within limits (up to 50 closest results for each mass)
Elements Used:
C: 20-34 H: 0-40 N: 2-4 O: 4-8 F: 0-1 Na: 0-1 S: 0-2 Cl: 0-1 Br: 0-1

RW-3a 16 (0.406)
1: TOF MS ES+

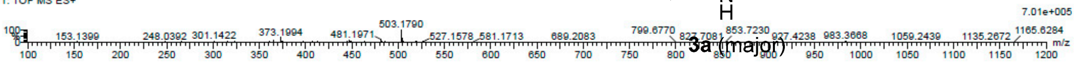

	Calc. Mass	mDa	PPM	DBE	i-FIT	Norm	Conf(%)	Formula
Minimum:				-1.5				
Maximum:		2.0	10.0	50.0				

Mass	Calc. Mass	mDa	PPM	DBE	i-FIT	Norm	Conf(%)	Formula
503.1790	503.1787	0.3	0.6	11.5	565.3	22.169	0.00	C24 H31 N4 O4 S2
	503.1794	-0.4	-0.8	13.5	543.2	0.000	99.98	C26 H28 N2 O7 Na
	503.1783	0.7	1.4	7.5	570.3	27.160	0.00	C23 H33 N2 O5 F S Cl
	503.1798	-0.8	-1.6	7.5	565.3	22.134	0.00	C21 H32 N4 O5 F S2
	503.1781	0.9	1.8	3.5	570.7	27.510	0.00	C21 H37 N2 O4 Na S2 Cl
	503.1805	-1.5	-3.0	6.5	570.5	27.293	0.00	C23 H36 N2 O4 S2 Cl
	503.1806	-1.6	-3.2	9.5	551.8	8.601	0.02	C23 H29 N2 O8 F Na
	503.1771	1.9	3.8	11.5	570.4	27.190	0.00	C26 H32 N2 O4 S Cl

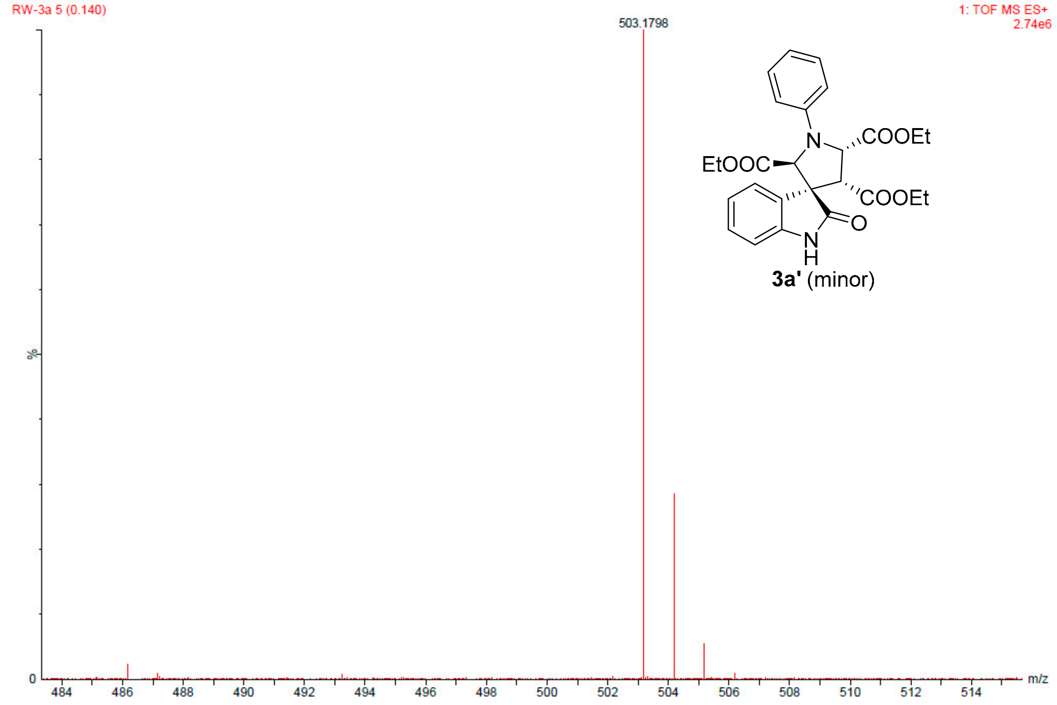

3a' (minor)

Elemental Composition Report **Page 1**

Single Mass Analysis
Tolerance = 2.0 mDa / DBE: min = -1.5, max = 50.0
Element prediction: Off
Number of isotope peaks used for i-FIT = 3

Monoisotopic Mass, Even Electron Ions
2438 formula(e) evaluated with 10 results within limits (up to 50 closest results for each mass)
Elements Used:
C: 0-34 H: 0-40 N: 2-4 O: 4-8 F: 0-1 Na: 0-1 S: 0-2 Cl: 0-1 Br: 0-1

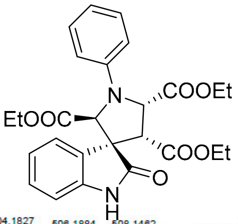

3a' (minor)

RW-3a 5 (0.140)
1: TOF MS ES+

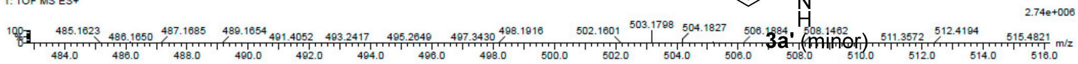

Minimum:				-1.5				
Maximum:		2.0	10.0	50.0				
Mass	Calc. Mass	mDa	PPM	DBE	i-FIT	Norm	Conf(%)	Formula
503.1798	503.1798	0.0	0.0	7.5	708.7	20.942	0.00	C21 H32 N4 O5 F S2
	503.1794	0.4	0.8	13.5	687.8	0.009	99.13	C26 H28 N2 O7 Na
	503.1792	0.6	1.2	-0.5	714.1	26.253	0.00	C18 H38 N2 O5 F Na S2 Cl
	503.1805	-0.7	-1.4	6.5	713.8	26.026	0.00	C23 H36 N2 O4 S2 Cl
	503.1806	-0.8	-1.6	9.5	692.5	4.739	0.87	C23 H29 N2 O8 F Na
	503.1790	0.8	1.6	0.5	715.0	27.150	0.00	C19 H40 N2 O6 S Br
	503.1787	1.1	2.2	11.5	708.8	21.010	0.00	C24 H31 N4 O4 S2
	503.1783	1.5	3.0	7.5	713.7	25.943	0.00	C23 H33 N2 O5 F S Cl
	503.1781	1.7	3.4	3.5	714.1	26.297	0.00	C21 H37 N2 O4 Na S2 Cl
	503.1816	-1.8	-3.6	2.5	713.9	26.051	0.00	C20 H37 N2 O5 F S2 Cl

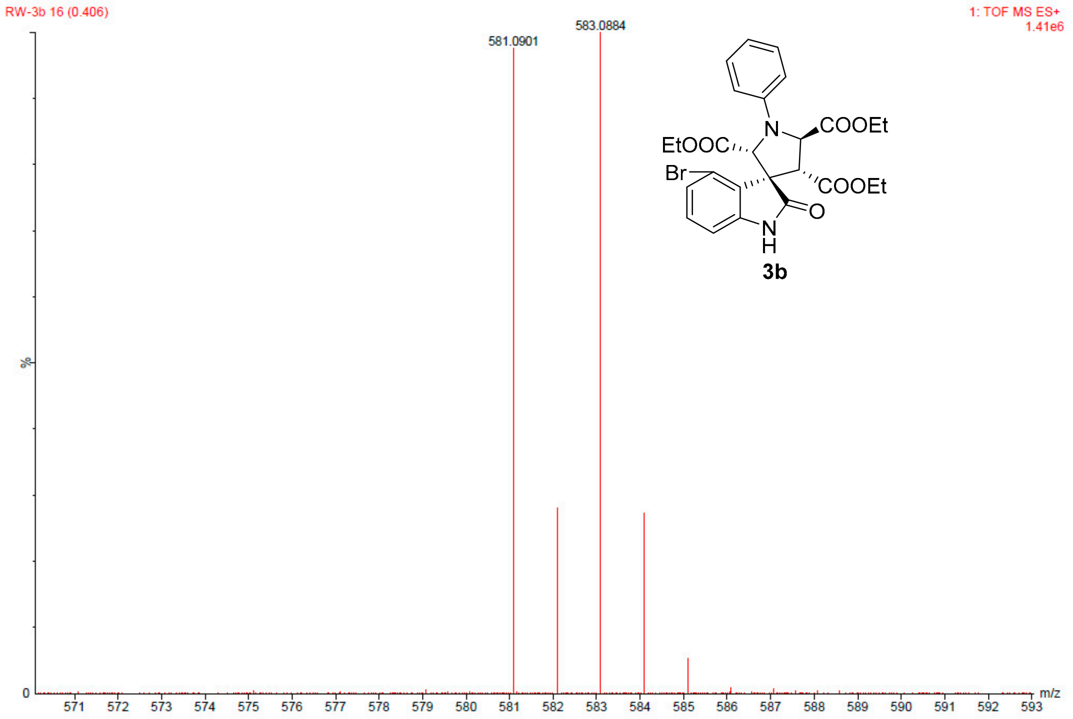

Elemental Composition Report **Page 1**

Single Mass Analysis
Tolerance = 2.0 mDa / DBE: min = -1.5, max = 50.0
Element prediction: Off
Number of isotope peaks used for i-FIT = 3

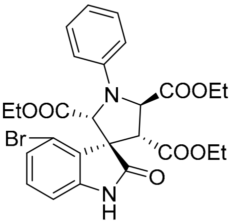

Monoisotopic Mass, Even Electron Ions
1109 formula(e) evaluated with 10 results within limits (up to 50 closest results for each mass)
Elements Used:
C: 0-36 H: 0-36 N: 2-4 O: 4-8 F: 0-1 Na: 0-1 S: 0-2 Br: 0-1

RW-3b 16 (0.406)
1: TOF MS ES+

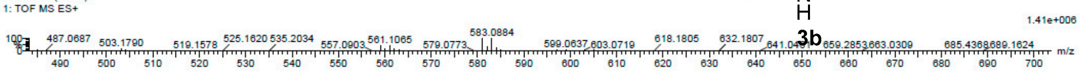

Mass	Calc. Mass	mDa	PPM	DBE	i-FIT	Norm	Conf(%)	Formula
Minimum:				-1.5				
Maximum:		2.0	10.0	50.0				
581.0901	581.0903	-0.2	-0.3	7.5	537.1	8.064	0.03	C21 H31 N4 O5 F S2 Br
	581.0899	0.2	0.3	13.5	529.0	0.002	99.75	C26 H27 N2 O7 Na Br
	581.0897	0.4	0.7	28.5	556.1	27.147	0.00	C33 H14 N4 O6 F
	581.0896	0.5	0.9	24.5	553.1	24.110	0.00	C31 H18 N4 O5 Na S
	581.0907	-0.6	-1.0	20.5	552.9	23.941	0.00	C28 H19 N4 O6 F Na S
	581.0892	0.9	1.5	11.5	536.9	7.901	0.04	C24 H30 N4 O4 S2 Br
	581.0911	-1.0	-1.7	9.5	535.3	6.338	0.18	C23 H28 N2 O8 F Na Br
	581.0914	-1.3	-2.2	29.5	556.5	27.496	0.00	C36 H15 N2 O4 F Na
	581.0886	1.5	2.6	32.5	556.1	27.100	0.00	C36 H13 N4 O5
	581.0920	-1.9	-3.3	27.5	554.0	24.968	0.00	C33 H17 N4 O5 S

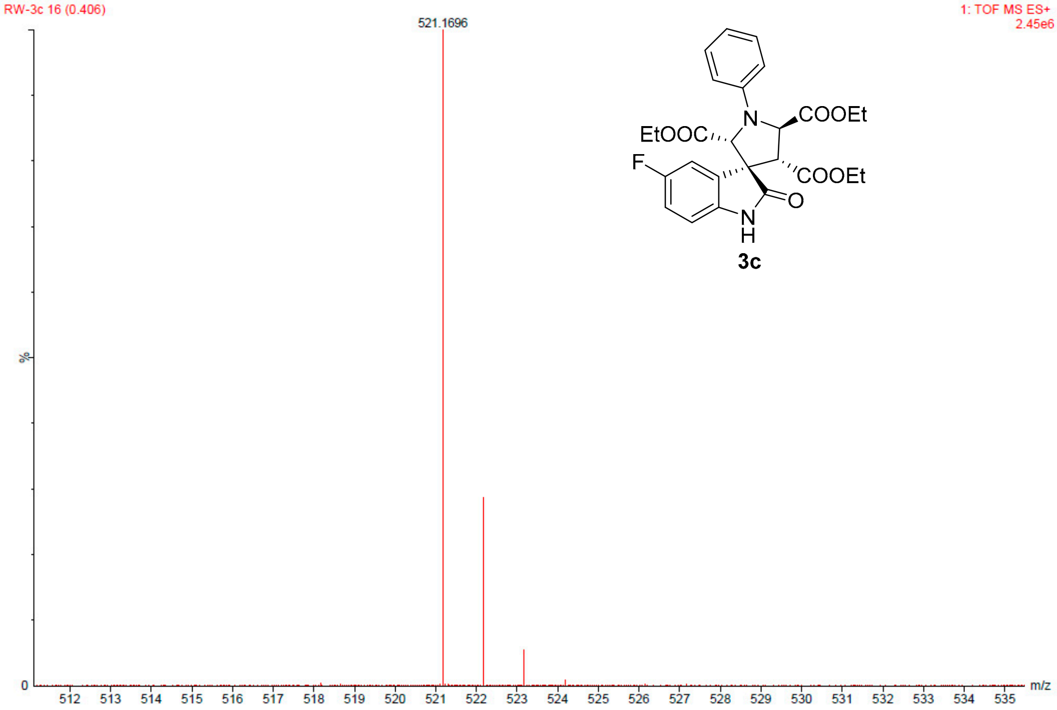

Elemental Composition Report Page 1

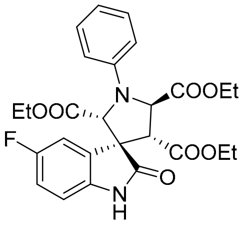

Single Mass Analysis
Tolerance = 2.0 mDa / DBE: min = -1.5, max = 50.0
Element prediction: Off
Number of isotope peaks used for i-FIT = 3

Monoisotopic Mass, Even Electron Ions
1137 formula(e) evaluated with 7 results within limits (up to 50 closest results for each mass)
Elements Used:
C: 0-36 H: 0-36 N: 2-4 O: 4-8 F: 0-1 Na: 0-1 S: 0-2 Br: 0-1

RW-3c 16 (0.406)
1: TOF MS ES+

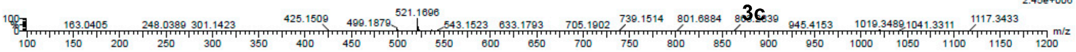

Minimum:				-1.5				
Maximum:		2.0	10.0	50.0				
Mass	Calc. Mass	mDa	PPM	DBE	i-FIT	Norm	Conf(%)	Formula
521.1696	521.1693	0.3	0.6	11.5	692.5	21.196	0.00	C24 H30 N4 O4 F S2
	521.1700	-0.4	-0.8	13.5	671.3	0.001	99.91	C26 H27 N2 O7 F Na
	521.1689	0.7	1.3	17.5	678.4	7.104	0.08	C29 H26 N2 O6 Na
	521.1706	-1.0	-1.9	11.5	688.9	17.605	0.00	C23 H29 N4 O8 S
	521.1682	1.4	2.7	8.5	689.3	18.000	0.00	C21 H30 N4 O8 Na S
	521.1713	-1.7	-3.3	20.5	681.0	9.667	0.01	C31 H25 N2 O6
	521.1716	-2.0	-3.8	3.5	692.6	21.326	0.00	C18 H34 N4 O8 Na S2

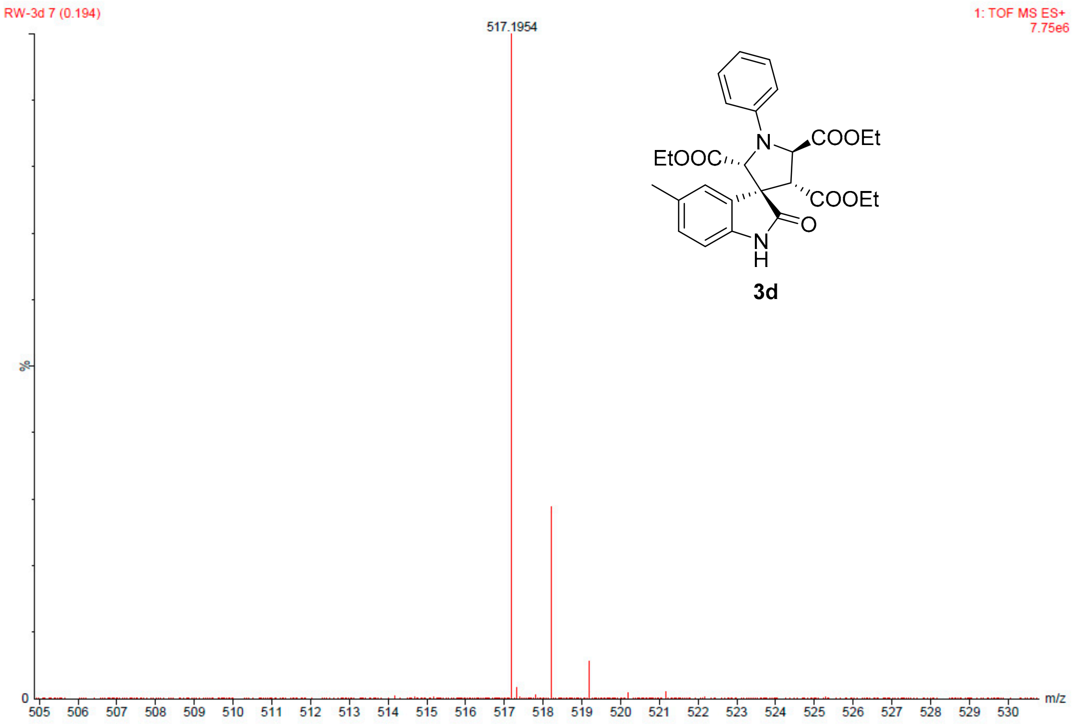

Elemental Composition Report **Page 1**

Single Mass Analysis
Tolerance = 2.0 mDa / DBE: min = -1.5, max = 50.0
Element prediction: Off
Number of isotope peaks used for i-FIT = 3

Monoisotopic Mass, Even Electron Ions
901 formula(e) evaluated with 4 results within limits (up to 50 closest results for each mass)
Elements Used:
C: 20-34 H: 0-40 N: 2-4 O: 4-8 Na: 0-1 S: 0-2 Cl: 0-1 Br: 0-1

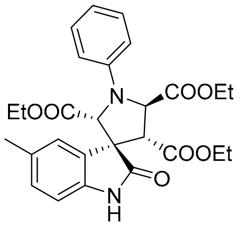

3d

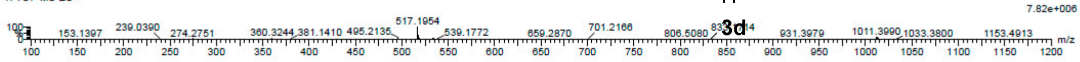

Mass	Calc. Mass	mDa	PPM	DBE	i-FIT	Norm	Conf(%)	Formula
Minimum:				-1.5				
Maximum:		2.0	10.0	50.0				
517.1954	517.1951	0.3	0.6	13.5	695.2	0.000	100.00	C27 H30 N2 O7 Na
	517.1962	-0.8	-1.5	6.5	718.2	22.963	0.00	C24 H38 N2 O4 S2 Cl
	517.1943	1.1	2.1	11.5	713.1	17.881	0.00	C25 H33 N4 O4 S2
	517.1937	1.7	3.3	3.5	718.5	23.269	0.00	C22 H39 N2 O4 Na S2 Cl

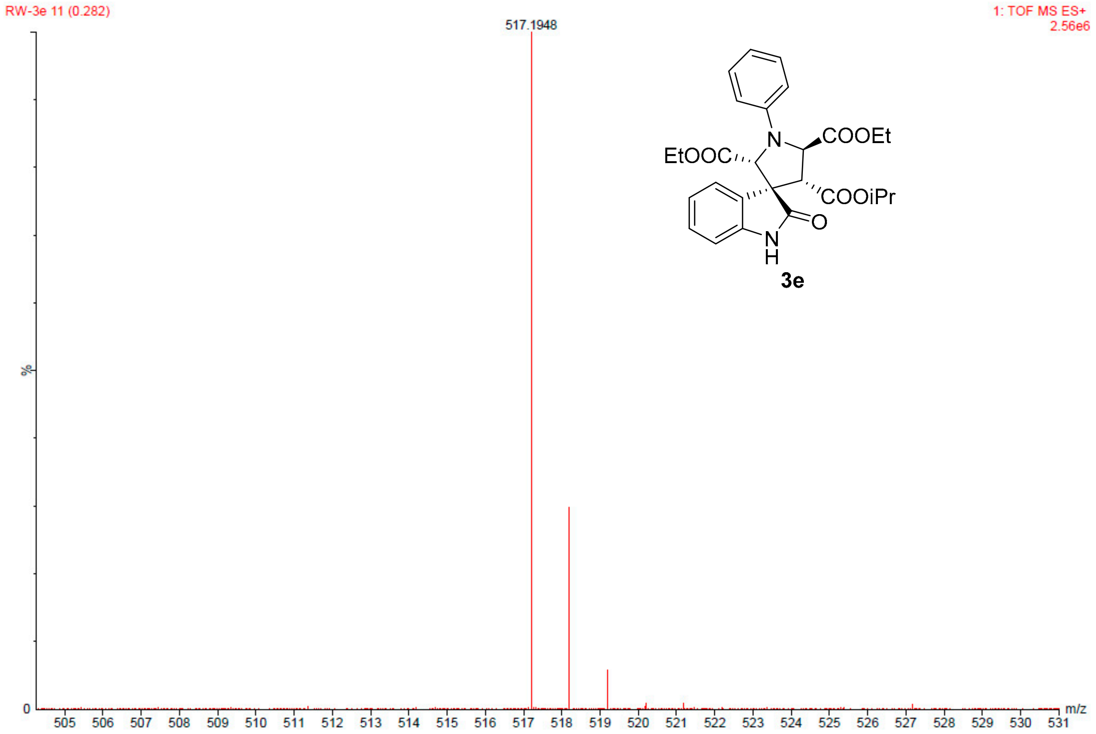

Elemental Composition Report

Page 1

Single Mass Analysis
Tolerance = 2.0 mDa / DBE: min = -1.5, max = 50.0
Element prediction: Off
Number of isotope peaks used for i-FIT = 3

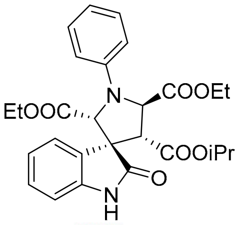

3e

Monoisotopic Mass, Even Electron Ions
1358 formula(e) evaluated with 8 results within limits (up to 50 closest results for each mass)
Elements Used:
C: 20-40 H: 20-40 N: 0-6 O: 5-8 F: 0-1 Na: 0-1 S: 0-4 Cl: 0-1
RW-3e 11 (0.282)
1: TOF MS ES+

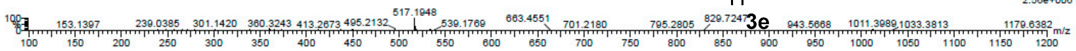

		mDa	PPM	DBE				
Minimum:				-1.5				
Maximum:		2.0	10.0	50.0				

Mass	Calc. Mass	mDa	PPM	DBE	i-FIT	Norm	Conf(%)	Formula
517.1948	517.1951	-0.3	-0.6	13.5	647.7	0.004	99.62	C27 H30 N2 O7 Na
	517.1942	0.6	1.2	9.5	673.5	25.782	0.00	C22 H31 N6 O5 Na Cl
	517.1955	-0.7	-1.4	7.5	668.8	21.073	0.00	C22 H34 N4 O5 F S2
	517.1939	0.9	1.7	7.5	673.9	26.190	0.00	C24 H35 N2 O5 F S Cl
	517.1962	-1.4	-2.7	9.5	653.3	5.568	0.38	C24 H31 N2 O8 F Na
	517.1931	1.7	3.3	4.5	669.2	21.486	0.00	C20 H35 N4 O5 F Na S2
	517.1930	1.8	3.5	6.5	669.2	21.500	0.00	C24 H37 O8 S2
	517.1966	-1.8	-3.5	12.5	673.2	25.495	0.00	C24 H30 N6 O5 Cl

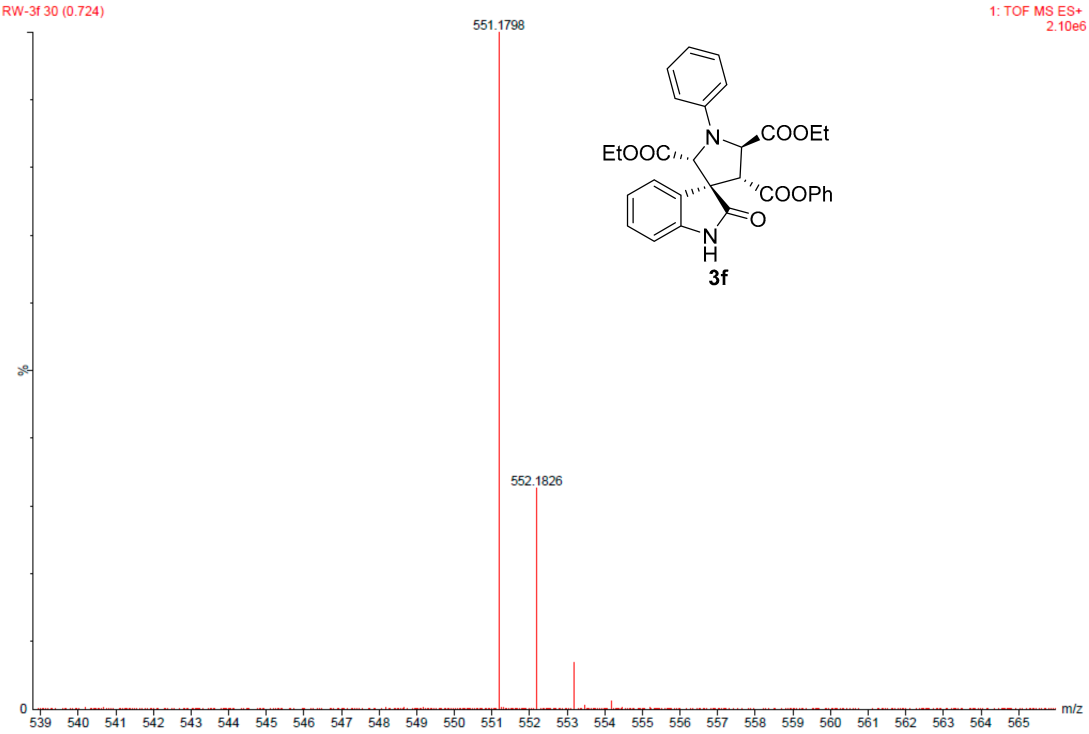

Elemental Composition Report **Page 1**

Single Mass Analysis
Tolerance = 2.0 mDa / DBE: min = -1.5, max = 50.0
Element prediction: Off
Number of isotope peaks used for i-FIT = 3

Monoisotopic Mass, Even Electron Ions
1576 formula(e) evaluated with 14 results within limits (up to 50 closest results for each mass)
Elements Used:
C: 20-40 H: 20-40 N: 0-6 O: 5-8 F: 0-1 Na: 0-1 S: 0-4 Cl: 0-1

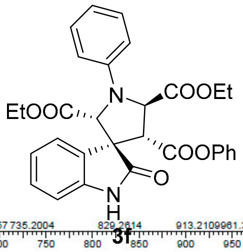

RW-3f 30 (0.724)
1: TOF MS ES+

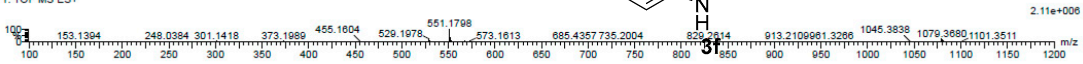

Mass	Calc. Mass	mDa	PPM	DBE	i-FIT	Norm	Conf(%)	Formula
Minimum:				-1.5				
Maximum:		2.0	10.0	50.0				
551.1798	551.1798	0.0	0.0	11.5	570.6	19.618	0.00	C25 H32 N4 O5 F S2
	551.1797	0.1	0.2	9.5	575.7	24.711	0.00	C22 H30 N6 O6 F Na Cl
	551.1794	0.4	0.7	17.5	551.0	0.013	98.75	C30 H28 N2 O7 Na
	551.1792	0.6	1.1	3.5	576.5	25.544	0.00	C22 H38 N2 O5 F Na S2 Cl
	551.1806	-0.8	-1.5	13.5	555.3	4.380	1.25	C27 H29 N2 O8 F Na
	551.1807	-0.9	-1.6	5.5	572.7	21.760	0.00	C24 H39 O8 S3
	551.1808	-1.0	-1.8	3.5	572.8	21.825	0.00	C20 H37 N4 O5 F Na S3
	551.1810	-1.2	-2.2	16.5	575.4	24.421	0.00	C27 H28 N6 O5 Cl
	551.1786	1.2	2.2	13.5	575.7	24.760	0.00	C25 H29 N6 O5 Na Cl
	551.1783	1.5	2.7	2.5	573.1	22.135	0.00	C22 H40 O8 Na S3
	551.1783	1.5	2.7	11.5	576.2	25.194	0.00	C27 H33 N2 O5 F S Cl
	551.1813	-1.5	-2.7	12.5	575.5	24.483	0.00	C29 H33 O7 Na Cl
	551.1780	1.8	3.3	6.5	573.1	22.151	0.00	C20 H35 N6 O6 S3
	551.1816	-1.8	-3.3	6.5	576.3	25.316	0.00	C24 H37 N2 O5 F S2 Cl

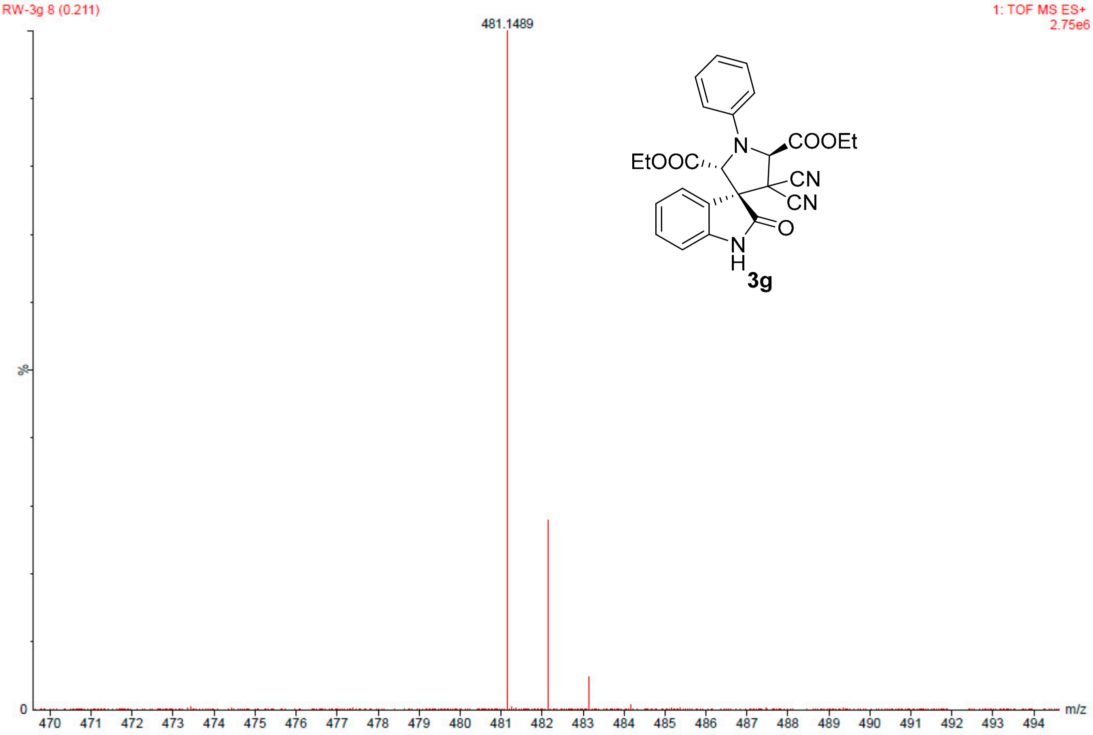

Elemental Composition Report **Page 1**

Single Mass Analysis
Tolerance = 2.0 mDa / DBE: min = -1.5, max = 50.0
Element prediction: Off
Number of isotope peaks used for i-FIT = 3

Monoisotopic Mass, Even Electron Ions
1130 formula(e) evaluated with 5 results within limits (up to 50 closest results for each mass)
Elements Used:
C: 0-36 H: 0-36 N: 2-4 O: 4-8 F: 0-1 Na: 0-1 S: 0-2 Br: 0-1

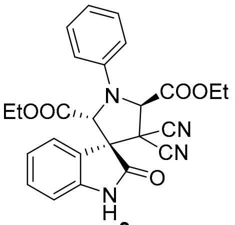

RW-3g 13 (0.335)
1: TOF MS ES+

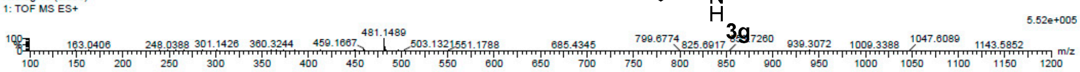

Minimum:				-1.5				
Maximum:		2.0	10.0	50.0				
Mass	Calc. Mass	mDa	PPM	DBE	i-FIT	Norm	Conf(%)	Formula
481.1489	481.1488	0.1	0.2	16.5	483.7	0.000	99.97	C25 H22 N4 O5 Na
	481.1484	0.5	1.0	3.5	511.9	28.216	0.00	C18 H34 N4 O4 S Br
	481.1496	-0.7	-1.5	-0.5	511.8	28.144	0.00	C15 H35 N4 O5 F S Br
	481.1499	-1.0	-2.1	12.5	491.7	8.048	0.03	C22 H23 N4 O6 F Na
	481.1478	1.1	2.3	5.5	506.2	22.477	0.00	C19 H30 N2 O7 F S2

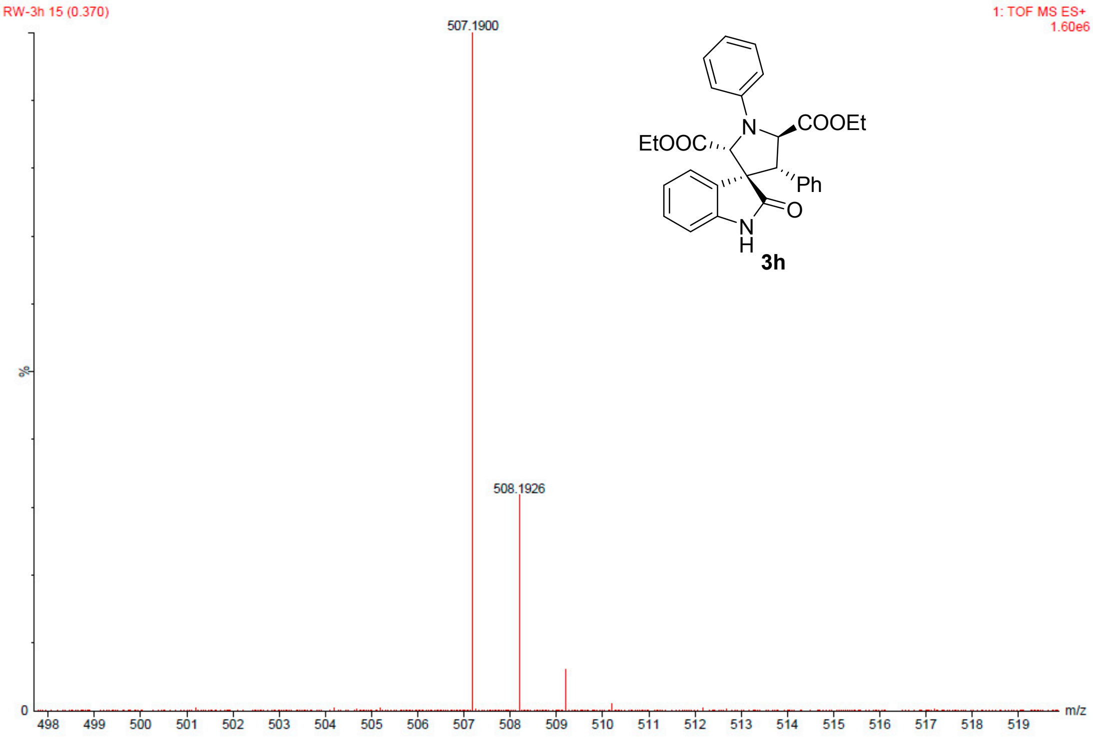

Elemental Composition Report Page 1

Single Mass Analysis
Tolerance = 2.0 mDa / DBE: min = -1.5, max = 50.0
Element prediction: Off
Number of isotope peaks used for i-FIT = 3

Monoisotopic Mass, Even Electron Ions
1315 formula(e) evaluated with 14 results within limits (up to 50 closest results for each mass)
Elements Used:
C: 20-40 H: 20-40 N: 0-6 O: 5-8 F: 0-1 Na: 0-1 S: 0-4 Cl: 0-1

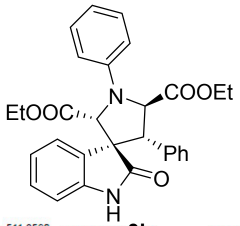

RW-3h 15 (0.370)
1: TOF MS ES+

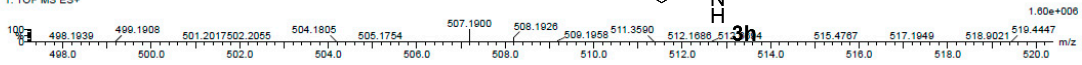

Mass	Calc. Mass	mDa	PPM	DBE	i-FIT	Norm	Conf(%)	Formula
Minimum:				-1.5				
Maximum:		2.0	10.0	50.0				
507.1900	507.1898	0.2	0.4	10.5	605.9	25.646	0.00	C25 H32 N2 O7 Cl
	507.1896	0.4	0.8	16.5	580.2	0.005	99.52	C29 H28 N2 O5 Na
	507.1907	-0.7	-1.4	12.5	585.7	5.450	0.43	C26 H29 N2 O6 F Na
	507.1908	-0.8	-1.6	2.5	606.3	26.105	0.00	C20 H37 N2 O7 Na S Cl
	507.1909	-0.9	-1.8	6.5	605.9	25.676	0.00	C22 H33 N2 O8 F Cl
	507.1909	-0.9	-1.8	4.5	603.2	22.924	0.00	C23 H39 O6 S3
	507.1891	0.9	1.8	11.5	588.2	7.973	0.03	C23 H28 N4 O8 F
	507.1889	1.1	2.2	7.5	598.0	17.760	0.00	C21 H32 N4 O7 Na S
	507.1913	-1.3	-2.6	10.5	597.4	17.197	0.00	C23 H31 N4 O7 S
	507.1914	-1.4	-2.8	11.5	605.7	25.501	0.00	C28 H33 O5 Na Cl
	507.1886	1.4	2.8	5.5	601.5	21.281	0.00	C23 H36 O7 F S2
	507.1885	1.5	3.0	1.5	603.5	23.246	0.00	C21 H40 O6 Na S3
	507.1885	1.5	3.0	3.5	606.2	25.935	0.00	C20 H34 N2 O8 F Na Cl
	507.1920	-2.0	-3.9	19.5	589.0	8.758	0.02	C31 H27 N2 O5

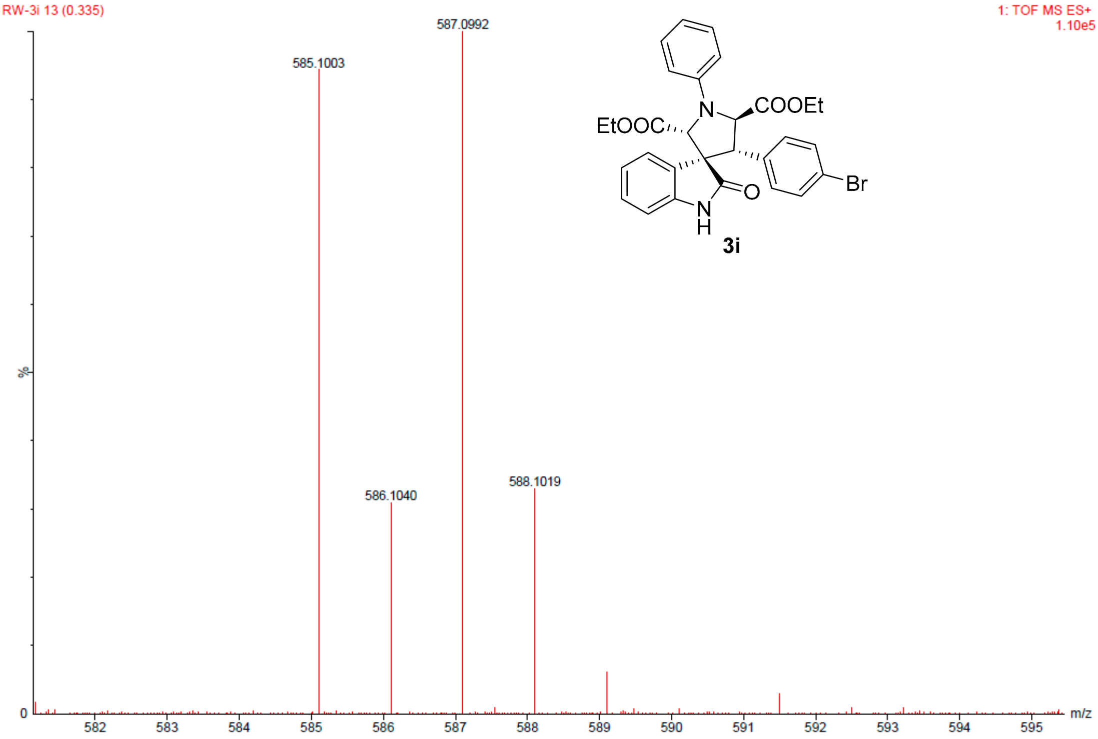

3i

Elemental Composition Report **Page 1**

Single Mass Analysis
Tolerance = 2.0 mDa / DBE: min = -1.5, max = 50.0
Element prediction: Off
Number of isotope peaks used for i-FIT = 3

Monoisotopic Mass, Even Electron Ions
830 formula(e) evaluated with 11 results within limits (up to 50 closest results for each mass)
Elements Used:
C: 20-40 H: 20-40 N: 0-6 O: 5-8 Na: 0-1 S: 0-4 Br: 0-1

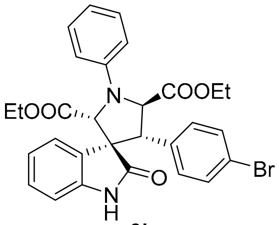

3i

RW-3i 13 (0.335)
1: TOF MS ES+

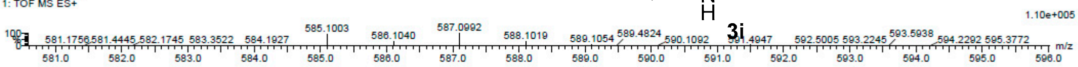

Minimum:				-1.5				
Maximum:		2.0	10.0	50.0				

Mass	Calc. Mass	mDa	PPM	DBE	i-FIT	Norm	Conf(%)	Formula
585.1003	585.1001	0.2	0.3	16.5	385.0	0.002	99.75	C29 H27 N2 O5 Na Br
	585.1008	-0.5	-0.9	25.5	407.2	22.161	0.00	C35 H21 O7 S
	585.0995	0.8	1.4	7.5	394.4	9.393	0.01	C21 H31 N4 O7 Na S Br
	585.1014	-1.1	-1.9	4.5	393.3	8.314	0.02	C23 H38 O6 S3 Br
	585.0991	1.2	2.1	18.5	403.6	18.546	0.00	C26 H22 N6 O5 Na S2
	585.1015	-1.2	-2.1	21.5	404.0	18.958	0.00	C28 H21 N6 O5 S2
	585.0990	1.3	2.2	1.5	396.2	11.166	0.00	C21 H39 O6 Na S3 Br
	585.1018	-1.5	-2.6	17.5	404.6	19.543	0.00	C30 H26 O7 Na S2
	585.1019	-1.6	-2.7	10.5	392.6	7.603	0.05	C23 H30 N4 O7 S Br
	585.0985	1.8	3.1	15.5	391.5	6.433	0.16	C26 H26 N4 O7 Br
	585.0984	1.9	3.2	22.5	406.8	21.801	0.00	C33 H22 O7 Na S

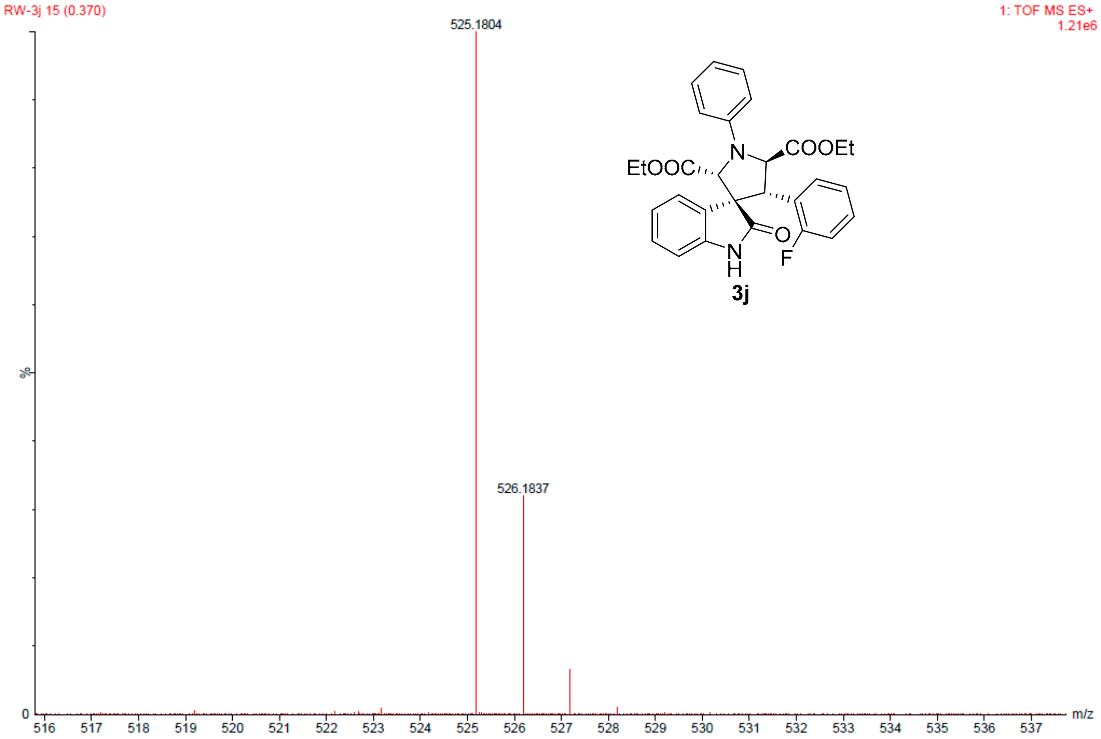

Elemental Composition Report **Page 1**

Single Mass Analysis
Tolerance = 2.0 mDa / DBE: min = -1.5, max = 50.0
Element prediction: Off
Number of isotope peaks used for i-FIT = 3

Monoisotopic Mass, Even Electron Ions
456 formula(e) evaluated with 4 results within limits (up to 50 closest results for each mass)
Elements Used:
C: 20-40 H: 20-40 N: 0-6 O: 5-8 F: 0-4 Na: 0-1

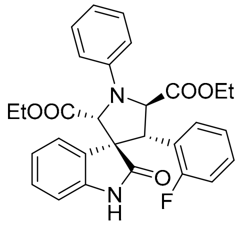

RW-3j 18 (0.440)
1: TOF MS ES+

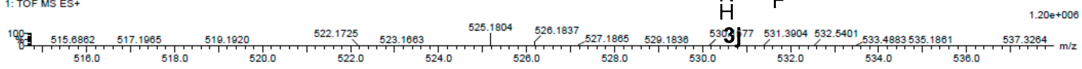

Minimum:				-1.5				
Maximum:		2.0	10.0	50.0				
Mass	Calc. Mass	mDa	PPM	DBE	i-FIT	Norm	Conf(%)	Formula
525.1804	525.1802	0.2	0.4	16.5	554.9	0.002	99.75	C29 H27 N2 O5 F Na
	525.1797	0.7	1.3	11.5	563.7	8.840	0.01	C23 H27 N4 O8 F2
	525.1813	-0.9	-1.7	12.5	561.2	6.314	0.18	C26 H28 N2 O6 F2 Na
	525.1786	1.8	3.4	15.5	562.4	7.531	0.05	C26 H26 N4 O7 F

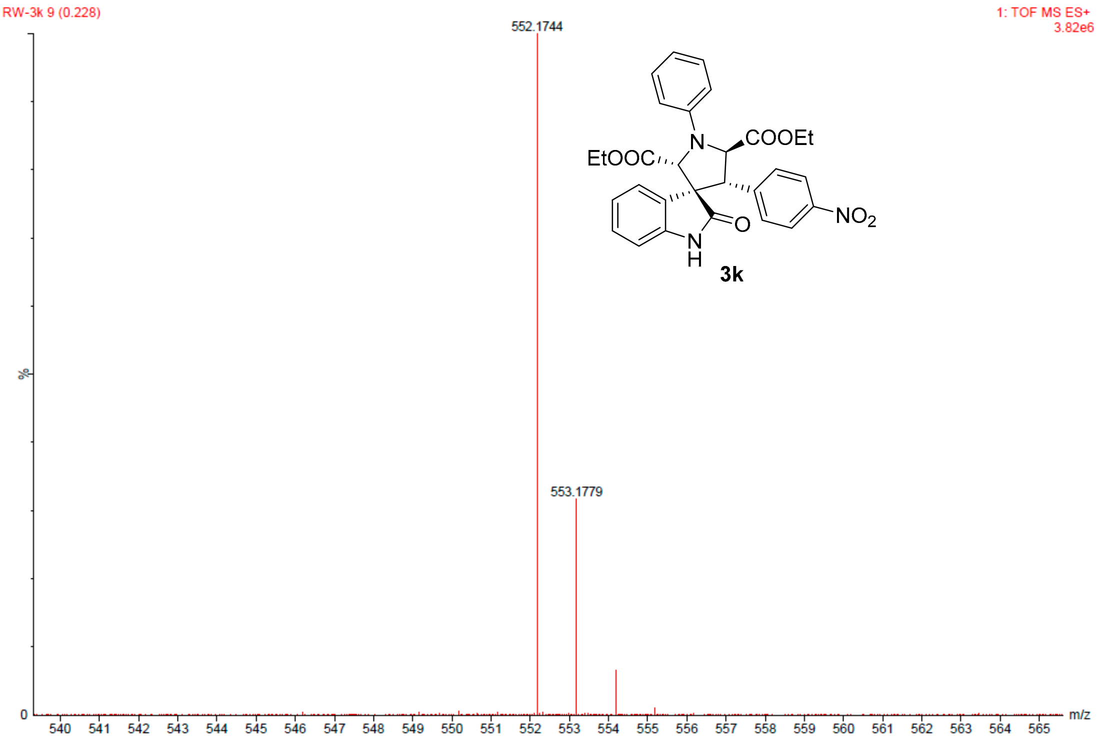

Elemental Composition Report **Page 1**

Single Mass Analysis
Tolerance = 2.0 mDa / DBE: min = -1.5, max = 50.0
Element prediction: Off
Number of isotope peaks used for i-FIT = 3

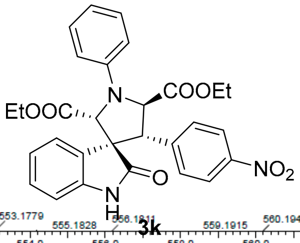

Monoisotopic Mass, Even Electron Ions
439 formula(e) evaluated with 4 results within limits (up to 50 closest results for each mass)
Elements Used:
C: 20-40 H: 20-40 N: 0-6 O: 5-8 Na: 0-1 S: 0-4
RW-3k 9 (0.228)
1: TOF MS ES+

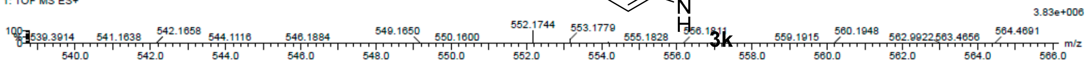

Mass	Calc. Mass	mDa	PPM	DBE	i-FIT	Norm	Conf(%)	Formula
Minimum:				-1.5				
Maximum:		2.0	10.0	50.0				
552.1744	552.1747	-0.3	-0.5	17.5	648.0	0.000	100.00	C29 H27 N3 O7 Na
	552.1735	0.9	1.6	2.5	668.9	20.907	0.00	C21 H39 N O8 Na S3
	552.1760	-1.6	-2.9	5.5	668.6	20.576	0.00	C23 H38 N O8 S3
	552.1726	1.8	3.3	10.5	667.0	18.995	0.00	C26 H34 N O8 S2

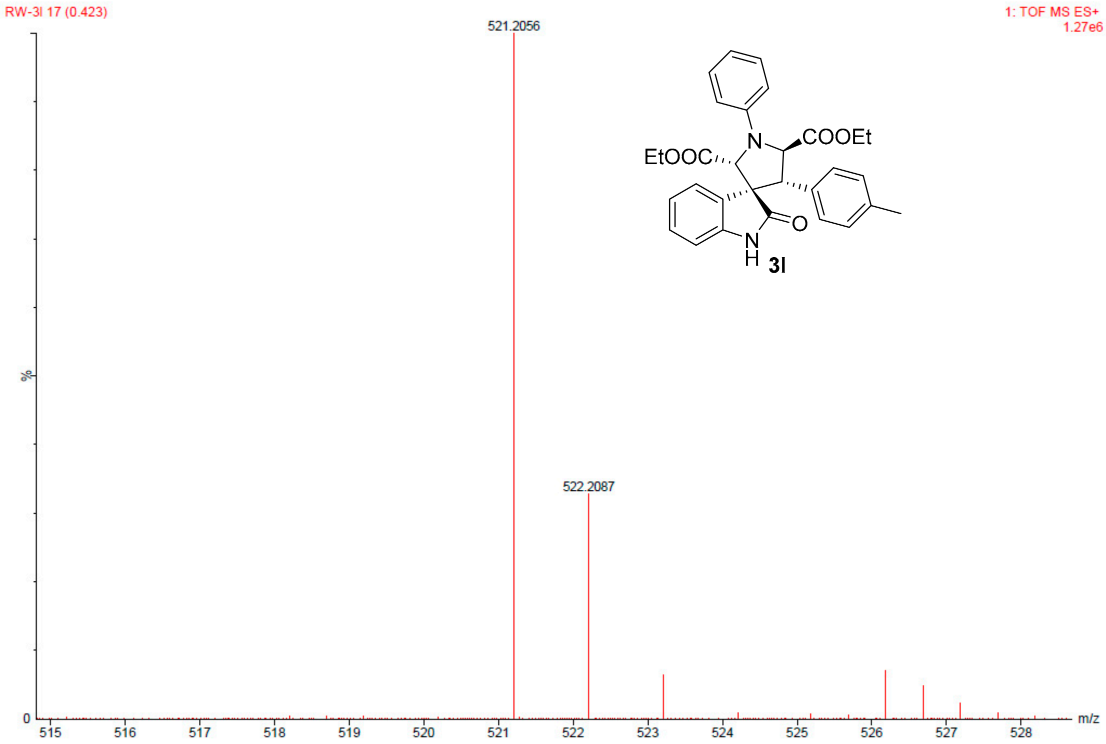

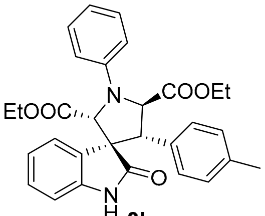

3l

Elemental Composition Report **Page 1**

Single Mass Analysis
Tolerance = 2.0 mDa / DBE: min = -1.5, max = 50.0
Element prediction: Off
Number of isotope peaks used for i-FIT = 3

Monoisotopic Mass, Even Electron Ions
385 formula(e) evaluated with 4 results within limits (up to 50 closest results for each mass)
Elements Used:
C: 20-40 H: 20-40 N: 0-6 O: 5-8 Na: 0-1 S: 0-4
RW-3l 17 (0.423)
1: TOF MS ES+

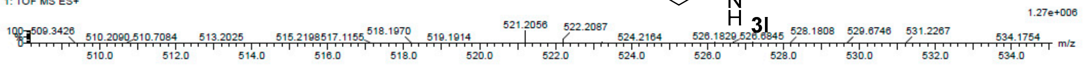

Minimum:				-1.5				
Maximum:		2.0	10.0	50.0				
Mass	Calc. Mass	mDa	PPM	DBE	i-FIT	Norm	Conf(%)	Formula
521.2056	521.2052	0.4	0.8	16.5	583.1	0.010	99.05	C30 H30 N2 O5 Na
	521.2046	1.0	1.9	7.5	600.4	17.313	0.00	C22 H34 N4 O7 Na S
	521.2070	-1.4	-2.7	10.5	599.7	16.652	0.00	C24 H33 N4 O7 S
	521.2036	2.0	3.8	15.5	587.8	4.660	0.95	C27 H29 N4 O7

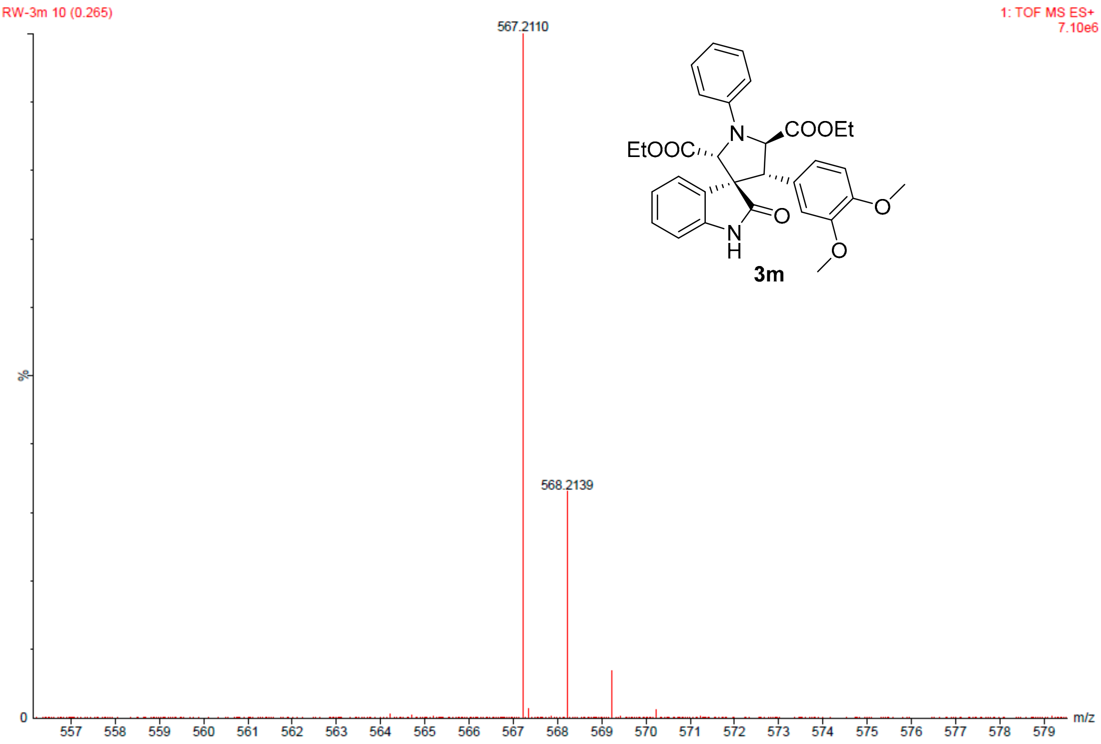

Elemental Composition Report **Page 1**

Single Mass Analysis
Tolerance = 2.0 mDa / DBE: min = -1.5, max = 50.0
Element prediction: Off
Number of isotope peaks used for i-FIT = 3

Monoisotopic Mass, Even Electron Ions
455 formula(e) evaluated with 2 results within limits (up to 50 closest results for each mass)
Elements Used:
C: 20-40 H: 20-40 N: 0-6 O: 5-8 Na: 0-1 S: 0-4
RW-3m 10 (0.265)
1: TOF MS ES+

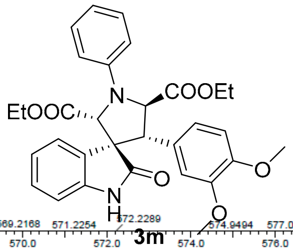

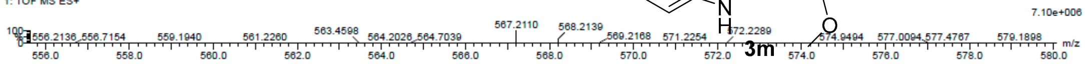

Minimum:				-1.5				
Maximum:		2.0	10.0	50.0				
Mass	Calc. Mass	mDa	PPM	DBE	i-FIT	Norm	Conf(%)	Formula
567.2110	567.2107	0.3	0.5	16.5	639.2	0.000	100.00	C31 H32 N2 O7 Na
	567.2093	1.7	3.0	5.5	658.6	19.352	0.00	C21 H39 N6 O6 S3

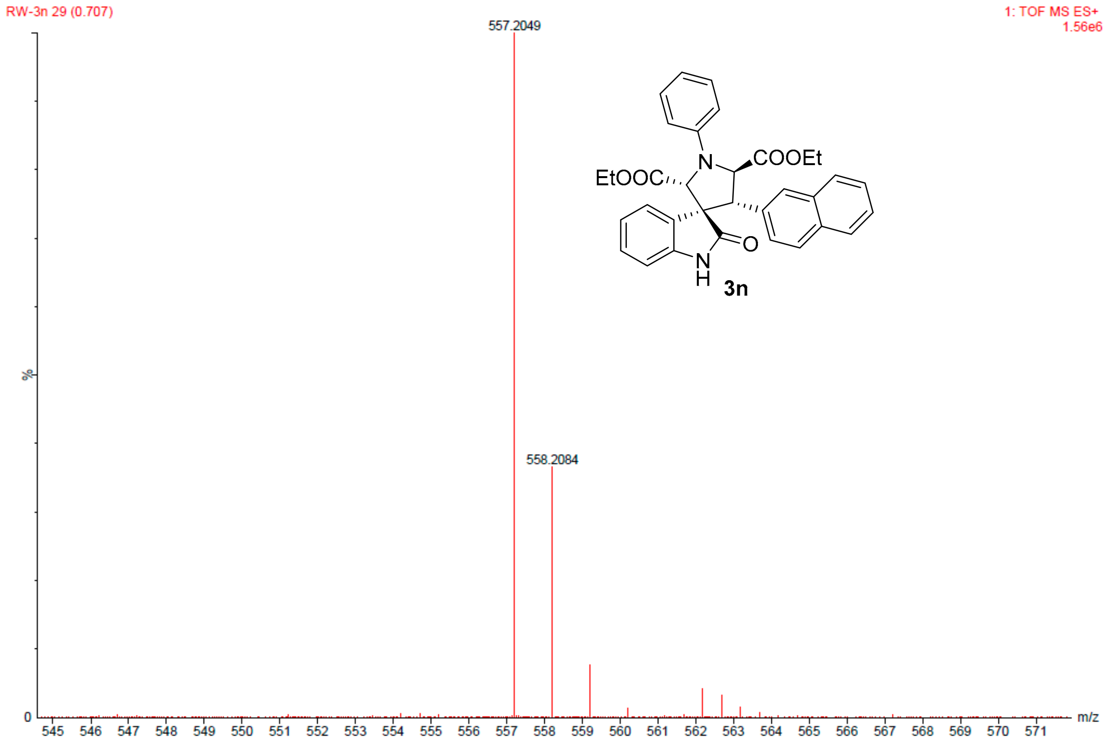

Elemental Composition Report **Page 1**

Single Mass Analysis
Tolerance = 2.0 mDa / DBE: min = -1.5, max = 50.0
Element prediction: Off
Number of isotope peaks used for i-FIT = 3

Monoisotopic Mass, Even Electron Ions
439 formula(e) evaluated with 4 results within limits (up to 50 closest results for each mass)
Elements Used:
C: 20-40 H: 20-40 N: 0-6 O: 5-8 Na: 0-1 S: 0-4

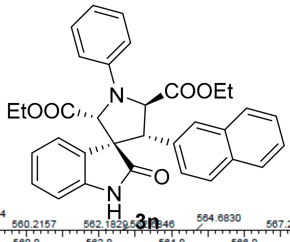

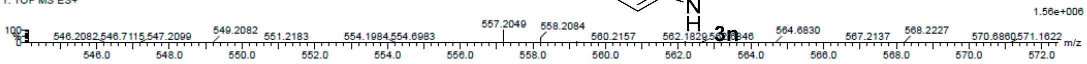

Minimum:				-1.5				
Maximum:		2.0	10.0	50.0				
Mass	Calc. Mass	mDa	PPM	DBE	i-FIT	Norm	Conf(%)	Formula
557.2049	557.2046	0.3	0.5	10.5	601.5	20.119	0.00	C25 H34 N4 O7 Na S
	557.2052	-0.3	-0.5	19.5	581.4	0.000	99.98	C33 H30 N2 O5 Na
	557.2036	1.3	2.3	18.5	590.2	8.758	0.02	C30 H29 N4 O7
	557.2032	1.7	3.1	12.5	605.2	23.779	0.00	C30 H37 O6 S2

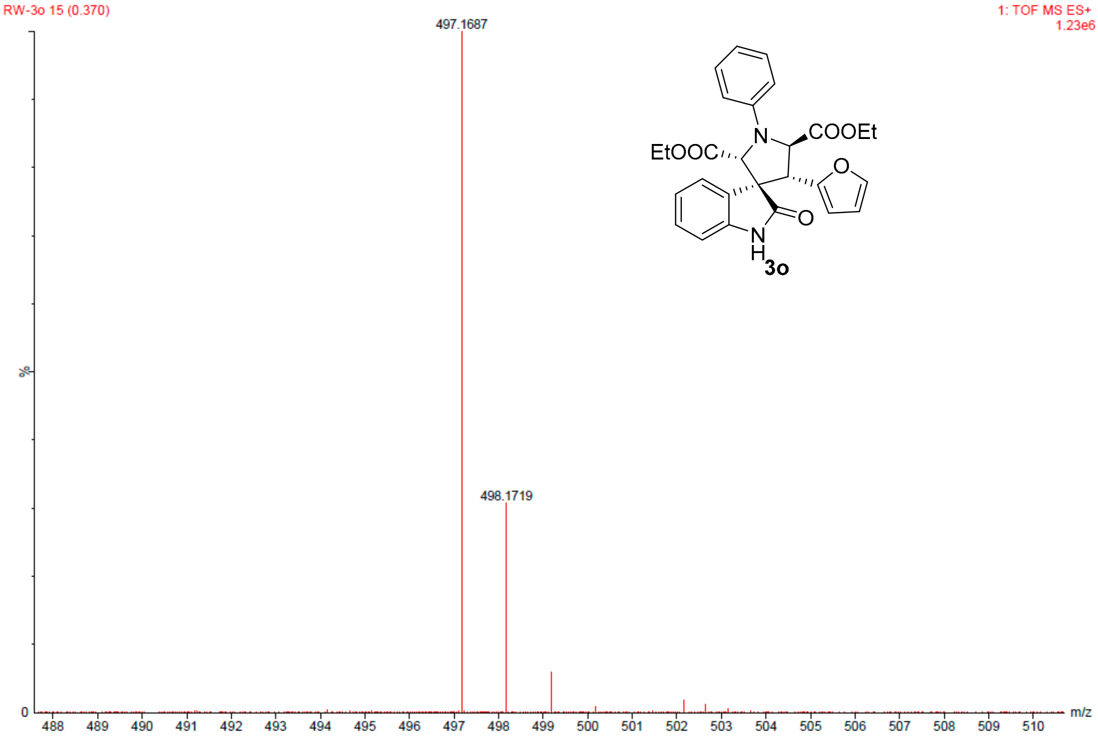

Elemental Composition Report **Page 1**

Single Mass Analysis
Tolerance = 2.0 mDa / DBE: min = -1.5, max = 50.0
Element prediction: Off
Number of isotope peaks used for i-FIT = 3

Monoisotopic Mass, Even Electron Ions
348 formula(e) evaluated with 5 results within limits (up to 50 closest results for each mass)
Elements Used:
C: 20-40 H: 20-40 N: 0-6 O: 5-8 Na: 0-1 S: 0-4
RW-3o 15 (0.370)
1: TOF MS ES+

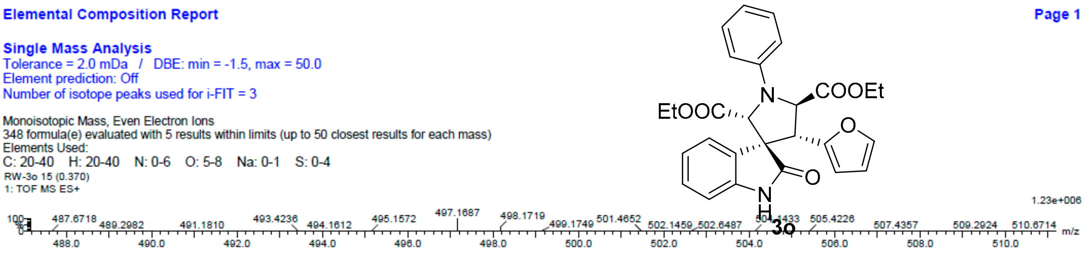

Mass	Calc. Mass	mDa	PPM	DBE	i-FIT	Norm	Conf(%)	Formula
Minimum:				-1.5				
Maximum:		2.0	10.0	50.0				
497.1687	497.1689	-0.2	-0.4	15.5	519.4	0.000	99.99	C27 H26 N2 O6 Na
	497.1701	-1.4	-2.8	3.5	545.0	25.595	0.00	C21 H37 O7 S3
	497.1672	1.5	3.0	14.5	529.0	9.593	0.01	C24 H25 N4 O8
	497.1668	1.9	3.8	8.5	543.4	23.926	0.00	C24 H33 O7 S2
	497.1706	-1.9	-3.8	9.5	539.5	20.045	0.00	C21 H29 N4 O8 S

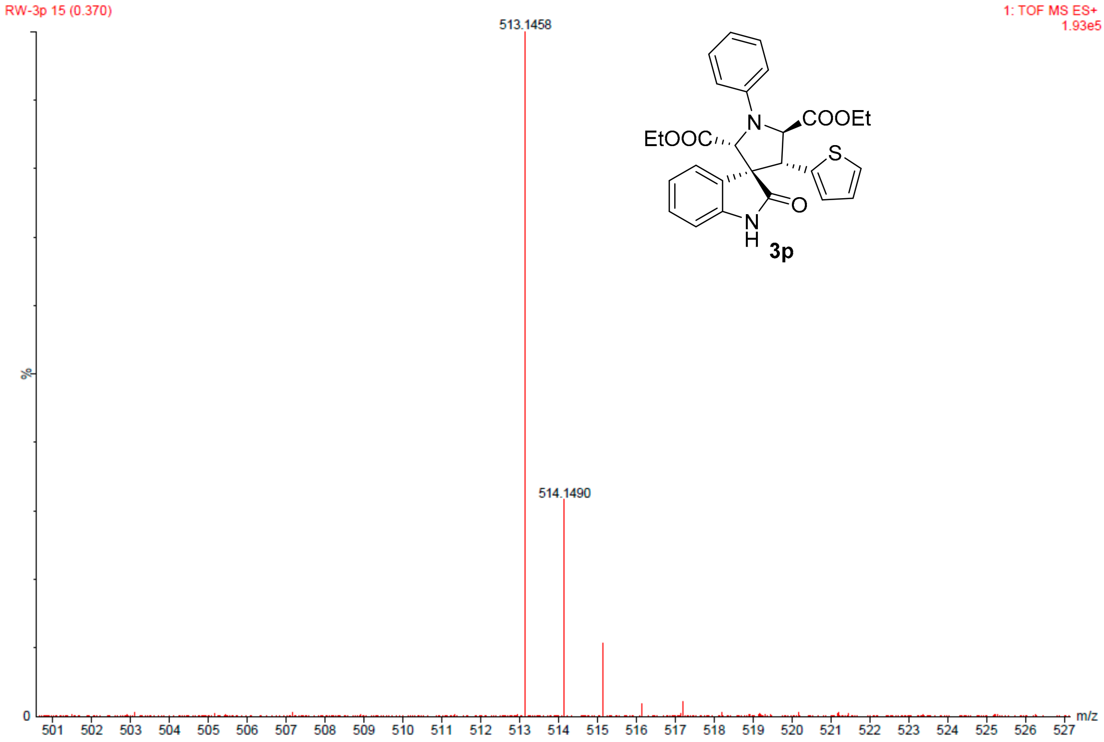

Elemental Composition Report **Page 1**

Single Mass Analysis
Tolerance = 2.0 mDa / DBE: min = -1.5, max = 50.0
Element prediction: Off
Number of isotope peaks used for i-FIT = 3

Monoisotopic Mass, Even Electron Ions
372 formula(e) evaluated with 6 results within limits (up to 50 closest results for each mass)
Elements Used:
C: 20-40 H: 20-40 N: 0-6 O: 5-8 Na: 0-1 S: 0-4
RW-3p 15 (0.370)
1: TOF MS ES+

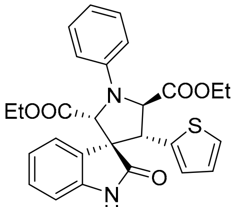

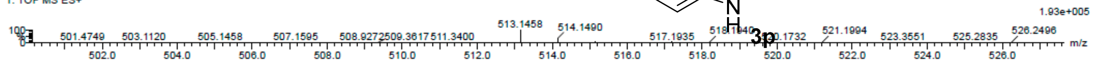

Minimum:				-1.5				
Maximum:		2.0	10.0	50.0				
Mass	Calc. Mass	mDa	PPM	DBE	i-FIT	Norm	Conf(%)	Formula
513.1458	513.1460	-0.2	-0.4	15.5	346.1	0.001	99.94	C27 H26 N2 O5 Na S
	513.1450	0.8	1.6	23.5	361.1	15.009	0.00	C32 H21 N2 O5
	513.1444	1.4	2.7	14.5	353.6	7.461	0.06	C24 H25 N4 O7 S
	513.1473	-1.5	-2.9	3.5	365.1	18.964	0.00	C21 H37 O6 S4
	513.1439	1.9	3.7	8.5	363.5	17.442	0.00	C24 H33 O6 S3
	513.1478	-2.0	-3.9	9.5	359.8	13.733	0.00	C21 H29 N4 O7 S2

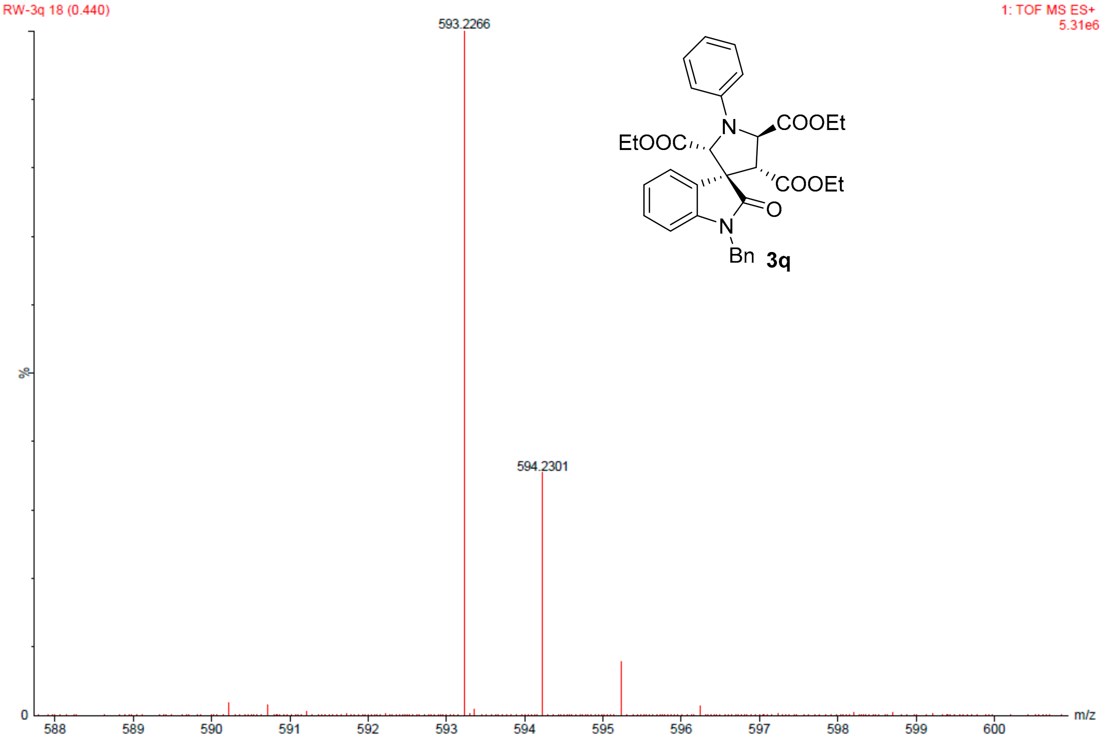

Elemental Composition Report **Page 1**

Single Mass Analysis
Tolerance = 2.0 mDa / DBE: min = -1.5, max = 50.0
Element prediction: Off
Number of isotope peaks used for i-FIT = 3

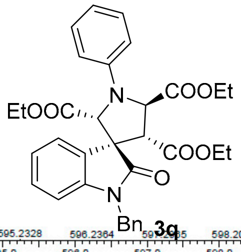

Monoisotopic Mass, Even Electron Ions
918 formula(e) evaluated with 4 results within limits (up to 50 closest results for each mass)
Elements Used:
C: 20-40 H: 20-40 N: 0-6 O: 5-8 Na: 0-1 S: 0-4 Cl: 0-1
RW-3q 18 (0.440)
1: TOF MS ES+

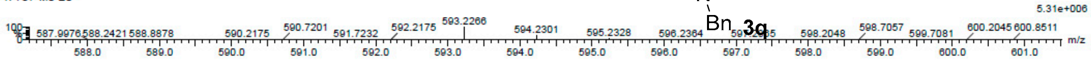

Minimum:				-1.5				
Maximum:		2.0	10.0	50.0				
Mass	Calc. Mass	mDa	PPM	DBE	i-FIT	Norm	Conf(%)	Formula
593.2266	593.2264	0.2	0.3	17.5	635.2	0.000	100.00	C33 H34 N2 O7 Na
	593.2255	1.1	1.9	13.5	657.8	22.620	0.00	C28 H35 N6 O5 Na Cl
	593.2279	-1.3	-2.2	16.5	657.4	22.226	0.00	C30 H34 N6 O5 Cl
	593.2282	-1.6	-2.7	12.5	657.5	22.270	0.00	C32 H39 O7 Na Cl

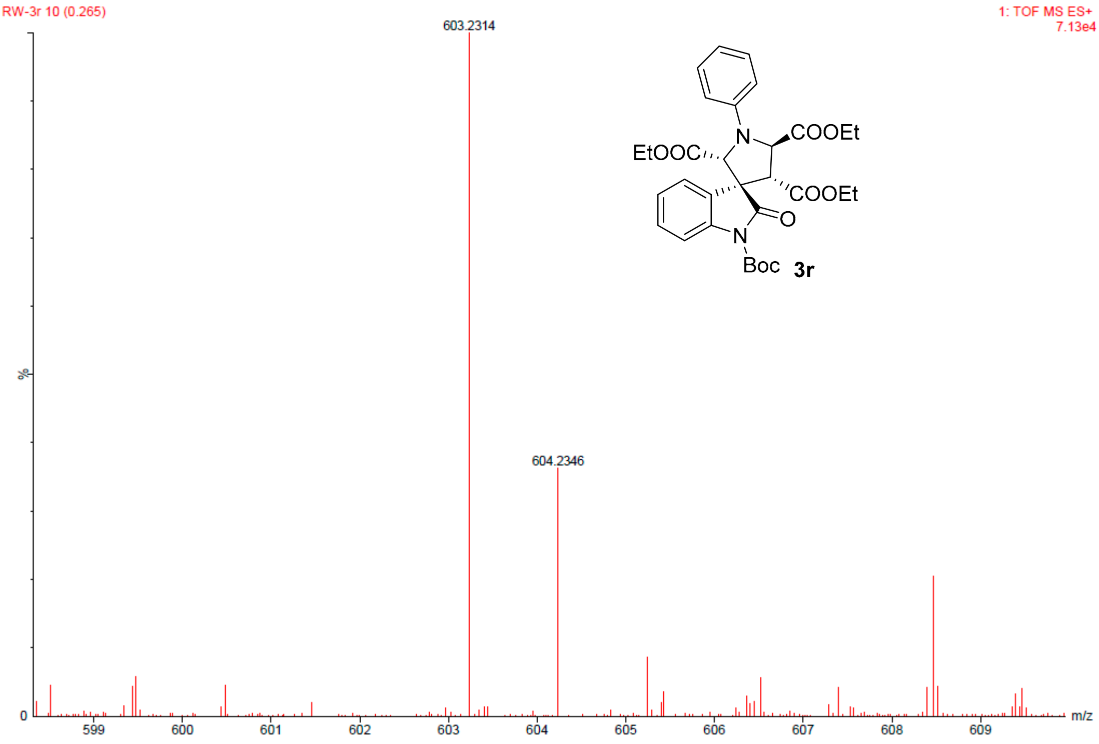

Elemental Composition Report **Page 1**

Single Mass Analysis
Tolerance = 2.0 mDa / DBE: min = -1.5, max = 50.0
Element prediction: Off
Number of isotope peaks used for i-FIT = 3

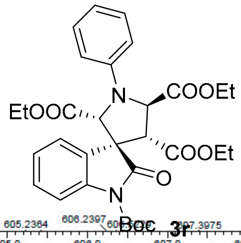

Monoisotopic Mass, Even Electron Ions
1118 formula(e) evaluated with 4 results within limits (up to 50 closest results for each mass)
Elements Used:
C: 20-40 H: 20-40 N: 0-6 O: 6-10 Na: 0-1 S: 0-4 Cl: 0-1
RW-3r 10 (0.265)
1: TOF MS ES+

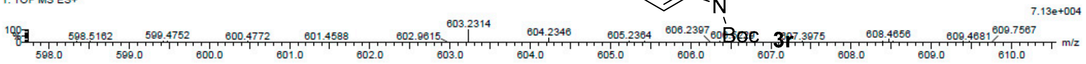

Mass	Calc. Mass	mDa	PPM	DBE	i-FIT	Norm	Conf(%)	Formula
Minimum:				-1.5				
Maximum:		2.0	10.0	50.0				
603.2314	603.2311	0.3	0.5	12.5	372.5	14.099	0.00	C29 H39 N4 O6 S2
	603.2310	0.4	0.7	10.5	378.2	19.806	0.00	C26 H37 N6 O7 Na Cl
	603.2319	-0.5	-0.8	14.5	358.4	0.000	100.00	C31 H36 N2 O9 Na
	603.2296	1.8	3.0	12.5	378.8	20.399	0.00	C31 H40 N2 O6 S Cl

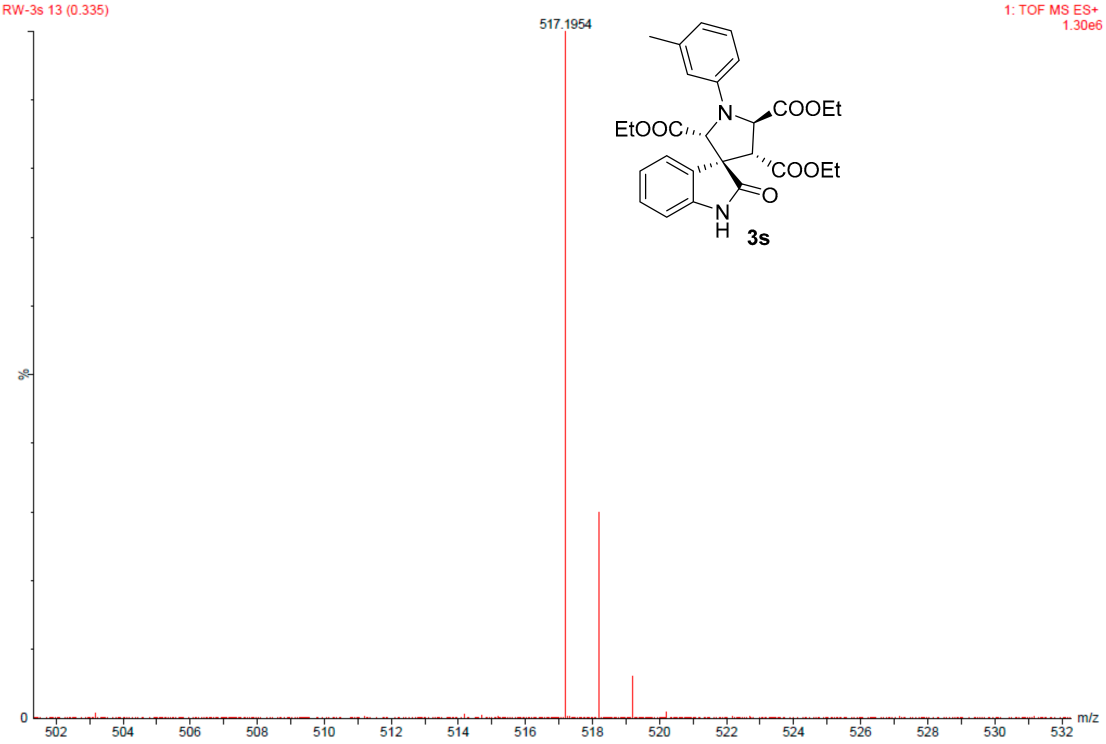

Elemental Composition Report **Page 1**

Single Mass Analysis
Tolerance = 2.0 mDa / DBE: min = -1.5, max = 50.0
Element prediction: Off
Number of isotope peaks used for i-FIT = 3

Monoisotopic Mass, Even Electron Ions
802 formula(e) evaluated with 5 results within limits (up to 50 closest results for each mass)
Elements Used:
C: 20-40 H: 20-40 N: 0-6 O: 6-10 Na: 0-1 S: 0-4 Cl: 0-1
RW-3s 13 (0.335)
1: TOF MS ES+

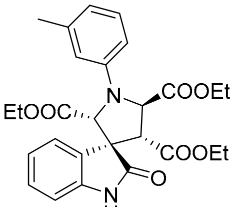

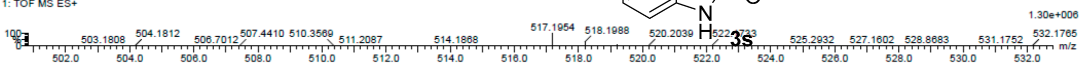

Minimum:				-1.5				
Maximum:		2.0	10.0	50.0				
Mass	Calc. Mass	mDa	PPM	DBE	i-FIT	Norm	Conf(%)	Formula
517.1954	517.1953	0.1	0.2	7.5	592.4	26.867	0.00	C23 H34 N2 O9 Cl
	517.1951	0.3	0.6	13.5	565.6	0.000	99.95	C27 H30 N2 O7 Na
	517.1968	-1.4	-2.7	7.5	584.1	18.531	0.00	C21 H33 N4 O9 S
	517.1969	-1.5	-2.9	8.5	592.2	26.700	0.00	C26 H35 O7 Na Cl
	517.1935	1.9	3.7	12.5	573.2	7.661	0.05	C24 H29 N4 O9

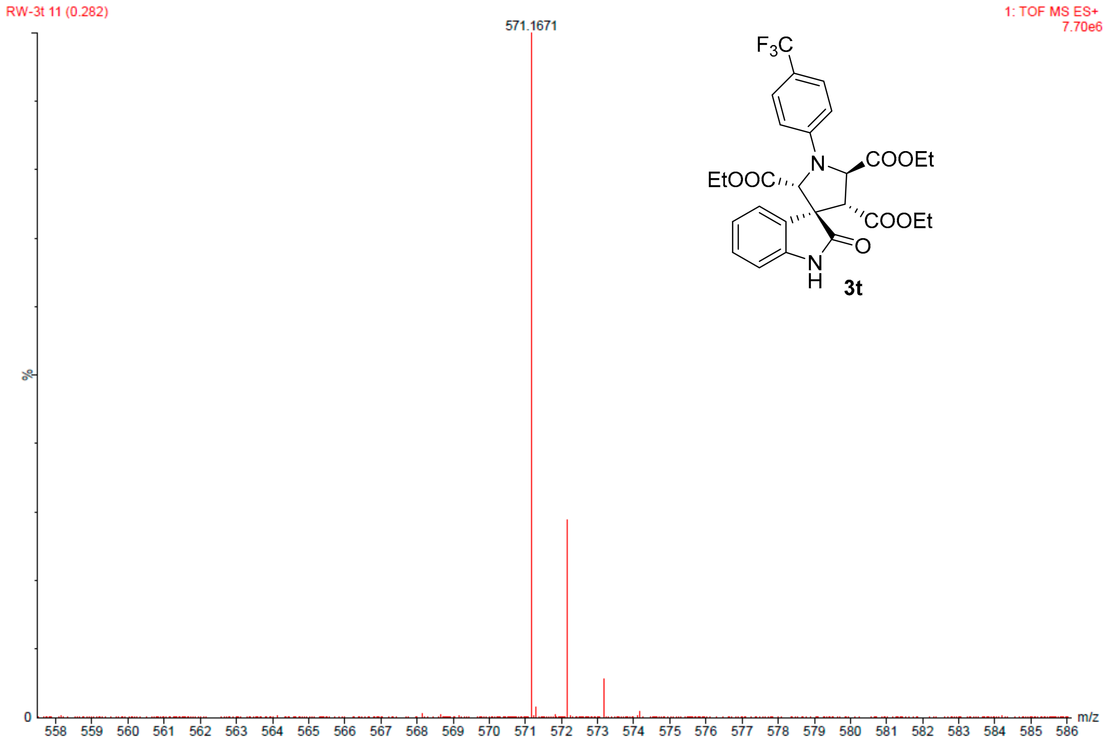

3t

Elemental Composition Report

Page 1

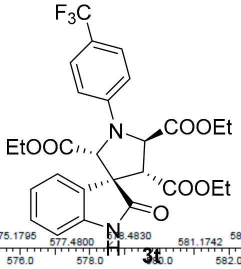

Single Mass Analysis

Tolerance = 2.0 mDa / DBE: min = -1.5, max = 50.0
Element prediction: Off
Number of isotope peaks used for i-FIT = 3

Monoisotopic Mass, Even Electron Ions
2225 formula(e) evaluated with 19 results within limits (up to 50 closest results for each mass)
Elements Used:
C: 20-40 H: 20-40 N: 0-3 O: 6-10 Na: 0-1 S: 0-4 Cl: 0-1 F: 0-3

RW-3t 11 (0.282)
1: TOF MS ES+

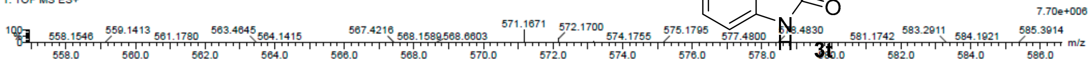

Minimum:				-1.5				
Maximum:		2.0	10.0	50.0				

Mass	Calc. Mass	mDa	PPM	DBE	i-FIT	Norm	Conf(%)	Formula
571.1671	571.1672	-0.1	-0.2	9.5	689.5	17.521	0.00	C26 H35 O10 S2
	571.1670	0.1	0.2	7.5	693.9	21.879	0.00	C23 H31 N2 O9 Cl F3
	571.1670	0.1	0.2	14.5	694.1	22.064	0.00	C29 H32 N2 O6 S Cl
	571.1669	0.2	0.4	5.5	691.3	19.316	0.00	C24 H37 O7 S3 F2
	571.1668	0.3	0.5	3.5	694.3	22.316	0.00	C21 H35 N2 O8 Na S Cl F2
	571.1668	0.3	0.5	13.5	672.0	0.007	99.31	C27 H27 N2 O7 Na F3
	571.1675	-0.4	-0.7	12.5	693.7	21.713	0.00	C29 H31 O6 Na Cl F2
	571.1679	-0.8	-1.4	6.5	694.4	22.433	0.00	C24 H37 N2 O6 Na S2 Cl
	571.1681	-1.0	-1.8	20.5	678.0	6.035	0.24	C32 H25 N2 O6 F2
	571.1681	-1.0	-1.8	10.5	694.1	22.065	0.00	C26 H33 N2 O7 S Cl F
	571.1681	-1.0	-1.8	1.5	691.3	19.322	0.00	C21 H38 O8 S3 F3
	571.1681	-1.0	-1.8	1.5	691.4	19.358	0.00	C21 H40 O10 Na S3
	571.1659	1.2	2.1	11.5	693.9	21.933	0.00	C26 H30 N2 O8 Cl F2
	571.1658	1.3	2.3	9.5	691.4	19.377	0.00	C27 H36 O6 S3 F
	571.1657	1.4	2.5	7.5	694.4	22.386	0.00	C24 H34 N2 O7 Na S Cl F
	571.1657	1.4	2.5	17.5	677.4	5.393	0.45	C30 H26 N2 O6 Na F2
	571.1686	-1.5	-2.6	8.5	693.7	21.744	0.00	C26 H32 O7 Na Cl F3
	571.1690	-1.9	-3.3	12.5	685.7	13.739	0.00	C27 H30 N2 O6 Na S F2
	571.1691	-2.0	-3.5	2.5	694.4	22.441	0.00	C21 H38 N2 O7 Na S2 Cl F

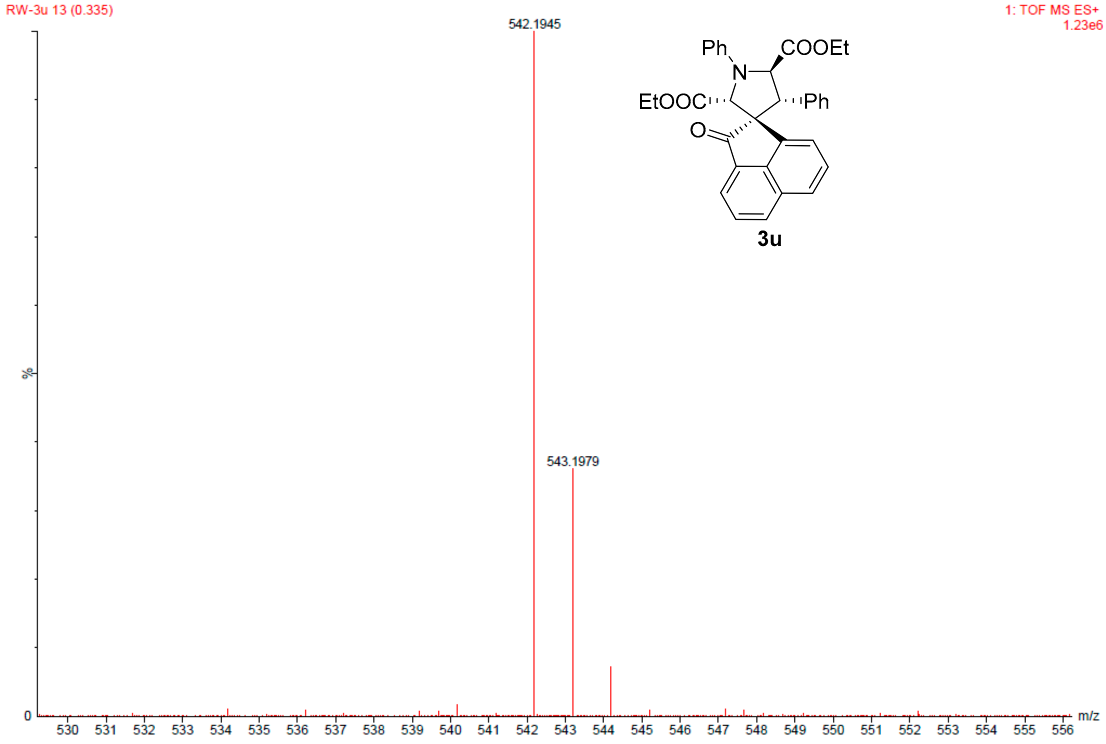

Elemental Composition Report Page 1

Single Mass Analysis
Tolerance = 2.0 mDa / DBE: min = -1.5, max = 50.0
Element prediction: Off
Number of isotope peaks used for i-FIT = 3

Monoisotopic Mass, Even Electron Ions
1439 formula(e) evaluated with 8 results within limits (up to 50 closest results for each mass)
Elements Used:
C: 20-40 H: 20-40 N: 0-2 O: 4-7 F: 0-3 Na: 0-1 S: 0-4 Cl: 0-1
RW-3u 13 (0.335)
1: TOF MS ES+

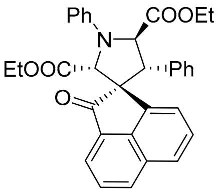

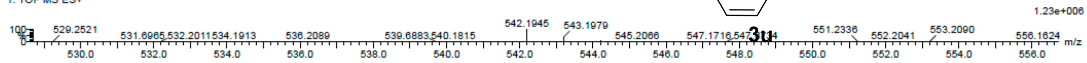

	Calc. Mass	mDa	PPM	DBE	i-FIT	Norm	Conf(%)	Formula
Minimum:				-1.5				
Maximum:		2.0	10.0	50.0				

Mass	Calc. Mass	mDa	PPM	DBE	i-FIT	Norm	Conf(%)	Formula
542.1945	542.1946	-0.1	-0.2	13.5	517.2	25.414	0.00	C29 H33 N O7 Cl
	542.1943	0.2	0.4	9.5	517.7	25.905	0.00	C27 H35 N O4 F2 S Cl
	542.1943	0.2	0.4	19.5	491.8	0.006	99.37	C33 H29 N O5 Na
	542.1953	-0.8	-1.5	1.5	518.1	26.293	0.00	C22 H40 N O4 F2 Na S2 Cl
	542.1955	-1.0	-1.8	5.5	517.7	25.886	0.00	C24 H38 N O7 Na S Cl
	542.1955	-1.0	-1.8	5.5	517.7	25.910	0.00	C24 H36 N O5 F3 S Cl
	542.1955	-1.0	-1.8	15.5	496.9	5.071	0.63	C30 H30 N O6 F Na
	542.1931	1.4	2.6	2.5	518.0	26.221	0.00	C22 H37 N O5 F3 Na S Cl

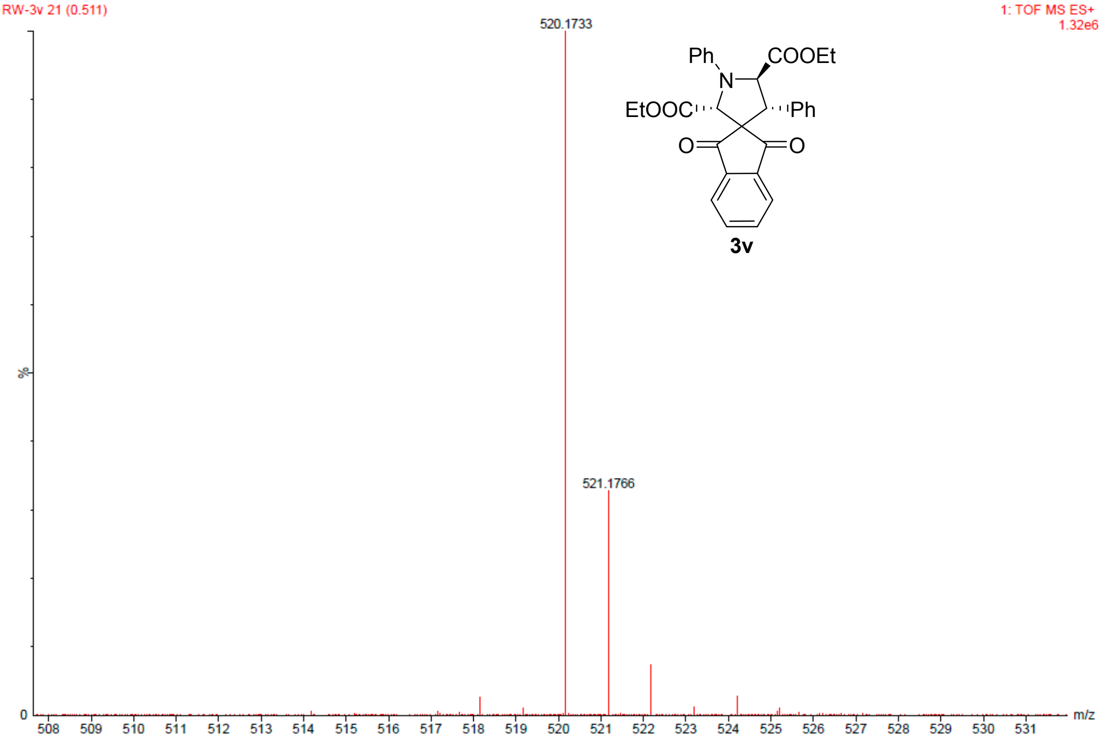

Elemental Composition Report

Page 1

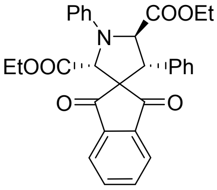

3v

Single Mass Analysis
Tolerance = 2.0 mDa / DBE: min = -1.5, max = 50.0
Element prediction: Off
Number of isotope peaks used for i-FIT = 3

Monoisotopic Mass, Even Electron Ions
797 formula(e) evaluated with 3 results within limits (up to 50 closest results for each mass)
Elements Used:
C: 25-40 H: 20-40 N: 0-2 O: 4-7 F: 0-2 Na: 0-1 S: 0-4 Cl: 0-1
RW-3v 21 (0.511)
1: TOF MS ES+

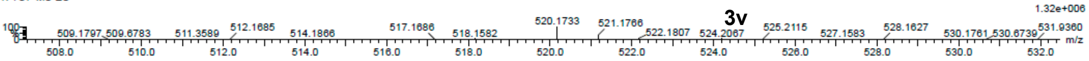

Minimum:				-1.5				
Maximum:		2.0	10.0	50.0				
Mass	Calc. Mass	mDa	PPM	DBE	i-FIT	Norm	Conf(%)	Formula
520.1733	520.1736	-0.3	-0.6	17.5	536.8	0.015	98.48	C30 H27 N O6 Na
	520.1725	0.8	1.5	11.5	558.3	21.545	0.00	C27 H32 N O4 F S Cl
	520.1748	-1.5	-2.9	13.5	541.0	4.186	1.52	C27 H28 N O7 F Na

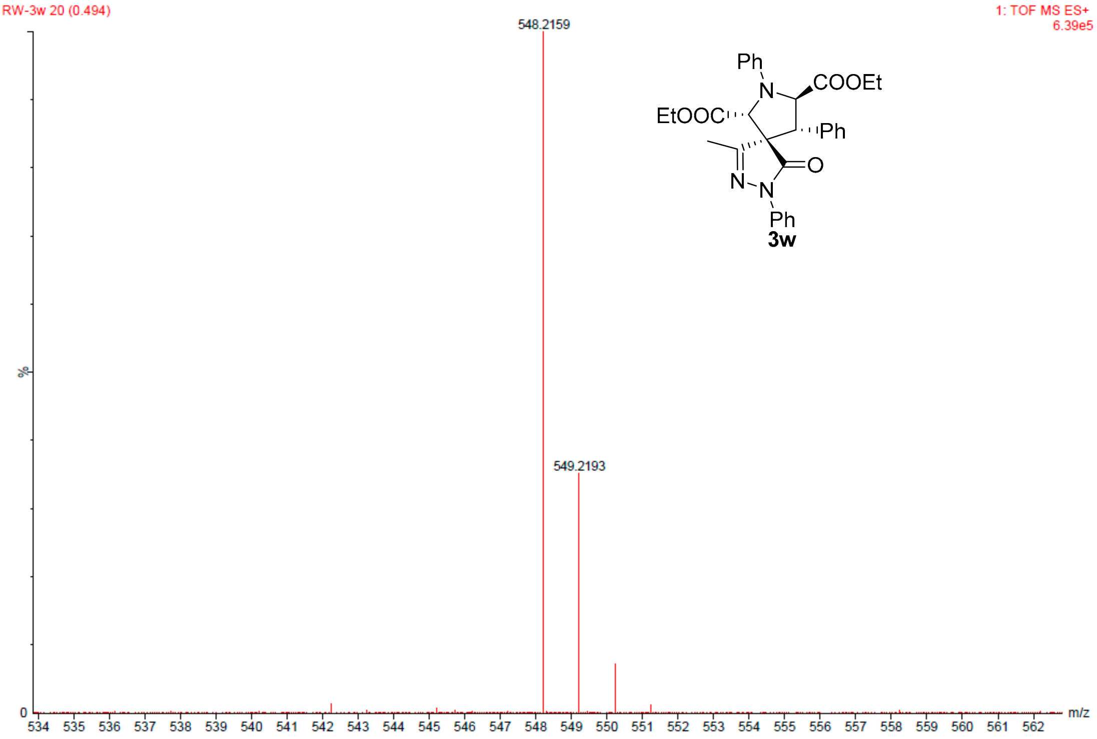

Elemental Composition Report **Page 1**

Single Mass Analysis
Tolerance = 2.0 mDa / DBE: min = -1.5, max = 50.0
Element prediction: Off
Number of isotope peaks used for i-FIT = 3

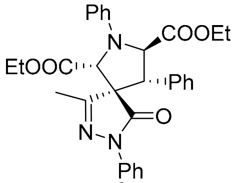

3w

Monoisotopic Mass, Even Electron Ions
367 formula(e) evaluated with 4 results within limits (up to 50 closest results for each mass)
Elements Used:
C: 20-35 H: 20-40 N: 0-5 O: 0-7 F: 0-2 Na: 0-1
RW-3w 20 (0.494)
1: TOF MS ES+

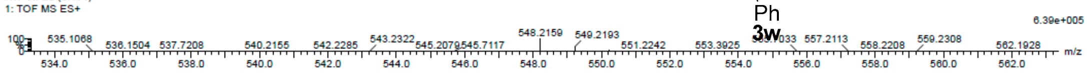

Minimum:				-1.5				
Maximum:		2.0	10.0	50.0				
Mass	Calc. Mass	mDa	PPM	DBE	i-FIT	Norm	Conf(%)	Formula
548.2159	548.2161	-0.2	-0.4	17.5	486.8	0.000	99.98	C31 H31 N3 O5 Na
	548.2150	0.9	1.6	21.5	495.9	9.116	0.01	C34 H28 N3 O2 F2
	548.2173	-1.4	-2.6	13.5	497.5	10.691	0.00	C28 H32 N3 O6 F Na
	548.2145	1.4	2.6	16.5	497.3	10.467	0.00	C28 H30 N5 O7

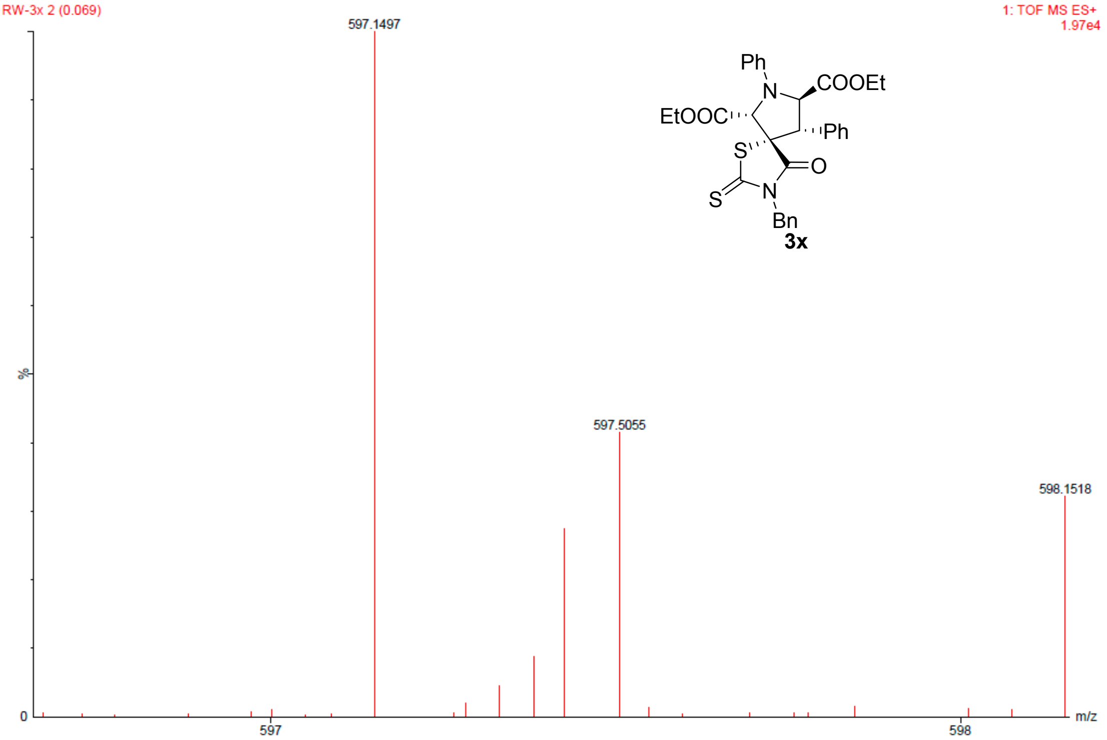

Elemental Composition Report Page 1

Single Mass Analysis
Tolerance = 2.0 mDa / DBE: min = -1.5, max = 50.0
Element prediction: Off
Number of isotope peaks used for i-FIT = 3

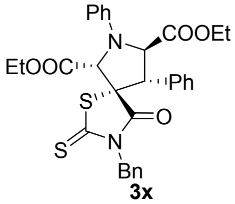

Monoisotopic Mass, Even Electron Ions
580 formula(e) evaluated with 2 results within limits (up to 50 closest results for each mass)
Elements Used:
C: 30-32 H: 30-35 N: 0-4 O: 5-8 Na: 0-1 S: 1-2 Cl: 0-2 Br: 0-2

RW-3x 2 (0.069)
1: TOF MS ES+

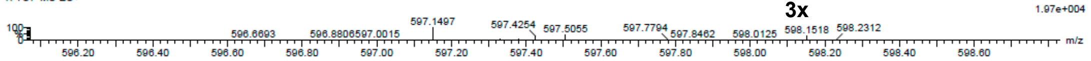

Mass	Calc. Mass	mDa	PPM	DBE	i-FIT	Norm	Conf(%)	Formula
Minimum:				-1.5				
Maximum:		2.0	10.0	50.0				
597.1497	597.1494	0.3	0.5	17.5	205.9	0.056	94.58	C31 H30 N2 O5 Na S2
	597.1512	-1.5	-2.5	12.5	208.8	2.914	5.42	C30 H35 O5 Na S2 Cl
